# Epigenetic Silencing of RFX7 Defines a Transcriptional Axis Linking Lactate Metabolism to Immune Checkpoint Therapy in Glioblastoma

**DOI:** 10.1002/advs.202523792

**Published:** 2026-05-28

**Authors:** Liying Han, Jinpeng Zhou, Gang Zhu, Fan Chen, Leiyang Li, Wenxing Cui, Fang Sun, Tian Feng, Yue Zhang, Qiang Wang, Shuoyao Ma, Chengxuan Guo, Ziwen Zhang, Kai Wang, Jiahui Wang, Liangbo Wang, Wencong Li, Chenyi Yuan, Haixiao Liu, Liang Wang, Yan Qu

**Affiliations:** ^1^ Department of Neurosurgery Tangdu Hospital The Fourth Military Medical University Xi'an Shaanxi China; ^2^ Department of Neurosurgery Air Force Hospital of Eastern Theater Nanjing Jiangsu China

**Keywords:** glioma, H4K12la, immune infiltration, lactate metabolism, PI3K/AKT pathway, RFX7‐PIK3IP1, Stiripentol

## Abstract

Glioblastoma (GBM) is a highly aggressive brain tumor characterized by rapid proliferation, diffuse invasion, and robust immunosuppression. Although excessive aerobic glycolysis and lactate accumulation are known to contribute to an immunosuppressive microenvironment, the upstream transcriptional mechanisms connecting metabolic reprogramming and immunotherapy resistance in GBM remain unclear.

By integrating transcriptomic profiling, chromatin immunoprecipitation sequencing, and metabolic analysis with gene perturbation experiments, we identified a regulatory axis comprising RFX7 and its downstream target PIK3IP1. In GBM tissues, RFX7 expression was reduced due to promoter hypermethylation. Restoration of RFX7 enhanced PIK3IP1 expression, suppressed PI3K/AKT activation, and inhibited malignant progression in GBM. Loss of PIK3IP1 increased lactate production and histone H4K12 lactylation (H4K12la), coinciding with upregulation of PD‐L1 and CSF1 and enhanced tumor immunosuppressive features. Pharmacological inhibition of lactate production with Stiripentol reduced H4K12la level, intracranial tumor growth, and immunosuppressive cell infiltration, while improving survival and response to immune checkpoint based therapy in experimental models.

These findings identify an upstream transcriptional pathway linking lactate metabolism, histone lactylation, and immune suppression in GBM. Targeting the RFX7‐PIK3IP1 axis provides a mechanistic rationale for metabolic‐immune modulation in therapy, addressing an aspect that has remained insufficiently understood in GBM immune resistance.

## Graphical abstract

Glioblastoma (GBM) exhibits RFX7 silencing via promoter hypermethylation, which downregulates PIK3IP1 to activate PI3K/AKT signaling, increase lactate accumulation, and enhance histone H4K12 lactylation. This lactylation upregulates PD‐L1/CSF1 expression and drives the immunosuppressive microenvironment formation. Pharmacological inhibition of lactate production by Stiripentol reduces H4K12la, reverses immunosuppression, and potentiates antitumor immunity in GBM.

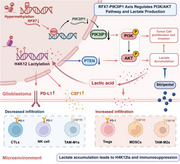

## 1 Introduction

Glioblastoma multiforme (GBM) is the most aggressive malignant glioma in the central nervous system. Despite advances in surgical resection, chemotherapy, and radiotherapy, median survival of GBM patients remains approximately 12–15 months [1, 2]. A major contributor to this poor prognosis is the presence of an immunosuppressive tumor microenvironment (TME), which reduces the effectiveness of immunotherapy such as immune checkpoint inhibition [3, 4, 5]. The success of such therapies depends on the function and infiltration of cytotoxic immune cells, which can be impaired by tumor‐driven metabolic changes [6, 7].

Altered tumor metabolism is a recognized feature of GBM and has been implicated in immune evasion [8]. GBM cells frequently adopt aerobic glycolysis, resulting in elevated lactate production [9]. Lactate not only alters the biochemical and cellular properties of the TME but also promotes immune suppression by impairing cytotoxic T cell and NK cell activity and by upregulating immunosuppressive molecules such as PD‐L1 [10, 11]. These changes can reduce the efficacy of immune checkpoint blockade. In addition, lactate has been associated with epigenetic modifications that may affect transcriptional programs relevant to tumor‐immune interactions [12, 13, 14]. However, in GBM, the upstream regulatory factors that promote lactate accumulation and coordinate its effects on immune suppression remain incompletely understood.

The Regulatory Factor X (RFX) family of transcription factors, through their highly conserved DNA‐binding domains, participate in various biological processes and play significant roles in tumors and immune responses [15, 16, 17]. In tumor‐related studies, RFX1 functions as a tumor suppressor, and its overexpression effectively suppresses the malignant phenotype of GBM cells [18]. Moreover, RFX6 modulates aerobic glycolysis in hepatocellular carcinoma cells and supports tumor growth [19]. Regarding immune regulation, the RFX complex formed by RFX5 is essential for the transcription of MHC class II molecules; its deficiency leads to immunodeficiency disorders [20, 21]. Additionally, RFX7 is indispensable for maintaining the homeostasis and function of natural killer (NK) cells [22, 23]. However, the effects of RFX7 on tumor growth, metabolism, and immune regulation in GBM remain to be determined.

The present study examines the role of RFX7 in GBM, with specific attention to its relationship with lactate metabolism and the immune TME. Using public datasets, primary glioma samples, and experimental models, we assessed the association between RFX7 expression and tumor cell behaviors, metabolic characteristics and immune composition. We further evaluated whether pharmacological inhibition of lactate production could alter these features and improve responses to immune‐based therapies.

## 2 Result

### 2.1 RFX7 Expression is Downregulated in GBM via Promoter Hypermethylation

**FIGURE 1:**
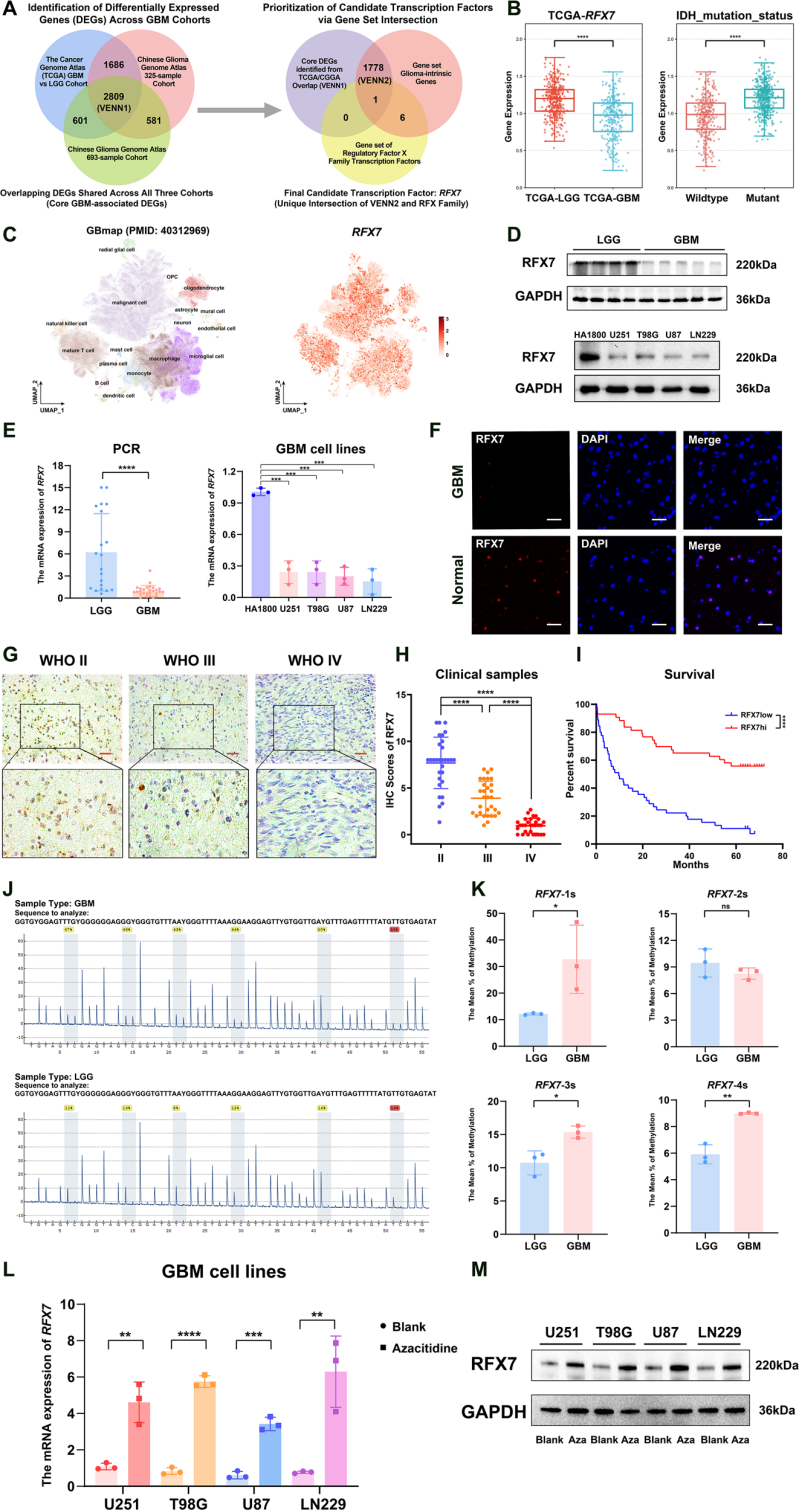
RFX7 Expression is Downregulated in GBM via Promoter Hypermethylation. (A) The intersection of DEGs from TCGA and CGGA datasets (VENN1), and its overlap with glioma‐intrinsic genes and the RFX gene family. (B) Expression of *RFX7* in LGG/GBM and IDH wildtype/mutant gliomas in TCGA database (Student's *t*‐test). (C) UMAP visualization of the *RFX7* expression in GBmap single‐cell atlas. (D, E) WB (D) and qPCR (E) analyses of the expression of RFX7 in LGG and GBM tissues, and in the normal astrocyte cell line and GBM cell lines (qPCR: Student's *t*‐test; 20 LGG and 27 GBM samples; n = 3 per cell line). (F) Immunofluorescence staining of the expression of RFX7 in GBM tissues and adjacent normal tissues (Scale Bar = 20 µm). (G, H) IHC staining (G) and analysis (H) of the expression of RFX7 in gliomas with different grades (Scale Bar = 50 µm, Kruskal–Wallis test; n = 30 per group; mean value of IHC scores: II: 7.689; III: 3.9; IV: 0.954). (I) Kaplan–Meier survival curves of the prognosis of glioma patients with different RFX7 expression (Log‐rank test). (J) Schematic diagram of the CpG islands and the specific region analyzed by bisulfite pyrosequencing within the *RFX7* promoter region. (K) The methylation levels of the designed *RFX7* promoter CpG island sites in LGG and GBM tissues (Student's *t*‐test; n = 3 per group). (L, M) The mRNA (L) and protein (M) expression of RFX7 in GBM cell lines after the treatment of Azacitidine (Student's *t*‐test; n = 3 per cell line). All values are shown as the mean ± SD. ^*^ *p* < 0.05, ^**^ *p* < 0.01, ^***^ *p* < 0.001, ^****^ *p* < 0.0001, ns: not significant.

To identify potential upstream transcription regulators driving GBM progression, we performed differential expression analysis based on GBM versus low‐grade glioma (LGG) expression profiles from the Cancer Genome Atlas (TCGA) and Chinese Glioma Genome Atlas (CGGA) databases, identifying 2,909 differentially expressed genes (DEGs) (VENN1). Further intersection of the VENN1 with the glioma‐intrinsic gene set [24] yielded 1,779 DEGs (VENN2). Given the critical role of RFX family transcription factors in tumor proliferation and immune regulation, we intersected the VENN2 with RFX family members, ultimately identifying *RFX7* as the candidate gene (Figure 1A). While RFX7's tumor‐suppressive functions have been confirmed in other cancer types [25, 26, 27, 28], its regulatory mechanisms in GBM remain largely unexplored.

Analysis of the TCGA and CGGA (merged by CGGA‐693 and CGGA‐325) databases revealed significantly lower *RFX7* expression in GBM compared with LGG. Notably, *RFX7* was markedly downregulated in IDH‐wildtype gliomas, a highly aggressive subtype with poor prognosis, suggesting that reduced expression may correlate with adverse patient outcomes (Figure 1B, Figure S1A) [29, 30, 31]. We analyzed the cellular expression of *RFX7* in two public single‐cell GBM databases: GBmap [32] and CNP0003766 [33]. Results showed that *RFX7* ‐was highly expressed in malignant/GBM cells across both datasets (Figure 1C and Figure S1B). We further examined *RFX7* expression patterns across GBmap‐defined cell populations, stratified by TCGA expression levels. At the malignant cell state levels, the *RFX7*‐low group was significantly enriched in the mesenchymal‐like (MES‐like) state, including hypoxia and MHC‐enriched subtypes, which are highly associated with immunosuppression and therapeutic resistance in GBM [32]. Notably, *RFX7*‐associated enrichment differences were further observed across the signature subtypes of the neural progenitor cell‐like (NPC‐like), astrocyte‐like (AC‐like), and oligodendrocyte precursor cell‐like (OPC‐like) states (Figure S1C, D). Integrated analyses using ESTIMATE, CIBERSORT, and CIBERSORTx with GBmap cell type atlas revealed that gliomas in the low‐expression group were associated with reduced tumor purity and a profoundly immune‐infiltrated microenvironment, including elevated macrophage and stromal cell scores (Figure S1E–I). The high expression of *RFX7* was correlated with better prognosis of glioma patients in the datasets examined (Figure S1J, K).

We further measured RFX7 protein and mRNA in fresh‐frozen clinical specimens from Tangdu Hospital. Western blot and qPCR analyses confirmed significantly lower RFX7 expression in GBM compared with LGG. In GBM cell lines, RFX7 expression was also significantly lower than in the normal astrocytic cell line (HA1800) (Figure 1D,E). Immunofluorescence showed lower RFX7 expression in GBM tissue than matched adjacent normal tissue (Figure 1F). Immunohistochemistry (IHC) revealed decreasing RFX7 immunoreactivity with increasing glioma grade, localized predominantly in nuclei (Figure 1G,H). Kaplan‐Meier analysis indicated that patients with higher RFX7 expression had longer overall survival (Figure 1I).

Given that transcriptional repression in cancer always arises from epigenetic mechanisms, we examined the methylation status of the *RFX7* promoter. Bisulfite pyrosequencing of the CpG sites within the multiple promoter regions (*RFX7*‐1s, *RFX7*‐3s, and *RFX7*‐4s) revealed significantly higher methylation levels in GBM compared with LGG (Figure 1J,K). These data indicated an association between promoter hypermethylation at specific CpG sites and lower *RFX7* expression in GBM. To validate the inhibitory effect of promoter methylation on *RFX7* expression, we treated GBM cell lines with the demethylating agent Azacitidine (AZA) (10 µmol/L, 48 h) [29, 34]. Following azacitidine treatment, both mRNA and protein expression of RFX7 were significantly upregulated (Figure 1L,M), indicating effective reversal of its transcriptional silencing, which directly confirms that *RFX7* expression is regulated by promoter methylation. Taken together, reduced *RFX7* expression in GBM is linked to aberrant promoter hypermethylation and is associated with poorer prognosis in glioma patients.

### 2.2 RFX7 Overexpression Inhibits GBM Cell Proliferation, Invasion, and Promotes Apoptosis

**FIGURE 2:**
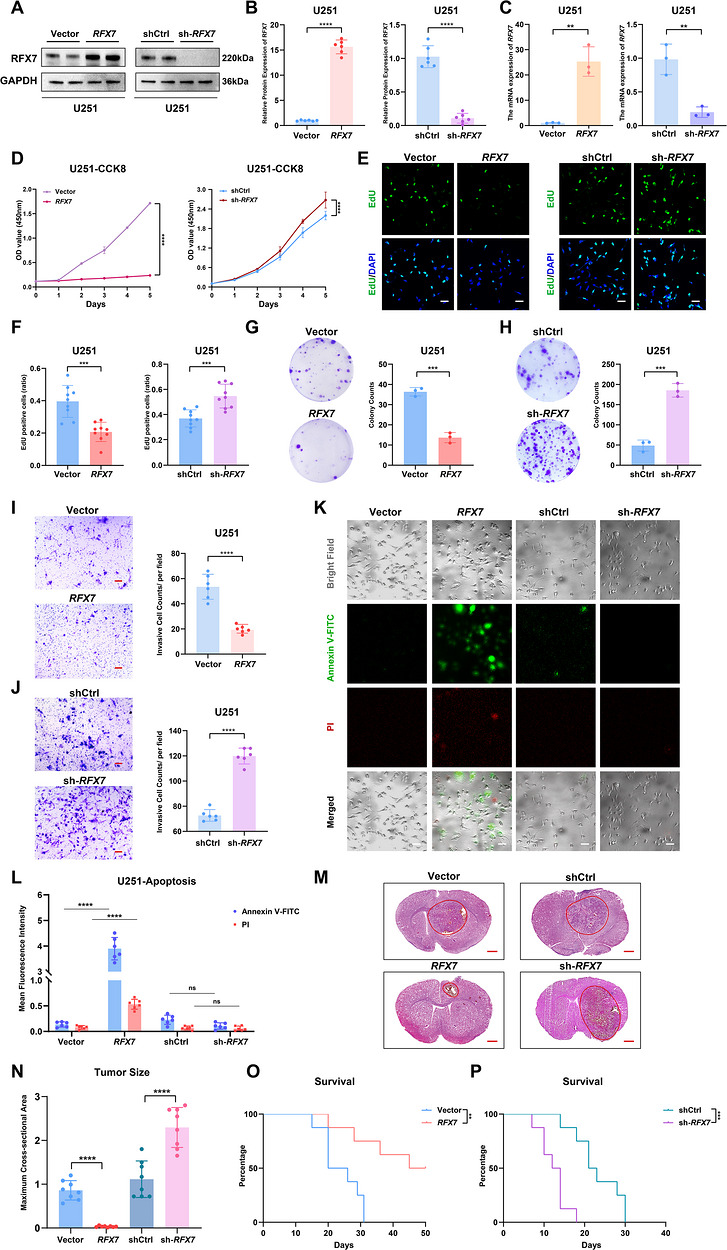
RFX7 Modulates the Malignant Phenotypes of U251 cells in vitro and in vivo. (A, B) Efficacy of RFX7 overexpression (A) and knockdown (B) in U251 cells validated by WB analysis (Student's *t*‐test; n = 6 per group). (C) The mRNA expression of *RFX7* in U251 cells after virus transfection (Student's *t*‐test; n = 3 per group). (D) Alterations in the proliferation capacity of U251 cells after interference with RFX7 expression in the CCK8 assay (Two‐way repeated measures ANOVA; 3 repeats at each time point). (E, F) Alterations in the DNA synthesis and proliferation capacity of U251 cells after interference with RFX7 expression in the EdU assay (Scale Bar = 20 µm; Student's *t*‐test; n = 8 per group). (G, H) Alterations in the clonogenic potential of U251 cells following RFX7 expression interference in the colony formation assay (Student's *t*‐test; n = 3 per group). (I, J) Alterations in the invasive capacity of U251 cells following RFX7 expression interference in the Transwell assay (Scale Bar = 20 µm; Student's *t*‐test; n = 6 per group). (K, L) Annexin V‐FITC/PI staining assay to assess the capacity of apoptosis in U251 cells (Scale Bar = 20 µm; Student's *t*‐test; n = 6 per group). (M, N) The tumor size analysis for mice in RFX7 overexpression (M) or knockdown (N) groups (Scale Bar = 1 mm; Student's *t*‐test; n = 8 per group). (O, P) Kaplan–Meier survival curves of mouse survival in RFX7 overexpression (O) or knockdown (P) groups (Log‐rank test; n = 8 per group). All values are shown as the mean ± SD. ^**^ *p* < 0.01, ^***^ *p* < 0.001, ^****^ *p* < 0.0001, ns: not significant.

Given the observed downregulation of RFX7 in GBM and its association with poor prognosis, we next investigated whether variation in RFX7 expression influences the malignant phenotypes of GBM cells. U251 and T98G cells were transfected with control vector (Vector) or RFX7‐overexpressing (*RFX7*) adenovirus, while knockdown was achieved using shRNA targeting *RFX7* (sh‐*RFX7*) compared with control shRNA (sh‐Ctrl). Protein and mRNA changes were confirmed by Western blot and qPCR in both cell lines (Figure 2A–C and Figure S2A–C).

Cell proliferation measured by CCK8 and EdU assays was significantly lower in RFX7‐overexpressing cells and higher following knockdown (Figure 2D–F and Figure S2D–F). Colony formation assay showed reduced clonogenic growth with RFX7 overexpression and increased colony numbers following RFX7 knockdown (Figure 2G,H and Figure S2G, H). Transwell assay demonstrated lower invasion capacity in RFX7‐overexpressing cells and higher invasion after knockdown (Figure 2I,J and Figure S2I, J). Annexin V‐FITC/PI apoptosis assay indicated that RFX7 overexpression was associated with an increased proportion of cells in the apoptotic phase compared with vector control, while no significant difference was observed between knockdown and control groups (Figure 2K,L and Figure S2K, L). In an intracranial xenograft model using U251 cells with BALB/c nude mice (n = 8 per group), RFX7 overexpression corresponded to smaller tumor volume and longer survival relative to vector control, whereas knockdown produced larger tumor volume and shorter survival (Figure 2M–P). Together, these data indicate that RFX7 exhibits inhibitory effects on GBM cell proliferation and invasion, and that its reduced expression may contribute to the aggressive behavior observed in GBM.

### 2.3 RFX7 Modulates the PI3K/AKT Pathway by Transcriptional Regulation of PIK3IP1

**FIGURE 3:**
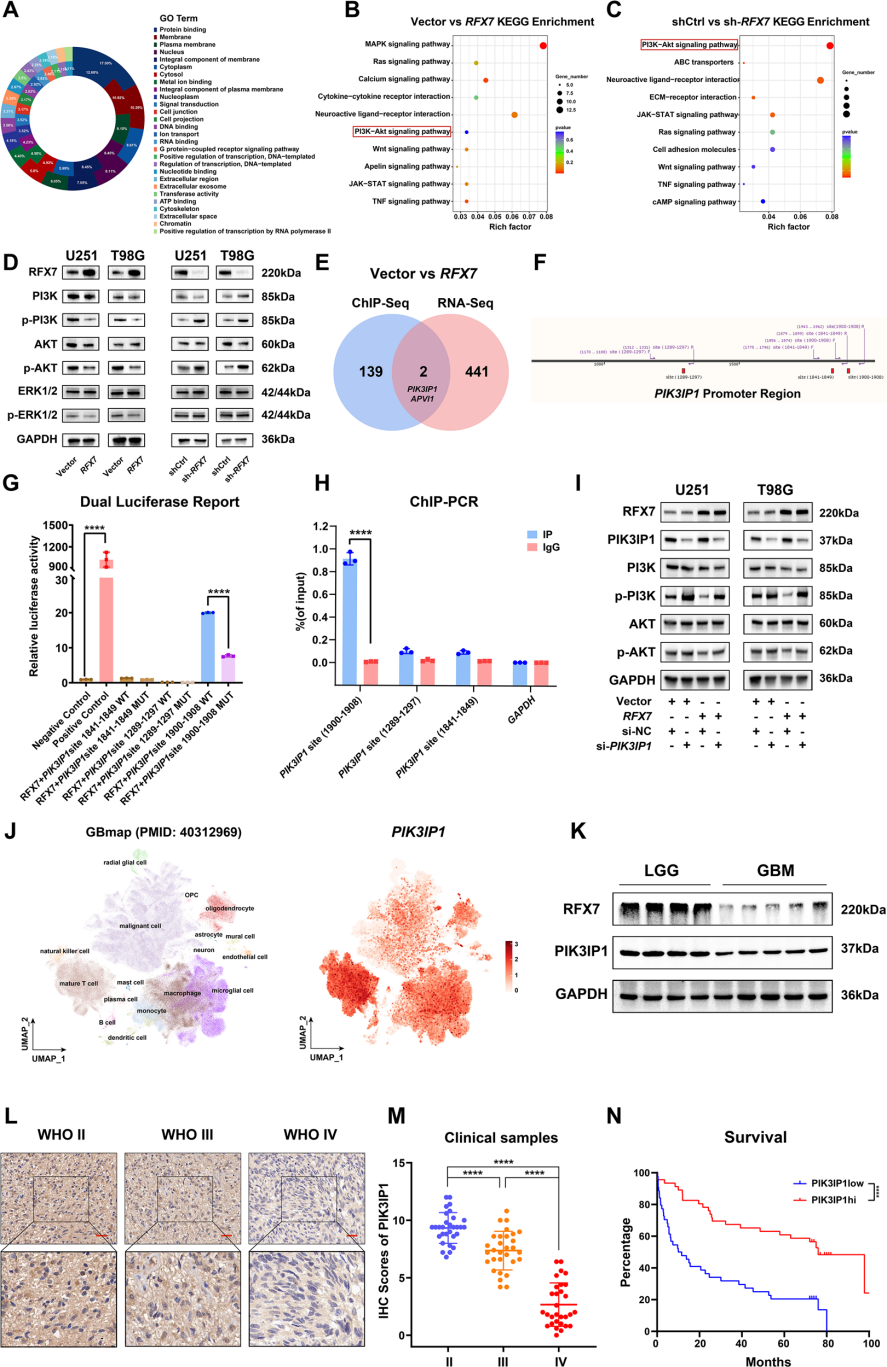
RFX7 Modulates the PI3K/AKT Pathway by Transcriptional Regulation of PIK3IP1. (A) GO enrichment analysis of DEGs from transcriptome sequencing data. (B, C) KEGG enrichment analysis revealed that both RFX7 overexpression (B) and knockdown (C) influenced the downstream PI3K/AKT signaling pathway. (D) WB analysis of downstream pathways in U251 and T98G cells with RFX7 interference. (E) Venn diagram of the overlapping genes from RNA‐seq and ChIP‐seq in Vector vs *RFX7* groups of U251 cells. (F) Schematic representation of three predicted RFX7‐binding sites within the *PIK3IP1* promoter region. (G) Dual‐luciferase reporter assay of the *PIK3IP1* promoter activity with promoter site mutations (Student's *t*‐test; n = 3 per group). (H) ChIP‐PCR assay of the enrichment of RFX7 at the binding sites of the *PIK3IP1* promoter with exogenous RFX7 expression (Student's *t*‐test; n = 3 per group). (I) WB analysis of p‐PI3K/p‐AKT in U251 and T98G cells with both RFX7 and PIK3IP1 interference. (J) UMAP visualization of the *PIK3IP1* expression in GBmap single‐cell atlas. (K) WB analysis of PIK3IP1 expression in LGG and GBM tissues. (L, M) IHC staining (L) and analysis (M) of the expression of PIK3IP1 in gliomas with different grades (Scale Bar = 50 µm, Kruskal–Wallis test; n = 30 per group; mean value of IHC scores: II: 9.33; III: 7.36; IV: 2.66). (N) Kaplan–Meier survival curves of the prognosis of glioma patients with different PIK3IP1 expression (Log‐rank test). All values are shown as the mean ± SD. *p* < 0.0001.

Based on the inhibitory effects of RFX7 on GBM cell proliferation and invasion, we next explored the downstream molecular mechanisms through which RFX7 may exert these functions, with a focus on signaling pathways linked to metabolic regulation and immune modulation. Transcriptome sequencing was performed on U251 cells from the Vector & *RFX7* groups and the shCtrl & sh‐*RFX7* groups. GO enrichment analysis of DEGs revealed that RFX7 modulated downstream signaling pathways, particularly in DNA transcription and cellular proliferation (Figure 3A). Among the KEGG enrichment analysis results of DEGs, we observed that both RFX7 overexpression and knockdown influenced the downstream PI3K/AKT signaling pathway (Figure 3B,C). In U251 and T98G cell lines, RFX7 overexpression decreased phosphorylated PI3K (p‐PI3K) and phosphorylated AKT (p‐AKT), while its knockdown produced the opposite changes. Besides, we examined the key proteins in the MAPK pathway that were most significantly affected by RFX7 overexpression. The expression of phosphorylated ERK1/2 was decreased in RFX7‐overexpressing cell lines, whereas it was unaffected in RFX7‐knockdown cell lines (Figure 3D). These results indicate that the effect of RFX7 on the phenotype of GBM cells is preferentially mediated through the PI3K/AKT pathway.

To further investigate how RFX7 regulates the PI3K/AKT pathway, we took the intersection of 141 potential RFX7 target genes from ChIP‐seq analysis and 443 up‐regulated DEGs from RNA‐seq analysis following RFX7 overexpression. Cross‐analysis of the above sequencing results detected that the *PIK3IP1* and *AVPI1* genes were present in both datasets (Figure 3E). In the public glioma patients’ datasets, *RFX7* exhibited a stronger Pearson correlation with *PIK3IP1* than with *AVPI1* (Figure S3A, B). Given that PIK3IP1 is known to inhibit PI3K activity and thereby affect downstream AKT signaling, a pathway connected to glycolytic metabolism and immune regulation, we hypothesized that RFX7 modulates PI3K/AKT signaling via PIK3IP1 [28, 30, 31]. Dual luciferase reporter assays using mutated *PIK3IP1* promoters revealed that mutations in the −1900 to −1908 bp region abolished changes in promoter activity linked to RFX7 overexpression, whereas mutations at other predicted sites had no effect (Figure 3F,G). ChIP‐qPCR confirmed RFX7 binding at the −1900 to −1908 bp site, with no enrichment at the other tested loci (Figure 3H). WB and qPCR results showed that *RFX7* overexpression increased and knockdown decreased PIK3IP1 expression in the cell lines studied (Figure S3C–E). In *RFX7*‐overexpressing cells, silencing *PIK3IP1* expression with siRNA restored PI3K/AKT phosphorylation toward the levels observed in the si‐NC group (Figure 3I).

In the public databases, compared with LGG, *PIK3IP1* expression was downregulated in GBM, and higher *PIK3IP1* expression was associated with a favorable prognosis (Figure S3F, G). We analyzed the cellular expression of *PIK3IP1* in the GBmap and CNP0003766) single‐cell atlases. Results showed that *PIK3IP1* was broadly expressed across multiple cell lineages, with high enrichment in malignant/GBM cells as well as the immune population (Figure , Figure S3H). We further explored *PIK3IP1* expression patterns across GBmap‐defined cell populations, stratified by TCGA expression levels. At the malignant cell state levels, the low *PIK3IP1* expression group was significantly enriched in the MES‐like, AC‐like, and OPC‐like states (Figure S3I). The significant enrichment in the MES‐like state is consistent with the *RFX7*‐low expression group. Further analysis of nine GBmap subtypes revealed the marked differences across specific subpopulations (Figure S3J).

In glioma samples, PIK3IP1 expression also decreased with reduced RFX7 expression (Figure 3K and Figure S3K). IHC staining also revealed decreasing PIK3IP1 expression with increasing glioma grade (Figure 3L,M). Matched tissue analysis demonstrated a significant association between RFX7 and PIK3IP1 levels (Figure S3L, M), and patient data revealed longer survival in cases with higher PIK3IP1 expression (Figure 3N). These findings indicate that RFX7 directly activates PIK3IP1 transcription, thereby modulating PI3K/AKT signaling in GBM.

### 2.4 RFX7‐PIK3IP1 Axis Regulates GBM Cell Proliferation, Invasion, and Tumorigenicity

**FIGURE 4:**
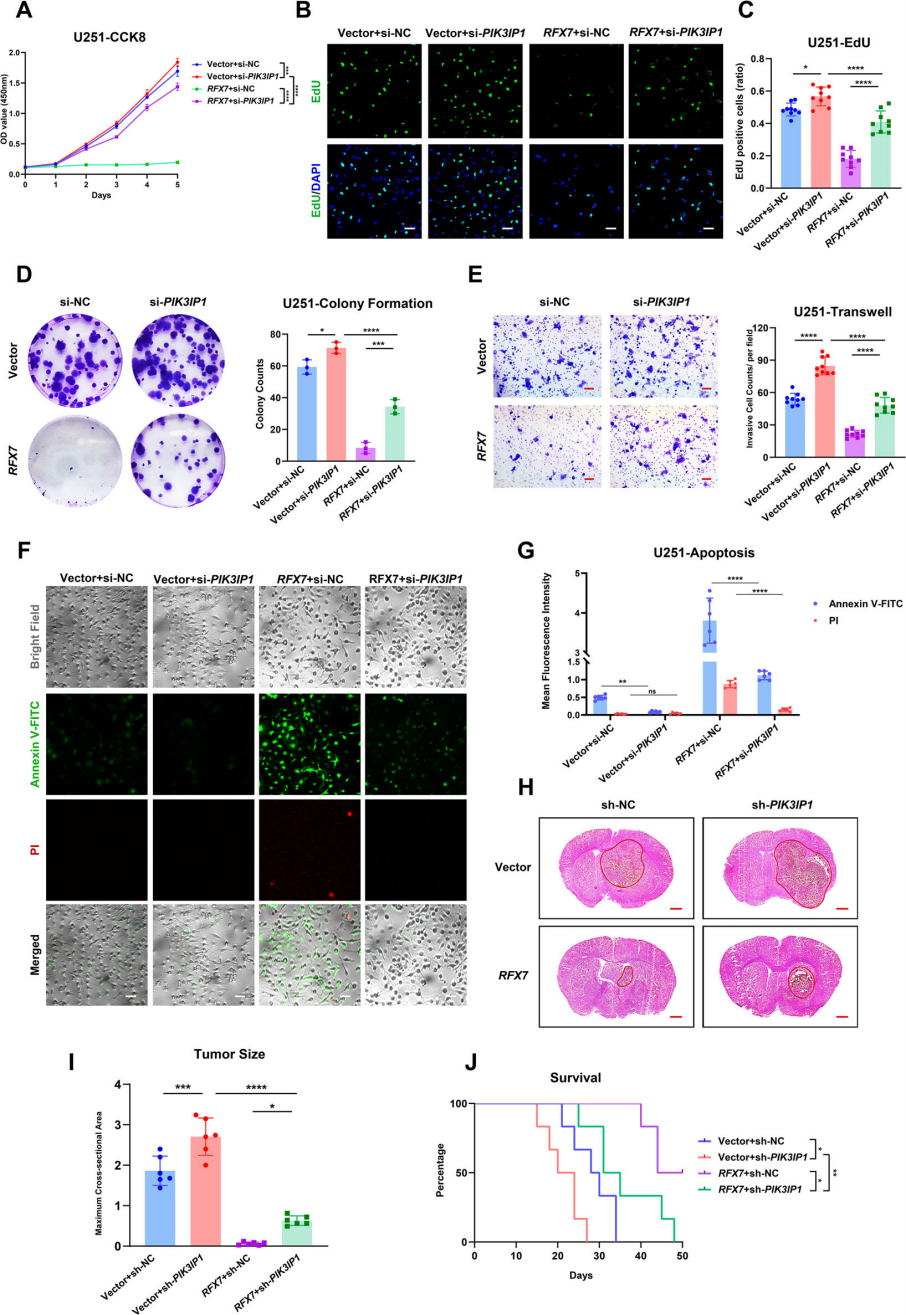
RFX7‐PIK3IP1 Axis Regulates the Malignant Phenotypes of U251 cells. (A) Cell proliferation assessed by CCK‐8 assay in U251 cells after RFX7 and PIK3IP1 intervention (Two‐way repeated measures ANOVA; 3 repeats at each time point). (B, C) Evaluation of DNA synthesis and proliferation by EdU assay in U251 cells following modulation of RFX7 and PIK3IP1 expression (Scale Bar = 20 µm; Two‐way ANOVA; n = 9 per group). (D) Clonogenic potential measured by colony formation assay in U251 cells following modulation of RFX7 and PIK3IP1 expression (Two‐way ANOVA; n = 3 per group). (E) Cell invasion capacity determined by Transwell assay in U251 cells following modulation of RFX7 and PIK3IP1 expression (Scale Bar = 20 µm; Two‐way ANOVA; n = 9 per group). (F, G) Apoptosis status analyzed by Annexin V‐FITC/PI staining in U251 cells following modulation of RFX7 and PIK3IP1 expression (Scale Bar = 20 µm; Two‐way ANOVA; n = 6 per group). (H, I) The tumor size analysis of orthotopic xenograft tumors following modulation of RFX7 and PIK3IP1 expression (Scale Bar = 1 mm; Two‐way ANOVA; n = 6 per group). (J) Kaplan–Meier survival curves of mice bearing tumors from the indicated groups (Log‐rank test; n = 6 per group). All values are shown as the mean ± SD. ^*^ *p* < 0.05, ^**^ *p* < 0.01, ^***^ *p* < 0.001, ^****^ *p* < 0.0001, ns: not significant.

Having identified PIK3IP1 as a direct transcriptional target of RFX7, we next assessed whether the RFX7‐PIK3IP1 axis is responsible for the effects of RFX7 on GBM cell malignant phenotypes. In CCK8 and EdU assays, compared to control siRNA (si‐NC), knockdown of *PIK3IP1* with siRNA (si‐*PIK3IP1*) partially increased cell viability in U251 and T98G cells transfected with Vector. Specifically, co‐transfection of *RFX7* and si‐*PIK3IP1* reversed the suppression of cell viability compared to co‐transfection of *RFX7* and si‐NC (Figure 4A–C and Figure S4A–C). Similarly, the colony‐formation assay showed that GBM cells co‐transfected with Vector and si‐*PIK3IP1* exhibited higher colony counts compared to cells co‐transfected with Vector and si‐NC. The colony counts were elevated in cells co‐transfected with RFX7 and si‐*PIK3IP1*, compared to those co‐transfected with *RFX7* and si‐NC (Figure 4D and Figure S4D). The Transwell assay results indicated that si‐*PIK3IP1* enhanced the invasive capacity of GBM cells. The invasive capacity was decreased following co‐transfection with *RFX7* and si‐NC, which was partially restored by co‐transfection with *RFX7* and si‐*PIK3IP1* (Figure 4E and Figure S4E). Annexin V‐FITC/PI immunofluorescence assay results showed that GBM cells co‐transfected with Vector and si‐NC or with Vector and si‐*PIK3IP1* displayed no significant apoptosis. Cells co‐transfected with *RFX7* and si‐*PIK3IP1* showed lower apoptotic status than those co‐transfected with *RFX7* and si‐NC, indicating that si‐*PIK3IP1* reduced the apoptotic effect of RFX7 (Figure 4F,G and Figure S4F, G). Collectively, co‐transfection with si‐*PIK3IP1* significantly abolished the inhibitory effects of RFX7 on GBM cell malignant phenotypes.

To further validate the regulatory role of the RFX7‐PIK3IP1 axis in vivo, U251 cells stably expressing a shRNA targeting *PIK3IP1* (sh‐*PIK3IP1*) or a nontargeting control (sh‐NC) were established via lentiviral transduction (Figure S4H, I). These cells, along with the indicated experimental groups, were intracranially injected into BALB/c nude mice (n = 6 per group). Mice implanted with cells co‐expressing *RFX7* and sh‐*PIK3IP1* developed significantly larger tumors and exhibited shorter survival compared with those receiving cells co‐expressing *RFX7* and the control sh‐NC. Conversely, the combination of *RFX7* and sh‐*PIK3IP1* resulted in smaller tumors and longer survival relative to mice injected with control vector cells expressing sh‐*PIK3IP1* alone (Figure 4H–J). These functional assays indicate that the loss of PIK3IP1 attenuates the inhibitory effects of RFX7 on GBM cell proliferation, invasion, and apoptosis, supporting a role for the RFX7‐PIK3IP1 axis in modulating tumor growth capacity.

### 2.5 Higher PIK3IP1 Expression Correlates with a Less Immunosuppressive TME Profile

**FIGURE 5:**
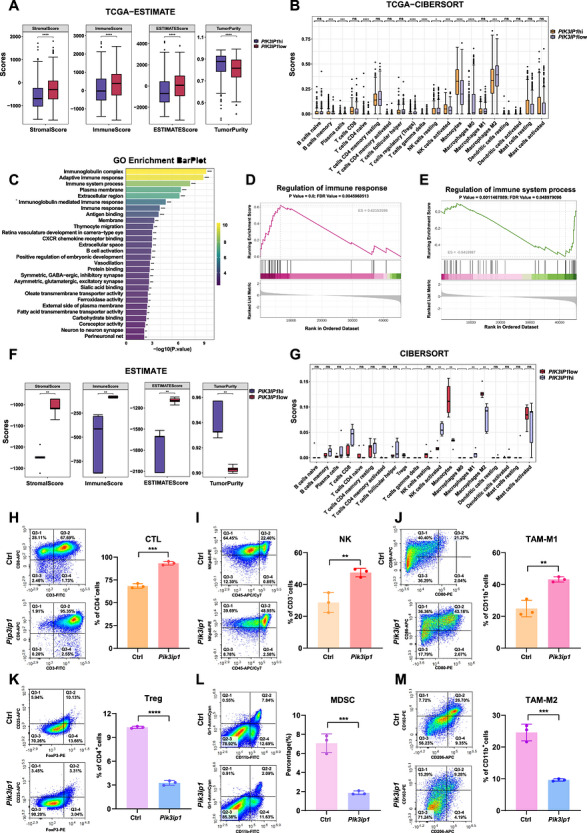
Higher PIK3IP1 Expression Correlates with a Less Immunosuppressive TME Profile. (A, B) ESTIMATE (A) and CIBERSORT (B) analyses of immune infiltration and tumor purity at different *PIK3IP1* expression levels in the TCGA database (Mann–Whitney U test). (C) GO enrichment analysis of DEGs with high and low *PIK3IP1* expression GBM samples. (D, E) GSEA enrichment analysis plots of immune process (D) and immune system process (E). (F) ESTIMATE analysis of the stromal content and tumor purity with different *PIK3IP1* expression in GBM samples (Mann–Whitney U test). (G) CIBERSORT analysis of immune infiltration with different *PIK3IP1* expression in GBM samples (Mann–Whitney U test). (H–J) Flow cytometry analyses of cytotoxic immune cells: CTLs (H), NKs (I) and TAM‐M1s (J), in GBM tissues from Ctrl and *Pik3ip1* group mice (Student's *t*‐test, n = 3 per group). (K–M) Flow cytometry analyses of immunosuppressive cells: Tregs (K), MDSCs (L) and TAM‐M2s (M), in GBM tissues from Ctrl and *Pik3ip1* group mice (Student's *t*‐test, n = 3 per group). All values are shown as the mean ± SD. ^*^ *p* < 0.05, ^**^ *p* < 0.01, ^***^ *p* < 0.001, ^****^ *p* < 0.0001, ns: not significant.

Given the association of the RFX7‐PIK3IP1 axis with GBM cell proliferation and invasion, we next examined whether variation in PIK3IP1 expression is linked to differences in tumor immune microenvironment composition in GBM. Analysis of the public databases using ESTIMATE, CIBERSORT, and CIBERSORTx with GBmap cell type revealed that lower expression was associated with higher stromal and immune scores, reduced tumor purity, and increased infiltration of multiple immune/stromal cell populations (Figure 5A,B and Figure S5A, B). Transcriptomic sequencing was performed on tumor samples from 10 GBM patients collected intraoperatively. GO enrichment analysis revealed that DEGs between subgroups with different *PIK3IP1* expression levels were significantly enriched in immune‐related pathways (Figure 5C and Figure S5C). Reactome pathway analysis further indicated that the DEGs also showed significant enrichment in immune‐related molecular functions, including immune cell surface antigen recognition and immunoglobulin‐mediated immune responses (Figure S5D). GSEA results revealed that compared to the *PIK3IP*1high (*PIK3IP1*hi) group, the PIK3IP1low group showed significant genes enrichment associated with the regulation of immune response and immune system process (Figure 5D,E), especially in the regulation of proliferation and activation in different immune cells (Figure S5E–H). Compared to the *PIK3IP1*low group, the *PIK3IP1*hi group was associated with reduced immune and stromal scores and elevated tumor purity (Figure 5F). Immune infiltration patterns estimated by CIBERSORT analysis revealed that compared with the *PIK3IP1*low group, the *PIK3IP1hi* group exhibited higher scores for antitumor immune cells (CD8^+^ T cells, activated NK cells, M1 macrophages), and lower scores for immunosuppressive cell types (regulatory T cells, monocytes, M2 macrophages) (Figure 5G). T‐cell State Score (TCSS) analysis indicated the downregulation of regulatory T cell‐associated signaling and the upregulation of cytotoxic T cell‐associated signaling in the *PIK3IP1hi* group, consistent with CIBERSORT findings (Figure S5I). These analyses indicate that high PIK3IP1 expression promotes an antitumor microenvironment with amplified cytotoxic T cell function and a reduction in immunosuppressive elements.

To validate the bioinformatics analysis results, we established an orthotopic tumor mouse model using GL261 glioma cells that were stably transfected with lentivirus. The cells expressed either a *Pik3ip1* overexpression construct (*Pik3ip1* group) or an empty vector control (Ctrl group), and tumor growth was monitored for 28 days (Figure S5J–L). Flow cytometry analysis of dissociated GBM tissues revealed that compared to the Ctrl group, the *Pik3ip1* group exhibited significantly increased infiltration of cytotoxic immune cells, including cytotoxic T lymphocytes (CTLs, CD3^+^CD4^−^CD8^+^), natural killer cells (NKs, CD3^−^CD45^+^NKp46^+^), and M1‐type tumor‐associated macrophages (TAM‐M1s, CD11b^+^CD80^+^CD86^+^) (Figure 5H–J). Conversely, immunosuppressive cells, including regulatory T cells (Tregs, CD4^+^CD25^+^FoxP3^+^), myeloid‐derived suppressor cells (MDSCs, CD11b^+^Gr1^+^), and M2‐type tumor‐associated macrophages (TAM‐M2s, CD11b^+^CD106^+^CD206^+^), were markedly reduced (Figure 5K–M).

These results suggest that higher PIK3IP1 expression is associated with a TME enriched in cytotoxic immune cells and lower levels of immunosuppressive cell types, linking the RFX7‐PIK3IP1 axis to immune composition changes in GBM.

### 2.6 PIK3IP1 Silencing Regulates Lactate Accumulation and Activates Histone H4K12 Lactylation in GBM

**FIGURE 6:**
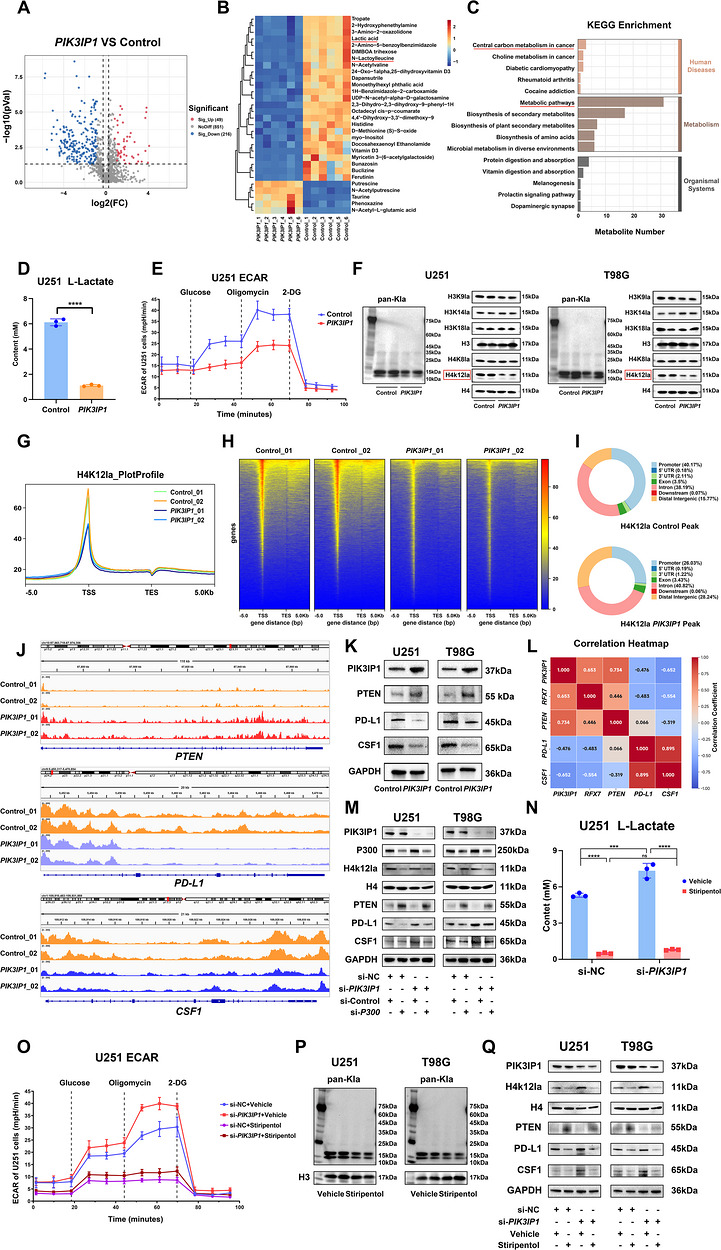
PIK3IP1 Silencing Regulates Lactate Accumulation and Activates Histone H4K12 Lactylation in GBM. (A) Volcano plot of the differentially expressed metabolites from the metabolomic analysis. (B) Heatmap of the top 30 most significantly altered metabolites from the metabolomic analysis. (C) KEGG enrichment analysis of the differentially expressed metabolites. (D) Intracellular L‐lactate concentration measured in the Control and *PIK3IP1* groups of U251 cells (Student's *t*‐test, n = 3 per group). (E) Extracellular acidification rate measured in the Control and *PIK3IP1* groups of U251 cells. (F) WB assessment of histone lactylation levels in the Control and *PIK3IP1* groups of U251 and T98G cells. (G) Profile plot of the distribution of H4K12 lactylation signals across gene regions. (H) Heatmap of enrichment signals around genes within the TSS regions from CUT&Tag sequencing. (I) Functional annotation of peaks enrichment in the Control and *PIK3IP1* groups. (J) IGV visualization of sequencing peaks for key genes (*PTEN*, *PD‐L1* and *CSF1*) from CUT&Tag sequencing. (K) WB analysis of PTEN, PD‐L1, and CSF1 protein expression in U251 and T98G cells following PIK3IP1 modulation. (L) Correlation analysis of *PIK3IP1*, *RFX7*, *PTEN*, *PD‐L1* and *CSF1* in the transcriptome data of GBM samples (Pearson correlation analysis). (M) WB analysis of downstream targets expression in U251 and T98G cells following PIK3IP1 and P300 interference. (N) Intracellular L‐lactate concentration of U251 cells following PIK3IP1 interference and Stiripentol treatment (Two‐way ANOVA; n = 3 per group). (O) Extracellular acidification rate of U251 cells following PIK3IP1 interference and Stiripentol treatment. (P) WB analysis of pan‐lysine lactylation in U251 and T98G cells after Stiripentol treatment. (Q) WB analysis of H4K12la, PTEN, PD‐L1, and CSF1 protein expression in U251 and T98G cells following PIK3IP1 interference and Stiripentol treatment. All values are shown as the mean ± SD. ^***^ *p* < 0.001, ^****^ *p* < 0.0001, ns: not significant.

Given that the RFX7‐PIK3IP1 axis is associated with changes in immune cell composition and is linked to PI3K/AKT signaling, which can influence glycolytic metabolism, we next investigated whether PIK3IP1 affects lactate production and lactate‐related epigenetic modifications. Nontargeted metabolomic profiling by LC‐MS was performed on U251 cells overexpressing PIK3IP1 (*PIK3IP1*) and control cells (Control). Quantitative analysis identified 49 metabolites at higher levels and 216 metabolites at lower levels in the *PIK3IP1* group compared with the control group (Figure 6A). Heatmap visualization of the top 30 altered metabolites showed decreased levels of lactic acid and N‐lactoylleucine in the *PIK3IP1* group (Figure 6B and Figure S6A,B). KEGG enrichment analysis indicated that these altered metabolites were significantly enriched in pathways related to carbon metabolism in cancer cells (Figure 6C and Figure S6C, D). The experimental results showed that intracellular lactate production and extracellular acidification rate (ECAR) were reduced when PIK3IP1 was overexpressed in U251 and T98G cells (Figure 6D,E and Figure S6E, F).

Enhanced lactate production can induce histone lactylation, a novel post‐translational modification that alters chromatin structure and gene transcription, influencing tumor progression [13, 35]. WB analysis showed lower global histone lactylation (pan‐Kla) in *PIK3IP1* groups compared with Control groups. Site‐specific assays (including H3K9, H3K14, H3K18, H4K8, and H4K12) identified the largest decrease at H4K12 lactylation (H4K12la) in *PIK3IP1* groups (Figure 6F). CUT&Tag profiling using H4K12la antibody showed lower enrichment pattern near the transcription start site (TSS) in the *PIK3IP1* group than in the Control group (Figure 6G). The heatmap of the enrichment signals in the TSS region was weaker in the *PIK3IP1* group compared to the Control group (Figure 6H). Functional annotation of the peaks revealed that the Control group exhibited a higher proportion of peaks in the promoter region (40.1%), whereas the *PIK3IP1* group showed a relatively lower enrichment in this region (26.03%) (Figure 6I). KEGG analysis of the upregulated peaks in the *PIK3IP1* group showed primary enrichment in immune‐related pathways, such as the T cell receptor signaling pathway and the PD‐L1 expression/PD‐1 checkpoint pathway in cancer (Figure S6G).

Furthermore, the peaks of key genes in the differentially enriched pathways were visualized through Integrative Genomics Viewer (IGV). The results showed that Phosphatase and tensin homolog (*PTEN*) expression levels were significantly elevated in the *PIK3IP1* group, which could effectively inhibit the PI3K/AKT signaling pathway [36]. Concurrently, the expression levels of *CD274* (*PD‐L1*) and Colony‐Stimulating Factor 1 (*CSF1*) were markedly reduced in the *PIK3IP1* group (Figure 6J). Validation experiments in U251 and T98G cells further supported the above findings (Figure 6K). Correlation analysis of the above genes in our GBM transcriptome data and TCGA database demonstrated that the expression of *RFX7* and *PIK3IP1* was positively correlated with the expression of *PTEN*, while inversely correlated with the expression of *PD‐L1* and *CSF1* (Figure 6L and Figure S6H).

To further elucidate how PIK3IP1 regulates H4K12 lactylation, we suppressed the histone lactylation “writer” P300 via si‐*P300* [37]. Knockdown of *PIK3IP1* significantly elevated H4K12la in U251 and T98G cells, which was markedly reversed by additional si‐*P300* treatment, confirming P300 as a key mediator of PIK3IP1‐regulated H4K12la. Regarding downstream targets, si‐*P300* upregulated PTEN expression, which was suppressed by si‐*PIK3IP1*, while inhibiting PD‐L1 and CSF1 expression, which were restored by si‐*PIK3IP1* (Figure 6M). Collectively, PIK3IP1 modulates PTEN, PD‐L1, and CSF1 transcription via P300‐mediated H4K12la modification.

Stiripentol is an anticonvulsant drug to inhibit lactate production in preclinical cancer studies [38, 39]. We treated U251 and T98G cell lines with 500 µm Stiripentol (Stiripentol group), using an equal volume of DMSO as the control (Vehicle group). Compared to the Vehicle group, Stiripentol significantly attenuated the increase of intracellular L‐lactate and ECAR induced by si‐*PIK3IP1* (Figure 6N,O and Figure S6I, J). Furthermore, Stiripentol treatment markedly reduced the global pan‑Kla level (Figure 6P). The administration of Stiripentol decreased the elevated expression of H4K12la, PD‐L1, and CSF1 induced by si‐*PIK3IP1*, while restoring the expression of PTEN, which was downregulated by si‐*PIK3IP1* (Figure 6Q). Dual downregulation of PD‐L1 and CSF1 expression is crucial for reversing immune suppression and inhibiting tumor immune escape and progression [40, 41].

These findings indicate that reduced PIK3IP1 expression is associated with increased lactate production and H4K12 lactylation, accompanied by higher PD‐L1 and CSF1 expression. Both PD‐L1 and CSF1 can promote an immunosuppressive TME and reduce the effectiveness of immune checkpoint blockade. Thus, metabolic changes linked to PIK3IP1 deficiency may have functional relevance for immunotherapy resistance in GBM.

### 2.7 Stiripentol Administration Reverses Tumor Malignancy and Alters Immune Cell Composition in GBM

**FIGURE 7:**
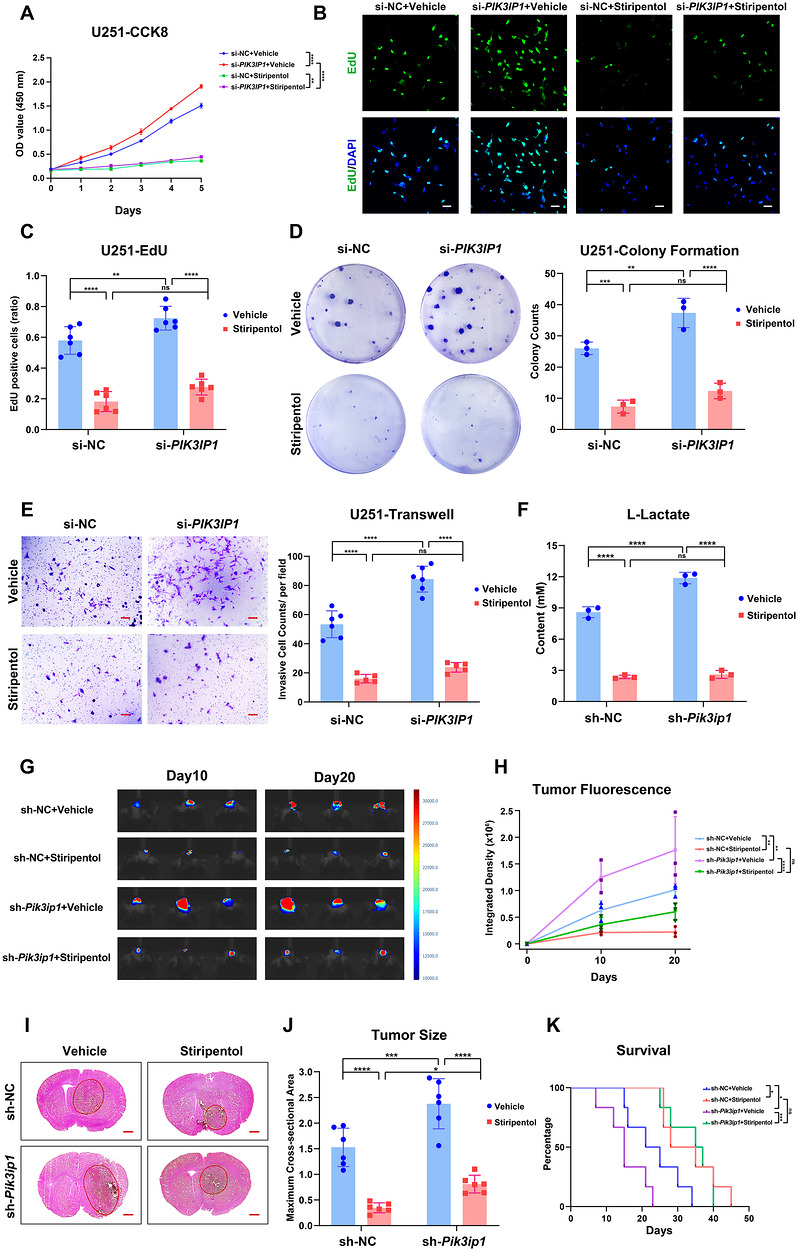
Stiripentol Reverses the Tumor Malignancy Induced by PIK3IP1 Silencing. (A) CCK‐8 assay for proliferation capacity of U251 cells following Stiripentol treatment after si‐NC/*PIK3IP1* interference (Two‐way repeated measures ANOVA; 3 repeats at each time point). (B, C) Evaluation of DNA synthesis and proliferation by EdU assay in U251 cells following si‐NC/*PIK3IP1* interference and Stiripentol administration (Scale Bar = 20 µm; Two‐way ANOVA; n = 6 per group). (D) Clonogenic potential measured by colony formation assay in U251 cells following si‐NC/*PIK3IP1* interference and Stiripentol administration (Two‐way ANOVA; n = 3 per group). (E) Cell invasion capacity determined by Transwell assay in U251 cells following si‐NC/*PIK3IP1* interference and Stiripentol administration (Scale Bar = 20 µm; Two‐way ANOVA; n = 6 per group). (F) L‐lactate concentration at GBM sites in sh‐NC/*Pik3ip1* mice following Stiripentol treatment (Two‐way ANOVA; n = 3 per group). (G, H) In vivo fluorescence imaging of intracranial GBM growth in sh‐NC/*Pik3ip1* mice following Stiripentol treatment (Two‐way repeated measures ANOVA; 3 repeats at each time point). (I, J) The tumor size analysis of orthotopic xenograft tumors in sh‐NC/*Pik3ip1* mice following Stiripentol treatment (Scale Bar = 1 mm; Two‐way ANOVA; n = 6 per group). (K) Kaplan–Meier survival curves of sh‐NC/*Pik3ip1* mice bearing tumors following Stiripentol treatment (Log‐rank test; n = 6 per group). All values are shown as the mean ± SD. *p < 0.05, **p < 0.01, ***p < 0.001, ****p < 0.0001, ns: not significant.

Since inhibition of lactate production in vitro reduced H4K12la and reversed the expression of the immune‐related genes, we next assessed whether pharmacological blockade of lactate production with Stiripentol could affect GBM progression and the tumor immune microenvironment. In CCK8, EdU, colony formation, and Transwell assays, Stiripentol treatment effectively counteracted the pro‐tumor effects induced by si‐*PIK3IP1*, and suppressed the proliferation and invasion of U251 and T98G cells compared with Vehicle control (Figure 7A–E and Figure S7A–G). To validate the antitumor efficacy of Stiripentol in vivo, GL261‐luc cells transfected with sh‐NC or sh‐*Pik3ip1* were transplanted into the mouse brain. The mice were then treated with either Stiripentol (200 mg kg^−1^day^−1^) or Vehicle for 28 days. L‐lactate detection in the tumor site showed that lactate level was significantly increased in the sh‐*Pik3ip1* group compared with the sh‐NC group, while Stiripentol treatment markedly reduced intratumoral lactate content (Figure 7F). The administration of Stiripentol also inhibited the intracranial tumor growth and prolonged survival in GBM‐bearing mice (Figure 7G–K).

**FIGURE 8:**
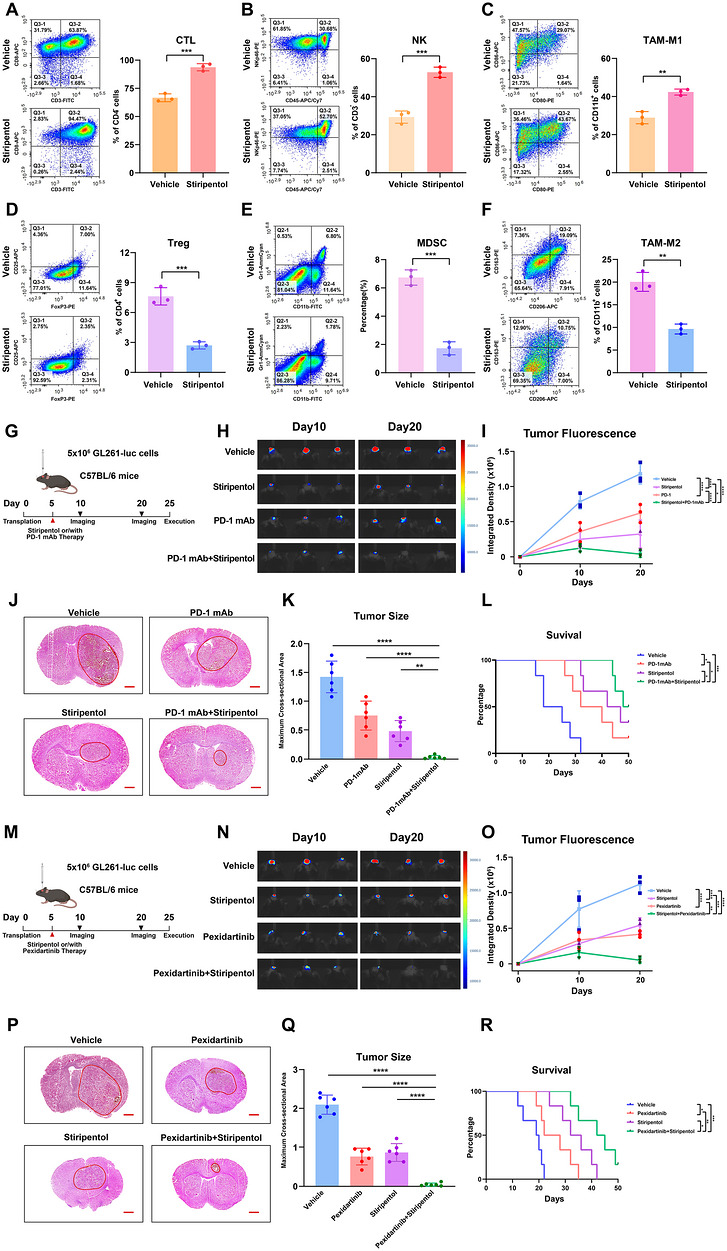
Stiripentol Reverses Immunosuppressive Microenvironment and Synergizes with immunotherapy in GBM. (A–C) Flow cytometry analyses of cytotoxic immune cells: CTLs (A), NKs (B) and TAM‐M1s (C), in GBM tissues from Vehicle and Stiripentol treated mice (Student's *t*‐test, n = 3 per group). (D–F) Flow cytometry analyses of immunosuppressive cells: Tregs (D), MDSCs (E) and TAM‐M2s (F), in GBM tissues from Vehicle‐ and Stiripentol‐ treated mice (Student's *t*‐test, n = 3 per group). (G) Schematic diagram of the administration of Stiripentol or/with PD‐1 mAb therapy. (H, I) In vivo fluorescence imaging of intracranial GBM growth in mice treated with Stiripentol or/with PD‐1 mAb (Two‐way repeated measures ANOVA; 3 repeats at each time point). (J, K) The tumor size analysis of orthotopic xenograft tumors from mice treated with Vehicle, PD‐1 mAb, Stiripentol, and PD‐1 mAb+Stiripentol (Scale Bar = 1 mm, One‐way ANOVA, n = 6 per group). (L) Kaplan–Meier survival curves of mice bearing tumors from the indicated groups (Log‐rank test, n = 6 per group). (M) Schematic diagram of the administration of Stiripentol or/with Pexidartinib therapy. (N, O) In vivo fluorescence imaging of intracranial GBM growth in mice treated with Stiripentol or/with Pexidartinib (Two‐way repeated measures ANOVA; 3 repeats at each time point). (P, Q) The tumor size analysis of orthotopic xenograft tumors from mice treated with Vehicle, Pexidartinib, Stiripentol, and Pexidartinib+Stiripentol (Scale Bar = 1 mm, One‐way ANOVA, n = 6 per group). (R) Kaplan–Meier survival curves of mice bearing tumors from the indicated groups (Log‐rank test, n = 6 per group). All values are shown as the mean ± SD. ^*^ *p* < 0.05, ^**^ *p* < 0.01, ^***^ *p* < 0.001, ^****^ *p* < 0.0001, ns: not significant.

Moreover, in the GL261‐luc orthotopic GBM model, Stiripentol treatment reshaped immune cell infiltration in TME. Flow cytometry analysis revealed that Stiripentol significantly increased the infiltration of antitumor immune cells, including CTLs, NKs, and TAM‐M1s (Figure 8A–C), while the infiltration of immunosuppressive cells such as Tregs, MDSCs, and TAM‐M2s were significantly decreased, compared with the Vehicle group (Figure 8D–F). Given the effects of Stiripentol on immune cell composition and PD‐L1/CSF1 expression, we evaluated combination therapy of Stiripentol with the FDA‐approved anti‐PD‐1 antibody (5 mg kg^−1^ day^−1^), as well as with the CSF1R inhibitor Pexidartinib (2 mg kg^−1^ day^−1^). Treatments began five days after tumor implantation and continued daily for 20 days. The combination therapy resulted in a significant reduction in tumor volume and longer survival time compared to anti‐PD‐1 antibody or Stiripentol monotherapy (Figure 8G–L). Pexidartinib exhibited obviously combinatorial efficacy with Stiripentol, similar to an anti‐PD‐1 antibody (Figure 8M–R). These findings suggest that Stiripentol, inhibiting lactate production, suppresses GBM growth and remodels the TME. Combining Stiripentol with immune checkpoint blockade or CSF1R inhibition yields superior antitumor efficacy compared to monotherapies.

## 3 Discussion

GBM remains a highly malignant brain tumor with poor responsiveness to conventional therapy and limited benefit from immune checkpoint inhibition [4, 42]. Although excessive glycolysis and lactate accumulation are known to promote an immunosuppressive microenvironment [10, 43, 44], the upstream regulatory mechanisms connecting lactate metabolism with immune dysfunction in GBM have not been clearly defined. In this study, integrated analyses of patient datasets and experimental models identified that epigenetic silencing of RFX7 corresponds with reduced expression of its transcriptional target PIK3IP1 and activation of PI3K/AKT signaling. These alterations are associated with increased lactate production and histone H4K12 lactylation (H4K12la), accompanied by upregulation of PD‐L1 and CSF1 and reduced cytotoxic immune activity, indicating that lactate‐driven epigenetic modification participates in immune suppression in GBM. Pharmacological inhibition of lactate production using Stiripentol partially reversed these molecular and immune changes and enhanced the efficacy of immune‐based combination therapy under experimental conditions.

Transcription factor RFX7 was initially recognized as a tumor suppressor frequently mutated in lymphoid neoplasms [25]. However, the function of RFX7 in GBM remains poorly characterized. Our study demonstrates that loss of RFX7 expression is a frequent event in GBM, and higher RFX7 expression in GBM patients correlates with a more favorable prognosis. The IDH mutation status is a key determinant in defining molecular subtypes and predicting prognosis in GBM [45]. IDH‐wildtype GBM represents the classic primary form, characterized by genomic instability, high invasiveness, and poor prognosis, whereas IDH‐mutant GBM typically evolves from LGG and follows a relatively indolent clinical course [46]. Our study found that low RFX7 expression in IDH‐wildtype patients correlates with unfavorable outcomes, while high RFX7 expression in IDH‐mutant patients is associated with better prognosis, suggesting that IDH status may be related to RFX7 expression and promoter methylation. It is known that core drivers of IDH‐wildtype GBM, such as EGFR amplification, persistently activate signaling pathways like PI3K/AKT, promoting uncontrolled proliferation and apoptosis resistance [47, 48, 49]. This study further indicates that loss of RFX7 expression attenuates its inhibitory effect on the PI3K/AKT pathway, thereby aligning mechanistically with the progression of IDH‐wildtype glioma, offering new insights into the role of RFX7 in this subtype.

Mechanistically, the tumor‐suppressive activity of RFX7 in glioma was mediated through direct transcriptional activation of PIK3IP1. PIK3IP1 is a transmembrane inhibitor of the PI3K/AKT pathway, serving as a critical adjustment point for immune and metabolic homeostasis [30, 50, 51]. In resting T cells, PIK3IP1 serves as a regulatory “brake” to prevent excessive activation by constraining PI3K/AKT signaling. Loss of PIK3IP1 expression has been associated with metabolic shifts from oxidative phosphorylation to aerobic glycolysis in autoimmune settings [52]. In GBM, our data reveal that downregulation of PIK3IP1, resulting from reduced RFX7 activity, lifts the inhibition of the PI3K/AKT pathway and fosters an immunosuppressive TME through enhanced aerobic glycolysis and lactate accumulation. Consequently, PIK3IP1 exhibits a context‐dependent role: its reduced expression in T cells promotes autoimmunity [52], whereas its loss in glioma cells supports tumor immune evasion. This duality underscores the potential of modulating PIK3IP1 expression to rebalance immune activation in both malignancy and autoimmune diseases. Moreover, downregulation of RFX7‐PIK3IP1 axis was linked to a more immunosuppressive TME, characterized by diminished cytotoxic cell infiltration and increased expression of immune checkpoint molecules. These findings indicate that the RFX7‐PIK3IP1 axis contributes not only to tumor suppression but also to the modulation of immune contexture within GBM, further supporting its relevance as a molecular determinant of therapeutic response.

Lactate has traditionally been regarded as a byproduct of glycolytic metabolism. However, recent studies have revealed that it serves not only as an energy metabolite but also as a crucial signaling molecule involved in tumor progression and immune regulation [11, 43, 44]. GBM, characterized by the Warburg effect, exhibits a strong preference for glycolysis even under aerobic conditions, resulting in the excessive production of lactate. Consequently, the lactate concentration in TME is significantly elevated compared to that in normal tissues. This metabolic reprogramming not only sustains the rapid proliferation of tumor cells but also directly modulates gene expression and protein function through microenvironmental acidification and the induction of lactylation modification [13]. Meanwhile, lactate plays a pivotal role in regulating the biological functions of immune cells within the TME. In glioma, lactate can activate the cAMP signaling pathway in TAMs, thereby promoting the secretion of pro‐tumorigenic factors and further consolidating the immunosuppressive microenvironment [53]. Additionally, the accumulation of lactate can disrupt intracellular lactate gradients in T cells, interrupt metabolic processes, and lead to impaired T cell function [10]. Our study found that lactate inhibition decreases the levels of H4K12la, PD‐L1, and CSF1 in GBM cells, while decreasing the infiltration of immunosuppressive cells. The potential mechanisms may involve an intrinsic epigenetic mechanism in GBM cells and a direct extrinsic effect of lactate on immune cells in the TME. This lactate‐mediated metabolic crosstalk network establishes bidirectional regulation between glioma cells and immune cells, collectively driving malignant tumor progression [10, 13]. Consequently, global suppression of TME lactate is necessary to suppress tumor cell growth and restore immune cell function.

Building on previous evidence that histone H3K9 lactylation may influence temozolomide resistance and can be targeted by Stiripentol [54], this study further identifies that lactylation at H4K12 is prominent in metabolically reprogrammed glioma cells. Stiripentol administration markedly reduced H4K12la level, restored PTEN expression, and suppressed PI3K/AKT signaling, leading to reduced tumor cell proliferation and invasion. Concomitantly, diminished H4K12la lowered PD‐L1 and CSF1 expression, thereby alleviating immunosuppressive features of the TME. These results provide an example of how metabolic modulation simultaneously affect epigenetic regulation and immune status in GBM.

Our study reveals that Stiripentol remodels the immunosuppressive microenvironment of GBM by reducing lactate levels, operating through both cell‐intrinsic mechanisms and microenvironmental extrinsic effects. Within GBM cells, decreased lactate levels lead to a reduction in H4K12la, thereby downregulating the expression of key immunosuppressive molecules such as PD‐L1 and CSF1. In the GBM microenvironment, Stiripentol shifts the pathologically high lactate concentration back toward the physiological range. This restoration directly alleviates the suppression of effector immune cells like CTLs and NK cells, while simultaneously abrogating the metabolic advantage of immunosuppressive cells such as Tregs and MDSCs. Consequently, Stiripentol significantly improves the immune cell balance and promotes the infiltration of antitumor immune cells. Although long‐term monotherapy may induce metabolic adaptive resistance, and its known off‐target effects (e.g., GABAergic modulation and cytochrome P450 enzyme inhibition) necessitate careful clinical management [55, 56, 57], combination therapy with anti‐PD‐1 antibodies and CSF1R inhibitors may achieve multi‐target synergistic efficacy and potentially delay the development of drug resistance.

Our findings provide integrated evidence linking transcriptional control, metabolic reprogramming, and immunosuppression through the RFX7‐PIK3IP1 axis. This combined analysis of clinical and experimental data offers a mechanistic framework to understand immune checkpoint resistance associated with lactate accumulation in GBM. Nevertheless, the current study is restricted to defined in vitro and in vivo models, and quantitative relationships among lactate concentration, histone lactylation, and target gene transcription require further clarification. Validation in larger and more diverse patient cohorts will be essential to confirm these mechanisms and determine their clinical relevance. Moreover, the potential cooperative effects between metabolic inhibitors and immune‐checkpoint blockade warrant systematic evaluation in preclinical and clinical studies.

Together, this work identifies an upstream transcriptional connection between lactate metabolism and immune regulation in GBM, providing a mechanistic framework for targeting metabolic‐immune interactions. These results highlight RFX7 as a key epigenetic and metabolic regulator and underscore the importance of integrating transcriptional and metabolic perspectives when addressing immune resistance in GBM.

## 4 Method and Materials

### 4.1 Patient Specimens

Paraffin‐embedded glioma samples (acquired from 2016/7 to 2017/1) with WHO II (n = 30), WHO III (n = 30), WHO IV (n = 30), and frozen glioma samples (obtained from 2020/1 to 2020/6) were obtained from the Department of Neurosurgery, Tangdu Hospital (Xi'an, China). Paraffin‐embedded glioma samples were collected for immunohistochemical staining, and frozen glioma samples were collected for Western blot, qPCR, and immunofluorescence staining. These patients were not treated with radiotherapy or any antitumor drugs prior to the surgery. All patients had prognostic follow‐up information. All patients had signed a written informed consent, and the protocol was also approved by the Ethics Committee of Tangdu Hospital of the Air Force Military Medical University.

### 4.2 Cell Proliferation Assays

Cell proliferation was detected by cell counting kit‐8 (Beyotime, C0038), EdU, and colony formation assays. In the CCK8 assay, we seeded 2 × 10^3^ cells per well in a 96‐well plate and cultured for 0–5 days. Cell viability was measured after adding 10 µL CCK8 reagent for 2 h. EdU staining proliferation assay was conducted by BeyoClick^TM^ EdU Cell Proliferation Kit (Beyotime, C00855) following the manufacture's protocol. In the colony formation assay, 200 cells per well were grown in 6‐well plates and cultured for 2 weeks. After fixation with 4% paraformaldehyde and staining with 0.1% crystal violet, photographs were then taken to count the cell colonies.

### 4.3 Cell Invasion Assay

5 × 10^4^ cells were transplanted into the upper chamber of the Transwell plates (8 µm pore size, 6.5 mm diameter) with 100 µL of DMEM without FBS, while the bottom chamber was filled with 500 µL of 20% FBS DMEM. After 48 h incubation, upper surface cells were removed, and lower surface cells were fixed with 4% paraformaldehyde and stained with Crystal Violet. After the removal of excess dye, we used an optical microscope to observe the cells and counted the cells throughout the filter.

### 4.4 Orthotopic Transplantation

All animal experiments were approved by the Committee for Experimental Animal Use and Care of the Air Force Military Medical University. This study followed the National Guidelines for the Experimentation of Animals in all animal experiments. Mice were anesthetized with 2% isoflurane (v/v) delivered in oxygen via a precision vaporizer and maintained on a heating pad at 37°C throughout the surgical procedure. Male BALB/c nude mice (6–8 weeks old) were implanted with 5 × 10^6^ U251 cells with modified gene expression. Male C57BL/6 mice (6–8 weeks old) were transplanted with the GL261‐luc cell line with modified gene expression.

### 4.5 Flow Cytometry

Tissue samples were collected from the tumor site, carefully removing surrounding brain tissue. Mince the tissue and grind it, then add papain to lyse the cells. After a 30 min reaction, add fetal bovine serum to terminate digestion. Subsequently, filter the cell suspension through a 100 µm cell strainer and add red blood cell lysis buffer to remove red blood cells, thereby obtaining a tumor single‐cell suspension. Add the desired antibodies to the single‐cell suspension and incubate in the dark for 2 h. Wash the cells with staining buffer. Finally, resuspend the cells in 300 µL of PBS containing 1% FBS. Data were analyzed using the ACEA NovoCyte flow cytometer and processed using NovoExpress 1.5.6 software.

### 4.6 RNA Library Sequencing

Total RNA was extracted using TRIzol reagent. Following quality assessment, mRNA was enriched, fragmented, and reverse‐transcribed into cDNA. The cDNA libraries were constructed through end repair, adenylation, and adapter ligation. After size selection and PCR amplification, the final libraries (insert size ∼300 bp) were paired‐end sequenced (2 × 150 bp) on an Illumina Novaseq 6000 platform. Differential expression analysis was performed with the *DESeq2* R package, applying a cutoff of absolute log2 fold‐change > 1.2 and an adjusted *p*‐value < 0.05.

### 4.7 Pyrosequencing for DNA methylation analysis

After extracting sample DNA using the QIAGEN Genomic DNA Extraction Kit, the DNA underwent bisulfite conversion using the Qiagen EpiTect Bisulfite Kit (Catalog No. 59104). Specific primers were designed using PyroMark Assay Design 2.0 software (primer sequences detailed in Supplemental Materials), followed by PCR amplification. The amplified products were subjected to pyrophosphate sequencing analysis on the Pyrosequencing Q48 system. Finally, the methylation levels at each CpG site were automatically calculated using the instrument's integrated Pyro Q‐CpG software.

### 4.8 Dual Luciferase Assay

Adherent cells in a 96‐well plate were lysed on ice for 5 min using 100 µL of lysis buffer. Following lysis, 20 µL of the lysate was transferred to a black microplate. Firefly luciferase activity was measured immediately after adding 100 µL of room‐temperature firefly luciferase working solution and mixing. Subsequently, 100 µL of Renilla luciferase working solution was added to the same well. After mixing and a 10 min incubation, Renilla luciferase activity was measured. The relative reporter gene activation was calculated as the ratio of firefly to Renilla luciferase activity. The primer sequences of the promoter region were detailed in the Supplemental Materials

### 4.9 ChIP‐seq and CUT&Tag

ChIP‐seq (Chromatin Immunoprecipitation followed by sequencing) and CUT&Tag (Cleavage Under Targets and Tagmentation) were used to study protein‐DNA interactions. ChIP‐seq involves cross‐linking proteins to DNA with formaldehyde, fragmenting chromatin via sonication, immunoprecipitating target protein‐bound DNA fragments with specific antibodies, and sequencing the purified DNA. CUT&Tag utilizes a protein A/G‐Tn5 transposase fusion complex guided by antibodies to target protein‐bound genomic regions. This complex simultaneously cleaves DNA and inserts sequencing adapters in situ. This process allows for efficient library construction directly from permeabilized cells.

### 4.10 Nontargeted Metabolomics Sequencing

Nontargeted metabolomics employs an unbiased detection strategy to systematically analyze the dynamic changes in all detectable small‐molecule metabolites by liquid chromatography‐mass spectrometry (LC‐MS). The standard workflow involves the extraction of metabolites with prechilled organic solvents to achieve instantaneous enzyme inactivation, followed by chromatographic separation and high‐resolution mass spectrometry. Finally, bioinformatics methods were applied to process the data for metabolite identification.

### 4.11 L‐Lactate and ECAR Detection Assays

Intracellular L‐lactate detection assay using the WST‐8 method quantifies lactate levels based on an enzymatic‐colorimetric principle: L‐lactate is oxidized by L‐lactate dehydrogenase (L‐LDH), generating NADH, which subsequently reduces WST‐8 to a formazan dye with absorbance at 450 nm proportional to lactate concentration. The Agilent Seahorse Energy Metabolism Analyzer determines the ECAR by measuring changes in the pH of the cell culture medium. During the assay, cells were exposed to specific metabolic substrates. The subsequent glycolytic activity of the cells leads to the production and secretion of lactate and hydrogen ions, which acidifies the surrounding medium, causing a measurable drop in pH. Based on the measured rate of pH change, the instrument automatically calculates the ECAR, providing a real‐time indicator of glycolytic flux.

### 4.12 Bioinformatic Analysis

All bioinformatic analysis was conducted with R 4.0.3 (https://www.rproject.org/). Following the identification of prognostic differentially expressed genes (DEGs), their associated biological functions and pathways were explored using Gene Ontology (GO), Kyoto Encyclopedia of Genes and Genomes (KEGG), and Gene Set Enrichment Analysis (GSEA). The tumor immune microenvironment was further characterized by estimating stromal and immune presence with the ESTIMATE algorithm and quantifying immune cell infiltration levels with CIBERSORT and ssGSEA.

### 4.13 Statistical Analysis

Data preprocessing was performed to ensure reliability. Data are presented as the mean ± standard deviation (SD). The sample size (n) refers to the number of independent cell culture experiments or the number of animals used in each group. Animals were randomly assigned to experimental groups using a randomization sequence generated with Microsoft Excel. Investigators involved in data collection and analysis were blinded to group allocations. Statistical significance was evaluated using two‐sided Student's *t*‐test, one‐way ANOVA, two‐way ANOVA, or log‐rank test as appropriate. For ANOVA, post‐hoc multiple comparisons were performed where necessary. *P* value < 0.05 was considered statistically significant. All statistical assumptions (normal distribution and homogeneity of variance) were tested and satisfied prior to analysis. Statistical analyses were performed using SPSS 22.0 and R version 4.0.3.

## Funding

This work was supported by the National Key Research and Development Program of China (2023YFC2510002), National Natural Science Foundation of China (82430039) and the Fund of Tangdu Hospital (N0.2025JCRH030).

## Ethics Statement

The studies involving human samples were performed in accordance with the Declaration of Helsinki and approved by the Medical Ethics Committee of Tangdu Hospital (Approval No. GKJ‐Y‐202503‐078). Written informed consent was obtained from all enrolled patients. All animal experiments were conducted in accordance with the National Institutes of Health Guide for the Care and Use of Laboratory Animals and approved by the Committee for Experimental Animal Care and Use of Air Force Medical University (Approval No. 251301).

## Conflicts of Interest

The authors declared that the research was conducted in the absence of any commercial or financial relationships that could be construed as a potential conflicts of interest.

## Supporting information




**Supporting File 1**: advs75761‐sup‐0001‐SuppMat.docx.

**Supporting File 2**: advs75761‐sup‐0002‐FigureS1.pdf.

**Supporting File 3**: advs75761‐sup‐0003‐FigureS2.pdf.

**Supporting File 4**: advs75761‐sup‐0004‐FigureS3.pdf.

**Supporting File 5**: advs75761‐sup‐0005‐FigureS4.pdf.

**Supporting File 6**: advs75761‐sup‐0006‐FigureS5.pdf.

**Supporting File 7**: advs75761‐sup‐0007‐FigureS6.pdf.

**Supporting File 8**: advs75761‐sup‐0008‐FigureS7.pdf.

## Data Availability

The raw data supporting the conclusions of this article will be made available by the authors, without undue reservation. The TCGA database for this study can be downloaded from https://portal.gdc.cancer.gov/. The CGGA database for this study can be downloaded from http://www.cgga.org.cn/. The public single‐cell datasets include GBmap, whose data is available via CZ CELLxGENE Discover (https://cellxgene.cziscience.com/collections/999f2a15‐3d7e‐440b‐96ae‐2c806799c08c), and CNP0003766, whose data is available via the original publication. The raw data of ChIP‐seq and RNA‐seq in our study have been deposited in the National Genomics Data Center (NGDC) database (https://ngdc.cncb.ac.cn) under the accession number HRA017652.
