# Supplementary material for: Epigenetic Silencing of RFX7 Defines a Transcriptional Axis Linking Lactate Metabolism to Immune Checkpoint Therapy in Glioblastoma
Source: Adv Sci (Weinh). 2026 May 28;13(45):e23792. doi: 10.1002/advs.202523792 (PMC13336137; doi:10.1002/advs.202523792)
Supplement: Supplementary file 1 [file ADVS-13-e23792-s001.pdf]

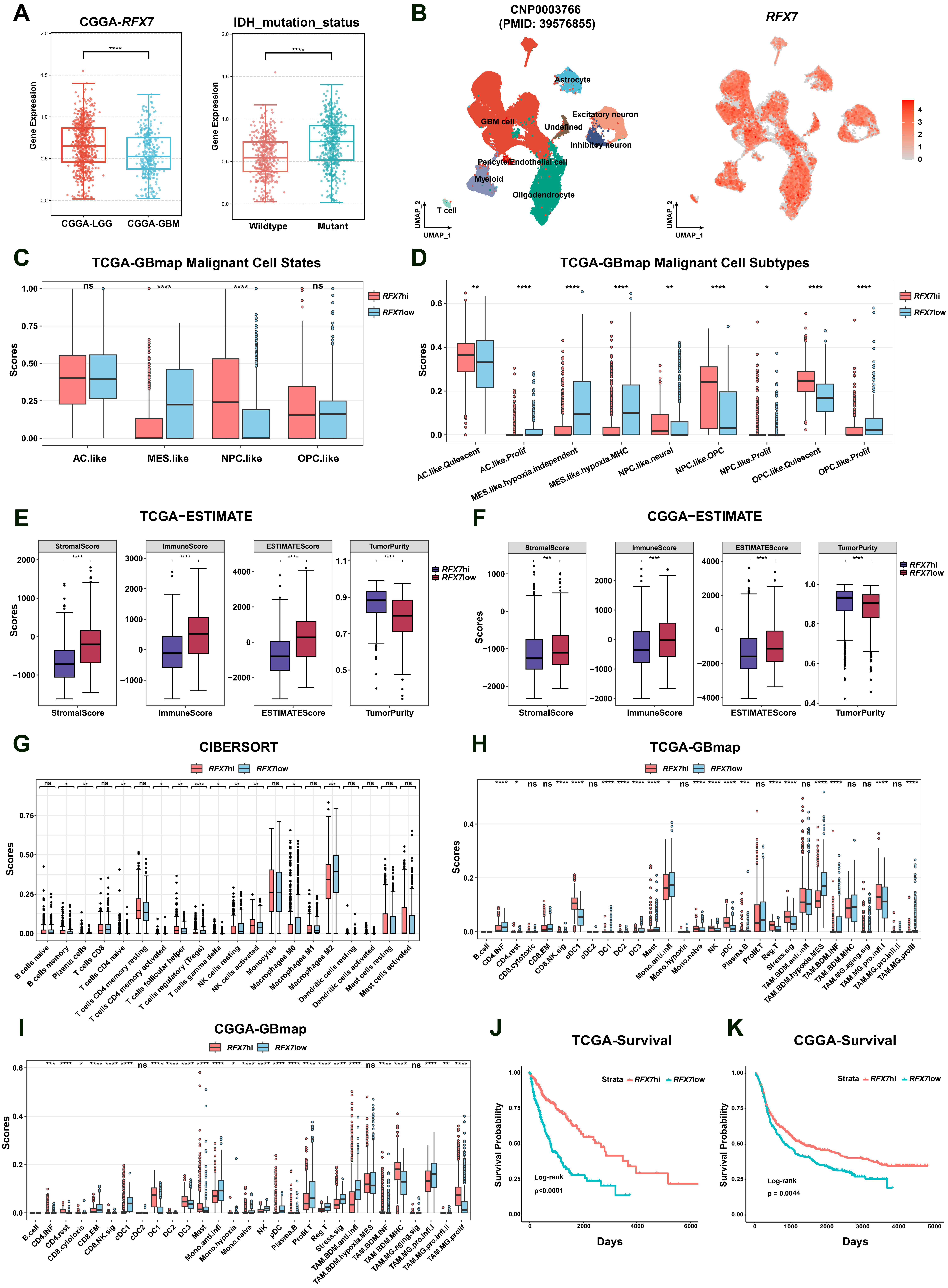
A
CGGA-RFX7
IDH_mutation_status
Gene Expression
CGGA-LGG
CGGA-GBM
Wildtype
Mutant
B
CNP0003766
(PMID: 39576855)
RFX7
Astrocyte
Excitatory neuron
GBM cell
Undefined
Inhibitory neuron
Pericyte/Endothelial cell
Myeloid
Oligodendrocyte
T cell
UMAP_1
UMAP_2
C
TCGA-GBmap Malignant Cell States
Scores
RFX7hi
RFX7low
AC.like
MES.like
NPC.like
OPC.like
D
TCGA-GBmap Malignant Cell Subtypes
AC.like.Quiescent
AC.like.Prolif
MES.like.hypoxia.independent
MES.like.hypoxia.MHC
NPC.like.neural
NPC.like.OPC
NPC.like.Prolif
OPC.like.Quiescent
OPC.like.Prolif
E
TCGA−ESTIMATE
StromalScore
ImmuneScore
ESTIMATEScore
TumorPurity
F
CGGA−ESTIMATE
G
CIBERSORT
B cells naive
B cells memory
Plasma cells
T cells CD8
T cells CD4 naive
T cells CD4 memory resting
T cells CD4 memory activated
T cells follicular helper
T cells regulatory (Tregs)
T cells gamma delta
NK cells resting
NK cells activated
Monocytes
Macrophages M0
Macrophages M1
Macrophages M2
Dendritic cells resting
Dendritic cells activated
Mast cells resting
Mast cells activated
H
TCGA-GBmap
B.cell
CD4.INF
CD4.rest
CD8.cytotoxic
CD8.EM
CD8.NK.sig
cDC1
cDC2
DC1
DC2
DC3
Mast
Mono.anti.infl
Mono.hypoxia
Mono.naive
NK
pDC
Plasma.B
Prolif.T
Reg.T
Stress.sig
TAM.BDM.anti.infl
TAM.BDM.hypoxia.MES
TAM.BDM.INF
TAM.BDM.MHC
TAM.MG.aging.sig
TAM.MG.pro.infl.I
TAM.MG.pro.infl.II
TAM.MG.prolif
I
CGGA-GBmap
J
TCGA-Survival
Strata
Survival Probability
Log-rank
p<0.0001
Days
K
CGGA-Survival
Log-rank
p = 0.0044
