# Supplementary material for: Epigenetic Silencing of RFX7 Defines a Transcriptional Axis Linking Lactate Metabolism to Immune Checkpoint Therapy in Glioblastoma
Source: Adv Sci (Weinh). 2026 May 28;13(45):e23792. doi: 10.1002/advs.202523792 (PMC13336137; doi:10.1002/advs.202523792)
Supplement: Supplementary file 2 [file ADVS-13-e23792-s002.pdf]

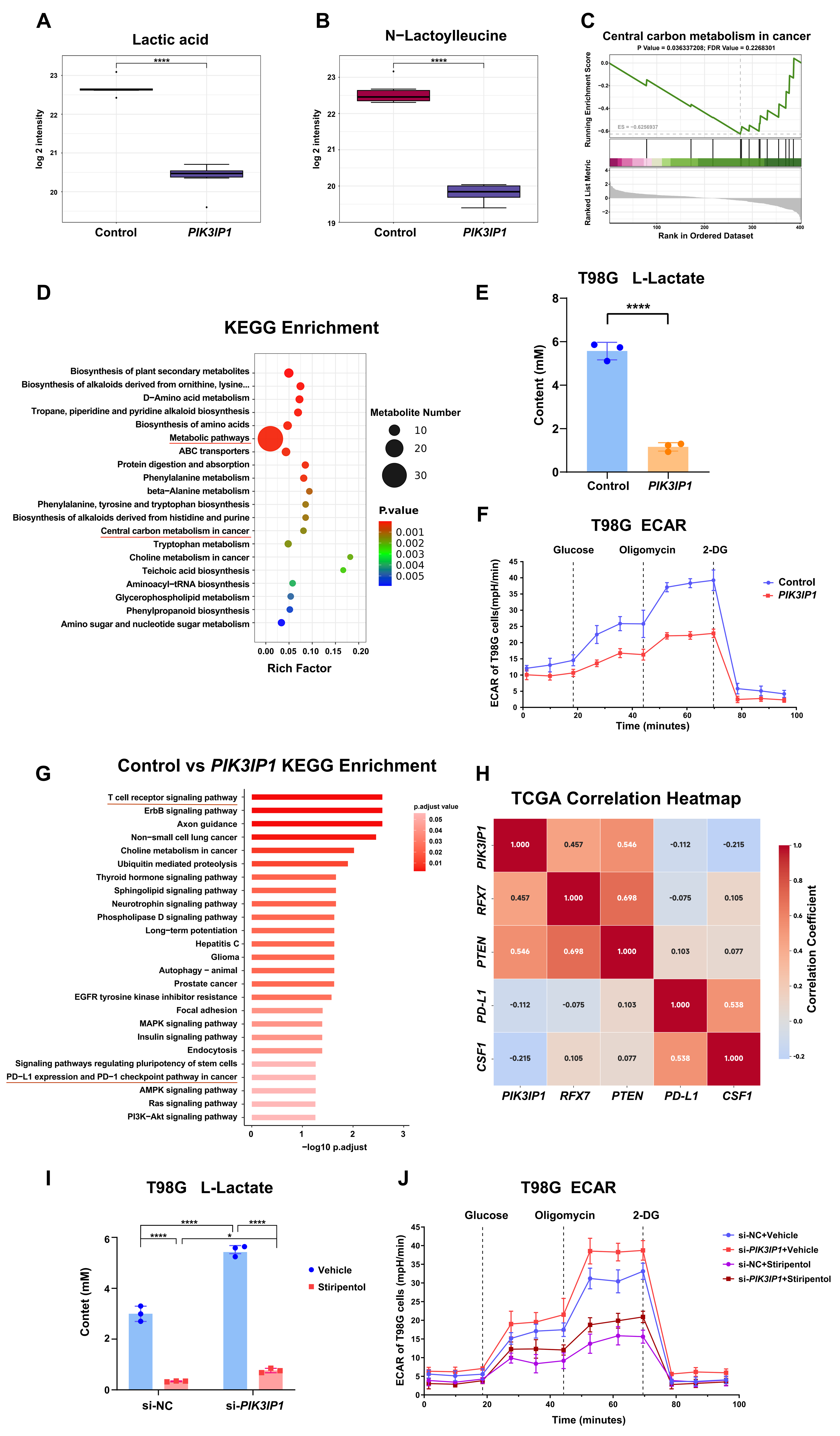

A
Lactic acid
log 2 intensity
Control
PIK3IP1
B
N−Lactoylleucine
log 2 intensity
Control
PIK3IP1
C
Central carbon metabolism in cancer
P Value = 0.036337208; FDR Value = 0.2268301
Running Enrichment Score
ES = −0.6256937
Ranked List Metric
Rank in Ordered Dataset
D
KEGG Enrichment
Biosynthesis of plant secondary metabolites
Biosynthesis of alkaloids derived from ornithine, lysine...
D−Amino acid metabolism
Tropane, piperidine and pyridine alkaloid biosynthesis
Biosynthesis of amino acids
Metabolic pathways
ABC transporters
Protein digestion and absorption
Phenylalanine metabolism
beta−Alanine metabolism
Phenylalanine, tyrosine and tryptophan biosynthesis
Biosynthesis of alkaloids derived from histidine and purine
Central carbon metabolism in cancer
Tryptophan metabolism
Choline metabolism in cancer
Teichoic acid biosynthesis
Aminoacyl−tRNA biosynthesis
Glycerophospholipid metabolism
Phenylpropanoid biosynthesis
Amino sugar and nucleotide sugar metabolism
Metabolite Number
P.value
Rich Factor
E
T98G L-Lactate
Content (mM)
Control
PIK3IP1
F
T98G ECAR
Glucose
Oligomycin
2-DG
ECAR of T98G cells(mpH/min)
Time (minutes)
Control
PIK3IP1
G
Control vs PIK3IP1 KEGG Enrichment
T cell receptor signaling pathway
ErbB signaling pathway
Axon guidance
Non−small cell lung cancer
Choline metabolism in cancer
Ubiquitin mediated proteolysis
Thyroid hormone signaling pathway
Sphingolipid signaling pathway
Neurotrophin signaling pathway
Phospholipase D signaling pathway
Long−term potentiation
Hepatitis C
Glioma
Autophagy − animal
Prostate cancer
EGFR tyrosine kinase inhibitor resistance
Focal adhesion
MAPK signaling pathway
Insulin signaling pathway
Endocytosis
Signaling pathways regulating pluripotency of stem cells
PD−L1 expression and PD−1 checkpoint pathway in cancer
AMPK signaling pathway
Ras signaling pathway
PI3K−Akt signaling pathway
p.adjust value
−log10 p.adjust
H
TCGA Correlation Heatmap
PIK3IP1
RFX7
PTEN
PD-L1
CSF1
Correlation Coefficient
I
T98G L-Lactate
Contet (mM)
Vehicle
Stiripentol
si-NC
si-PIK3IP1
J
T98G ECAR
Glucose
Oligomycin
2-DG
ECAR of T98G cells (mpH/min)
Time (minutes)
si-NC+Vehicle
si-PIK3IP1+Vehicle
si-NC+Stiripentol
si-PIK3IP1+Stiripentol
