# Supplementary material for: Epigenetic Silencing of RFX7 Defines a Transcriptional Axis Linking Lactate Metabolism to Immune Checkpoint Therapy in Glioblastoma
Source: Adv Sci (Weinh). 2026 May 28;13(45):e23792. doi: 10.1002/advs.202523792 (PMC13336137; doi:10.1002/advs.202523792)
Supplement: Supplementary file 3 [file ADVS-13-e23792-s003.pdf]

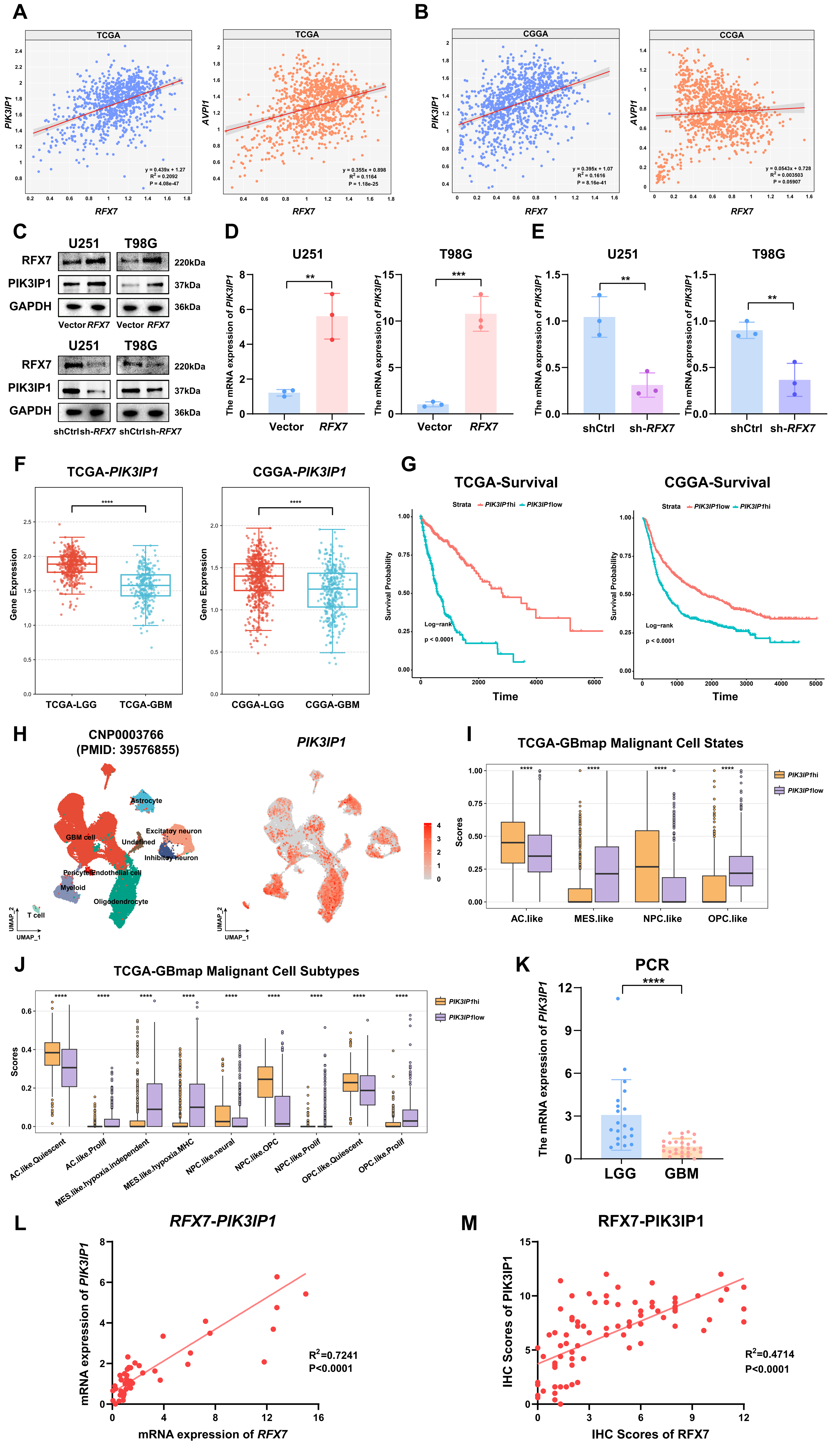

A
TCGA
PIK3IP1
RFX7
y = 0.439x + 1.27
R^2 = 0.2092
P = 4.08e-47
TCGA
AVPI1
RFX7
y = 0.355x + 0.898
R^2 = 0.1164
P = 1.18e-25
B
CGGA
PIK3IP1
RFX7
y = 0.395x + 1.07
R^2 = 0.1616
P = 8.16e-41
CCGA
AVPI1
RFX7
y = 0.0543x + 0.728
R^2 = 0.003503
P = 0.05907
C
U251
T98G
RFX7
PIK3IP1
GAPDH
220kDa
37kDa
36kDa
Vector RFX7 Vector RFX7
shCtrl sh-RFX7 shCtrl sh-RFX7
D
U251
T98G
The mRNA expression of PIK3IP1
Vector
RFX7
E
U251
T98G
The mRNA expression of PIK3IP1
shCtrl
sh-RFX7
F
TCGA-PIK3IP1
CGGA-PIK3IP1
Gene Expression
TCGA-LGG
TCGA-GBM
CGGA-LGG
CGGA-GBM
G
TCGA-Survival
CGGA-Survival
Strata
PIK3IP1hi
PIK3IP1low
Survival Probability
Log-rank
p < 0.0001
Time
H
CNP0003766
(PMID: 39576855)
Astrocyte
Excitatory neuron
GBM cell
Undefined
Inhibitory neuron
Pericyte/Endothelial cell
Myeloid
Oligodendrocyte
T cell
UMAP_1
UMAP_2
PIK3IP1
I
TCGA-GBmap Malignant Cell States
Scores
AC.like
MES.like
NPC.like
OPC.like
PIK3IP1hi
PIK3IP1low
J
TCGA-GBmap Malignant Cell Subtypes
Scores
AC.like.Quiescent
AC.like.Prolif
MES.like.hypoxia.independent
MES.like.hypoxia.MHC
NPC.like.neural
NPC.like.OPC
NPC.like.Prolif
OPC.like.Quiescent
OPC.like.Prolif
K
PCR
The mRNA expression of PIK3IP1
LGG
GBM
L
RFX7-PIK3IP1
mRNA expression of PIK3IP1
mRNA expression of RFX7
R^2=0.7241
P<0.0001
M
RFX7-PIK3IP1
IHC Scores of PIK3IP1
IHC Scores of RFX7
R^2=0.4714
P<0.0001
