# Supplementary material for: Epigenetic Silencing of RFX7 Defines a Transcriptional Axis Linking Lactate Metabolism to Immune Checkpoint Therapy in Glioblastoma
Source: Adv Sci (Weinh). 2026 May 28;13(45):e23792. doi: 10.1002/advs.202523792 (PMC13336137; doi:10.1002/advs.202523792)
Supplement: Supplementary file 4 [file ADVS-13-e23792-s004.pdf]

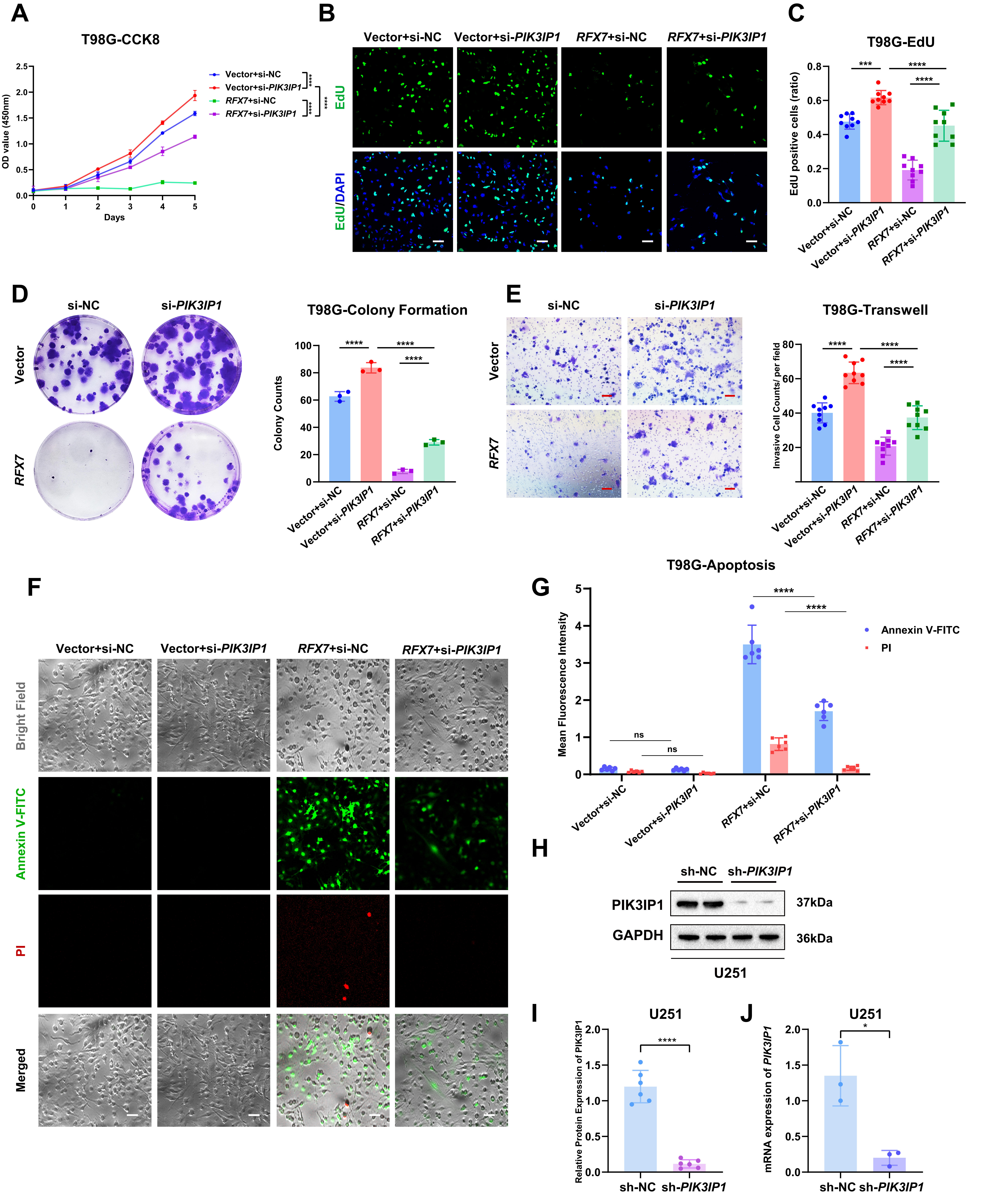

A
T98G-CCK8
OD value (450nm)
Days
Vector+si-NC
Vector+si-PIK3IP1
RFX7+si-NC
RFX7+si-PIK3IP1
B
Vector+si-NC
Vector+si-PIK3IP1
RFX7+si-NC
RFX7+si-PIK3IP1
EdU
EdU/DAPI
C
T98G-EdU
EdU positive cells (ratio)
D
si-NC
si-PIK3IP1
Vector
RFX7
T98G-Colony Formation
Colony Counts
E
si-NC
si-PIK3IP1
Vector
RFX7
T98G-Transwell
Invasive Cell Counts/ per field
F
Vector+si-NC
Vector+si-PIK3IP1
RFX7+si-NC
RFX7+si-PIK3IP1
Bright Field
Annexin V-FITC
PI
Merged
G
T98G-Apoptosis
Mean Fluorescence Intensity
Annexin V-FITC
PI
ns
H
sh-NC
sh-PIK3IP1
PIK3IP1
GAPDH
37kDa
36kDa
U251
I
U251
Relative Protein Expression of PIK3IP1
sh-NC
sh-PIK3IP1
J
U251
mRNA expression of PIK3IP1
sh-NC
sh-PIK3IP1
