# Supplementary material for: Epigenetic Silencing of RFX7 Defines a Transcriptional Axis Linking Lactate Metabolism to Immune Checkpoint Therapy in Glioblastoma
Source: Adv Sci (Weinh). 2026 May 28;13(45):e23792. doi: 10.1002/advs.202523792 (PMC13336137; doi:10.1002/advs.202523792)
Supplement: Supplementary file 5 [file ADVS-13-e23792-s006.pdf]

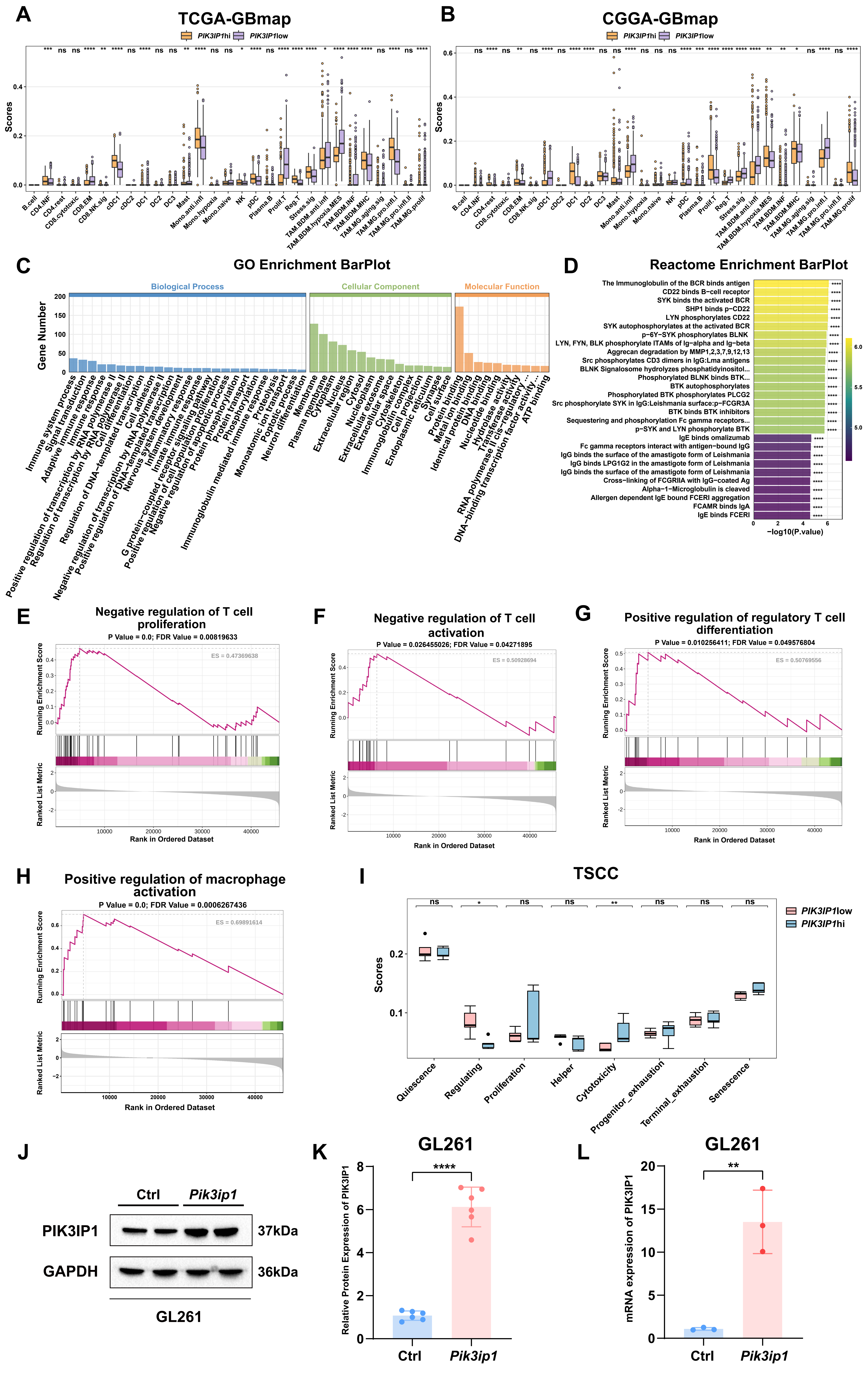
A
TCGA-GBmap
PIK3IP1hi PIK3IP1low
Scores
B
CGGA-GBmap
PIK3IP1hi PIK3IP1low
Scores
C
GO Enrichment BarPlot
Biological Process
Cellular Component
Molecular Function
Gene Number
D
Reactome Enrichment BarPlot
The Immunoglobulin of the BCR binds antigen
CD22 binds B-cell receptor
SYK binds the activated BCR
SHP1 binds p-CD22
LYN phosphorylates CD22
SYK autophosphorylates at the activated BCR
p-6Y-SYK phosphorylates BLNK
LYN, FYN, BLK phosphorylate ITAMs of Ig-alpha and Ig-beta
Aggrecan degradation by MMP1,2,3,7,9,12,13
Src phosphorylates CD3 dimers in IgG:Lma antigens
BLNK Signalosome hydrolyzes phosphatidyinositol...
Phosphorylated BLNK binds BTK...
BTK autophosphorylates
Phosphorylated BTK phosphorylates PLCG2
Src phosphorylate SYK in IgG:Leishmania surface:p-FCGR3A
BTK binds BTK inhibitors
Sequestering and phosphorylation Fc gamma receptors...
p-SYK and LYN phosphorylate BTK
IgE binds omalizumab
Fc gamma receptors interact with antigen-bound IgG
IgG binds the surface of the amastigote form of Leishmania
IgG binds LPG1G2 in the amastigote form of Leishmania
IgG binds the surface of the amastigote form of Leishmania
Cross-linking of FCGRIIA with IgG-coated Ag
Alpha-1-Microglobulin is cleaved
Allergen dependent IgE bound FCERI aggregation
FCAMR binds IgA
IgE binds FCERI
-log10(P.value)
E
Negative regulation of T cell proliferation
P Value = 0.0; FDR Value = 0.00819633
ES = 0.47369638
Running Enrichment Score
Ranked List Metric
Rank in Ordered Dataset
F
Negative regulation of T cell activation
P Value = 0.026455026; FDR Value = 0.04271895
ES = 0.50928694
G
Positive regulation of regulatory T cell differentiation
P Value = 0.010256411; FDR Value = 0.049576804
ES = 0.50769556
H
Positive regulation of macrophage activation
P Value = 0.0; FDR Value = 0.0006267436
ES = 0.69891614
I
TSCC
PIK3IP1low
PIK3IP1hi
Scores
Quiescence
Regulating
Proliferation
Helper
Cytotoxicity
Progenitor_exhaustion
Terminal_exhaustion
Senescence
J
Ctrl Pik3ip1
PIK3IP1 37kDa
GAPDH 36kDa
GL261
K
GL261
Relative Protein Expression of PIK3IP1
****
Ctrl Pik3ip1
L
GL261
mRNA expression of PIK3IP1
**
Ctrl Pik3ip1
