# Supplementary material for: Epigenetic Silencing of RFX7 Defines a Transcriptional Axis Linking Lactate Metabolism to Immune Checkpoint Therapy in Glioblastoma
Source: Adv Sci (Weinh). 2026 May 28;13(45):e23792. doi: 10.1002/advs.202523792 (PMC13336137; doi:10.1002/advs.202523792)
Supplement: Supplementary file 6 [file ADVS-13-e23792-s007.pdf]

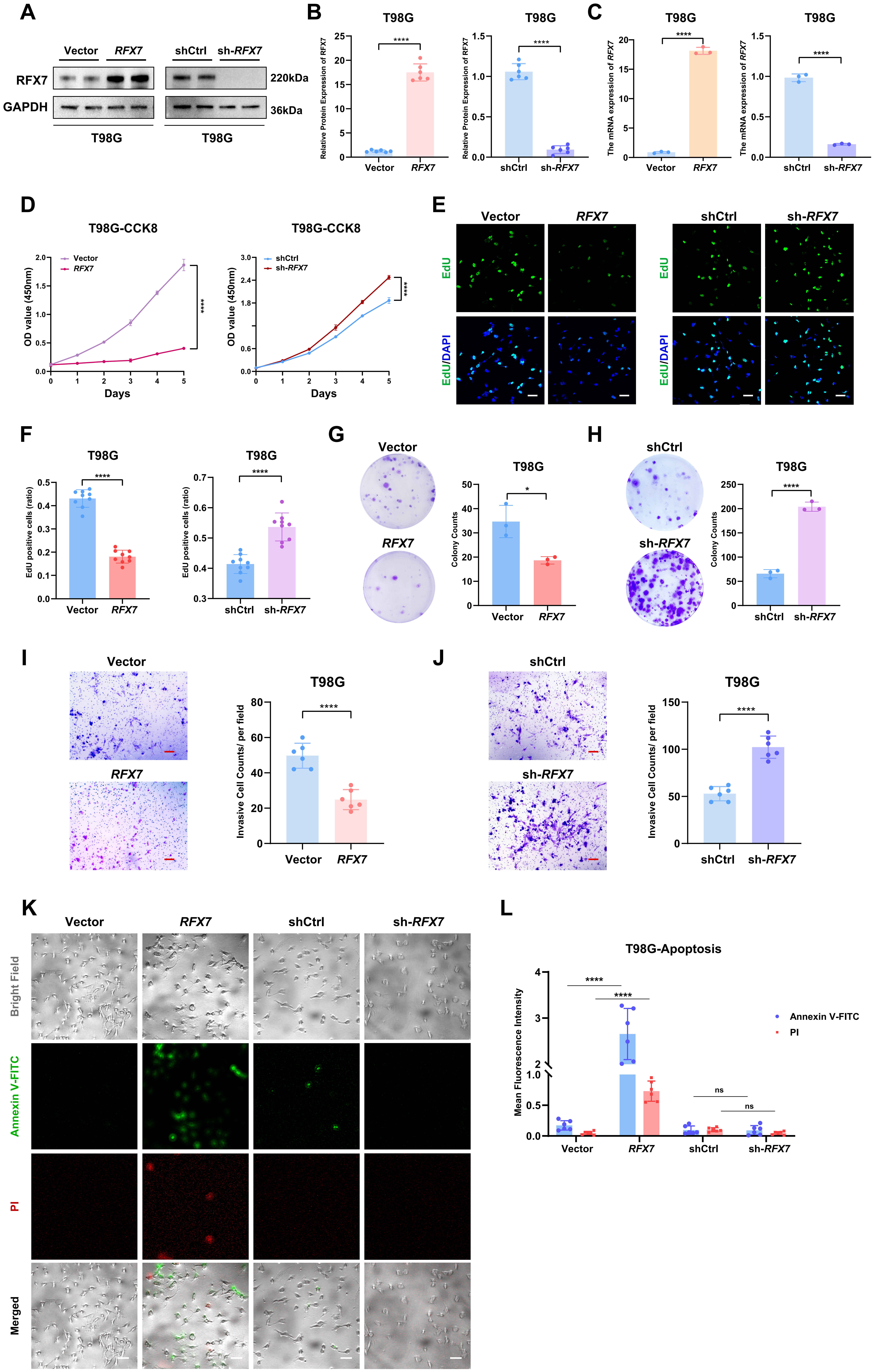
A
Vector
RFX7
shCtrl
sh-RFX7
RFX7
GAPDH
220kDa
36kDa
T98G
T98G
B
T98G
Relative Protein Expression of RFX7
Vector
RFX7
shCtrl
sh-RFX7
C
T98G
The mRNA expression of RFX7
D
T98G-CCK8
OD value (450nm)
Days
E
EdU
EdU/DAPI
F
EdU positive cells (ratio)
G
Colony Counts
H
I
Invasive Cell Counts/ per field
J
K
Bright Field
Annexin V-FITC
PI
Merged
L
T98G-Apoptosis
Mean Fluorescence Intensity
Annexin V-FITC
PI
ns
