# Supplementary material for: Epigenetic Silencing of RFX7 Defines a Transcriptional Axis Linking Lactate Metabolism to Immune Checkpoint Therapy in Glioblastoma
Source: Adv Sci (Weinh). 2026 May 28;13(45):e23792. doi: 10.1002/advs.202523792 (PMC13336137; doi:10.1002/advs.202523792)
Supplement: Supplementary file 7 [file ADVS-13-e23792-s008.pdf]

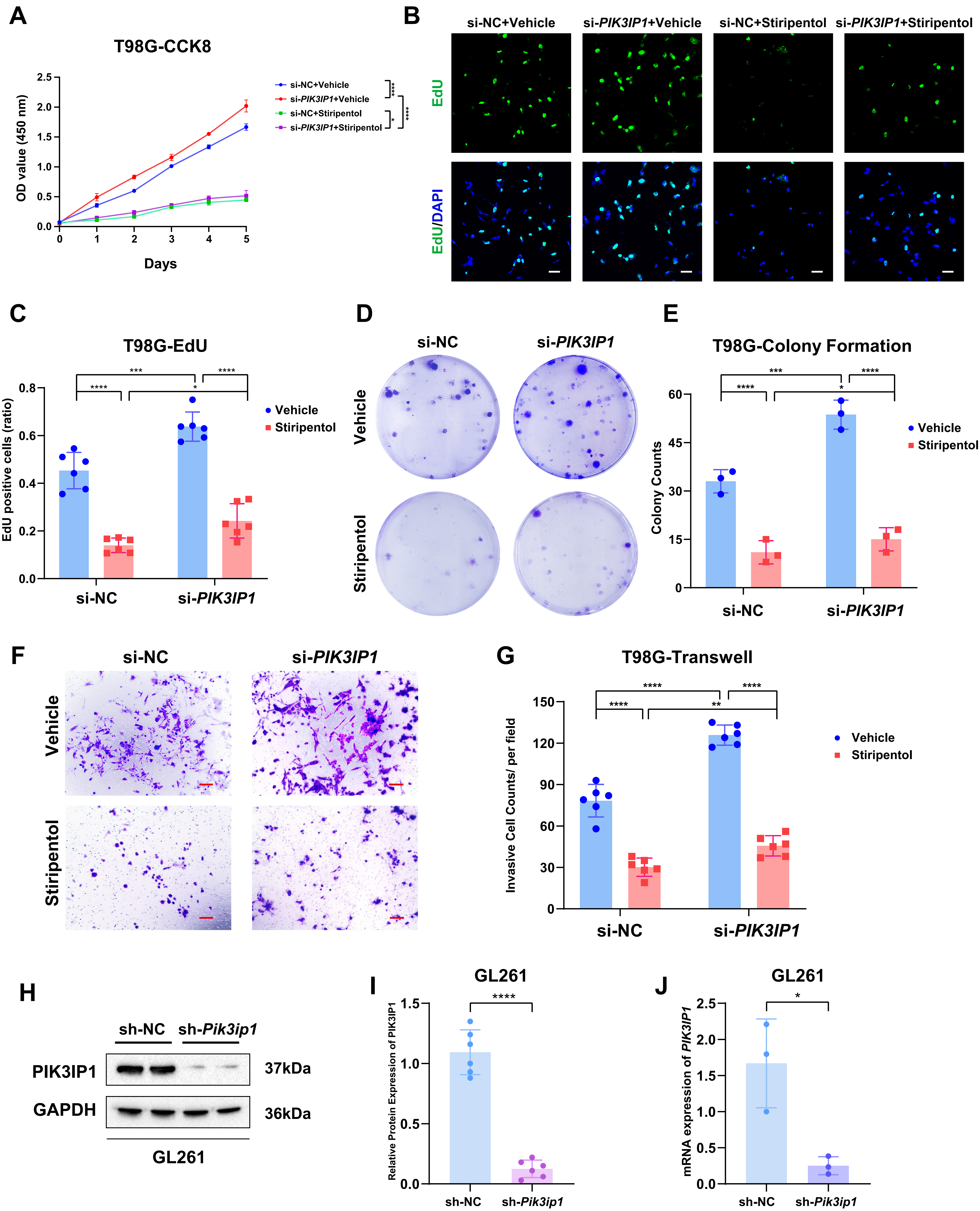
A
T98G-CCK8
OD value (450 nm)
Days
si-NC+Vehicle
si-PIK3IP1+Vehicle
si-NC+Stiripentol
si-PIK3IP1+Stiripentol
B
si-NC+Vehicle
si-PIK3IP1+Vehicle
si-NC+Stiripentol
si-PIK3IP1+Stiripentol
EdU
EdU/DAPI
C
T98G-EdU
EdU positive cells (ratio)
Vehicle
Stiripentol
si-NC
si-PIK3IP1
D
si-NC
si-PIK3IP1
Vehicle
Stiripentol
E
T98G-Colony Formation
Colony Counts
Vehicle
Stiripentol
si-NC
si-PIK3IP1
F
si-NC
si-PIK3IP1
Vehicle
Stiripentol
G
T98G-Transwell
Invasive Cell Counts/ per field
Vehicle
Stiripentol
si-NC
si-PIK3IP1
H
sh-NC
sh-Pik3ip1
PIK3IP1
GAPDH
37kDa
36kDa
GL261
I
GL261
Relative Protein Expression of PIK3IP1
sh-NC
sh-Pik3ip1
J
GL261
mRNA expression of PIK3IP1
sh-NC
sh-Pik3ip1
