# Supplementary material for: Epigenetic Silencing of RFX7 Defines a Transcriptional Axis Linking Lactate Metabolism to Immune Checkpoint Therapy in Glioblastoma
Source: Adv Sci (Weinh). 2026 May 28;13(45):e23792. doi: 10.1002/advs.202523792 (PMC13336137; doi:10.1002/advs.202523792)
Supplement: Supplementary file 8 [file ADVS-13-e23792-s005.docx]

**Figure S1 Relative Analyses of *RFX7* in Public Databases**

A. Expression of *RFX7* in LGG/GBM and IDH wildtype/mutant gliomas in CGGA (merged by CGGA-693 and CGGA-325) database (Student's t-test).

B. UMAP visualization of the *RFX7* expression in CNP0003766 single-cell atlas.

C-D. GBmap-based cell state signature scores and subpopulation scores (4 major states and 9 subtypes) stratified by *RFX7* expression levels in the TCGA database (Mann-Whitney U test).

E-F. ESTIMATE analysis of tumor purity at different *RFX7* expression levels in the TCGA (C) and CGGA (merged by CGGA-693 and CGGA-325) (D) databases (Mann-Whitney U test).

G. CIBERSORT analysis of immune infiltration comparing *RFX7*-high and *RFX7*-low GBM subgroups in the TCGA database (Mann-Whitney U test).

H-I. CIBERSORTx analysis of GBmap immune cell type profile comparing *RFX7*-high and *RFX7*-low GBM subgroups in the TCGA (F) and CGGA (merged by CGGA-693 and CGGA-325) (G) databases (Mann-Whitney U test).

J-K. Analysis of survival curves stratified by *RFX7* expression in patients from the TCGA (H) and CGGA (merged by CGGA-693 and CGGA-325) (I) databases (Log-rank test).

All values are shown as the mean ± SD. ^*^p < 0.05, ^**^p < 0.01, ^***^p < 0.001, ^****^p < 0.0001, ns: not significant.

**Figure S2 RFX7 Modulates the Malignant Behaviors of T98G cells in vitro**

A-B. Efficacy of RFX7 overexpression (A) and knockdown (B) in T98G cells validated by WB analysis (Student's t-test; n = 6 per group).

C. The mRNA expression of *RFX7* in T98G cells after virus transfection (Student's t-test; n = 3 per group).

D. Alterations in the proliferation capacity of T98G cells after interference with RFX7 expression in the CCK8 assay (Two-way repeated measures ANOVA; 3 repeats at each time point).

E-F. Alterations in the DNA synthesis and proliferation capacity of T98G cells after interference with RFX7 expression in the EdU assay (Scale Bar = 20 μm; Student's t-test; n = 8 per group).

G-H. Alterations in the clonogenic potential of T98G cells following RFX7 expression interference in the colony formation assay (Student's t-test; n = 3 per group).

I-J. Alterations in the invasive capacity of T98G cells following RFX7 expression interference in the Transwell assay (Scale Bar = 20 μm; Student's t-test; n = 6 per group).

K-L. Annexin V-FITC/PI staining assay to assess the capacity of apoptosis in T98G cells (Scale Bar = 20 μm; Student's t-test; n = 6 per group).

All values are shown as the mean ± SD. ^*^p < 0.05, ^****^p < 0.0001, ns: not significant.

**Figure S3 RFX7 transcriptionally regulates PIK3IP1 expression**

A-B. Pearson correlation analyses of the relationships between *RFX7* and *PIK3IP1*, and between *RFX7* and *AVPI1*, in TCGA (A) and CGGA (merged by CGGA-693 and CGGA-325) (B) databases (Pearson correlation analysis).

C. WB analysis of the effects of RFX7 overexpression and knockdown on PIK3IP1 expression.

D-E. PCR analysis of the effects of RFX7 overexpression (D) and knockdown (E) on *PIK3IP1* expression (Student's t-test; n = 3 per group).

F. Expression of *PIK3IP1* in LGG and GBM in TCGA and CGGA (merged by CGGA-693 and CGGA-325) databases (Student's t-test).

G. Analysis of survival curves stratified by *PIK3IP1* expression in patients from the TCGA and CGGA (merged by CGGA-693 and CGGA-325) databases (Log-rank test).

H. UMAP visualization of the *PIK3IP1* expression in CNP0003766 single-cell atlas.

I-J. GBmap-based cell state signature scores and subpopulation scores (4 major states and 9 subtypes) stratified by *PIK3IP1* expression levels in the TCGA database (Mann-Whitney U test).

K. PCR analysis of *PIK3IP1* expression in LGG and GBM tissues (qPCR: Student's t-test; 20 LGG and 27 GBM samples).

L. Pearson correlation analysis of the relationship between the mRNA expression of *RFX7* and *PIK3IP1* (Pearson correlation analysis; n =47).

M. Pearson correlation analysis of the relationship between the IHC scores of RFX7 and PIK3IP1 (Pearson correlation analysis; n =90).

All values are shown as the mean ± SD. ^**^p < 0.01, ^***^p < 0.001, ^****^p < 0.0001.

**Figure S4 RFX7-PIK3IP1 axis Modulates the Malignant Phenotypes T98G cells and the Efficiency of sh-NC/*PIK3IP1* Transfection in U251 cells**

A. Cell proliferation measured by CCK-8 assay following modulation of RFX7 and PIK3IP1 expression in T98G cells (Two-way repeated measures ANOVA; 3 repeats at each time point).

B-C. Assessment of DNA synthesis and proliferative capacity by EdU assay following modulation of RFX7 and PIK3IP1 expression in T98G cells (Scale Bar = 20 μm; Two-way ANOVA; n =9 per group).

D. Assessment of clonogenic potential by colony formation assay following modulation of RFX7 and PIK3IP1 expression in T98G cells (Two-way ANOVA; n = 3 per group).

E. Cell invasion capacity determined by Transwell assay following modulation of RFX7 and PIK3IP1 expression in T98G cells (Scale Bar = 20 μm; Two-way ANOVA; n =9 per group).

F-G. Apoptosis status analyzed by Annexin V-FITC/PI staining following modulation of RFX7 and PIK3IP1 expression in T98G cells (Scale Bar = 20 μm; Two-way ANOVA; n = 6 per group).

H-I. Efficacy of PIK3IP1 knockdown in U251 cells validated by WB analysis (Student's t-test, n = 6 per group).

J. Efficacy of *PIK3IP1* knockdown in U251 cells validated by qPCR analysis (Student's t-tes, n = 3 per group).

All values are shown as the mean ± SD. *p < 0.05, ^***^p < 0.001, ^****^p < 0.0001, ns: not significant.

**Figure S5 Higher PIK3IP1 Expression Correlates with a Less Immunosuppressive TME Profile**

A-B. CIBERSORTx analysis of GBmap (PMID:40312969) immune cell type profile comparing *PIK3IP1*-high and *PIK3IP1*-low GBM subgroups in the TCGA (A) and CGGA (merged by CGGA-693 and CGGA-325) (B) databases (Mann-Whitney U test).

C. GO enrichment analysis of DEGs with hi- and low- *PIK3IP1* expression GBM samples.

D. Reactome enrichment analysis of DEGs with hi- and low- *PIK3IP1* expression GBM samples.

E-H. GSEA enrichment analysis plots of the proliferation and activation in different immune cells.

I. TCSS analysis of the status of different T cells with hi- and low- *PIK3IP1* expression GBM samples (Mann-Whitney U test).

J-L. Efficacy of PIK3IP1 overexpression in GL261 cells validated by WB (J-K) and qPCR (L) analyses (Student's t-test; WB: n = 6 per group; PCR: n = 3 per group).

All values are shown as the mean ± SD.*p < 0.05, **p < 0.01, ***p < 0.001, ****p < 0.0001, ns: not significant.

**Figure S6 PIK3IP1 Silencing Regulates Lactate Accumulation in GBM**

A-B. Relative abundance of lactic acid and N-lactoyleucine in the Control and *PIK3IP1* groups (Student's t-test, n = 6 per group).

C. GSEA enrichment analysis plot of “Central carbon metabolism in cancer” pathway from the metabolomic analysis.

D. Scatterplot of KEGG enrichment analysis for the differentially expressed metabolites from the metabolomic analysis.

E. Intracellular L-lactate concentration measured in the Control and *PIK3IP1* groups of T98G cells (Student's t-test, n = 3 per group).

F. Extracellular acidification rate measured in the Control and *PIK3IP1* groups of T98G cells.

G. KEGG pathway enrichment analysis of upregulated peaks from CUT&Tag sequencing.

H. Correlation analysis of *PIK3IP1*, *RFX7*, *PTEN*, *PD-L1* and *CSF1* in the TCGA database (Pearson correlation analysis).

I. Intracellular L-lactate concentration of T98G cells following PIK3IP1 interference and Stiripentol treatment (Two-way ANOVA; n = 3 per group).

J. Extracellular acidification rate of T98G cells following PIK3IP1 interference and Stiripentol treatment.

All values are shown as the mean ± SD. ^*^p < 0.05, ^***^p < 0.001, ^****^p < 0.0001.

**Figure S7 Stiripentol Reverse the Tumor Malignancy Induced by PIK3IP1 Silencing in T98G cells and the Efficiency of sh-NC/*Pik3ip1* Transfection in GL261 cells**

A. CCK8 assay for proliferation capacity of T98G cells following Stiripentol treatment after si-NC/*PIK3IP1* interference (Two-way repeated measures ANOVA，3 repeats at each time point).

B-C. Evaluation of DNA synthesis and proliferation by EdU assay in T98G cells following si-NC/*PIK3IP1* interference and Stiripentol administration (Scale Bar = 20 μm, Two-way ANOVA, n = 6 per group).

D-E. Clonogenic potential measured by colony formation assay in T98G cells following si-NC/*PIK3IP1* interference and Stiripentol administration (Two-way ANOVA, n = 3 per group).

F-G. Cell invasion capacity determined by Transwell assay in T98G cells following si-NC/*PIK3IP1* interference and Stiripentol administration (Scale Bar = 20 μm, Two-way ANOVA, n = 6 per group).

H-I. Efficacy of PIK3IP1 knockdown in GL261 cells validated by WB analysis (Student's t-test, n = 6 per group).

J. Efficacy of *PIK3IP1* knockdown in GL261 cells validated by qPCR analysis (Student's t-tes, n = 3 per group).

All values are shown as the mean ± SD. *p < 0.05, **p < 0.01, ***p < 0.001, ****p < 0.0001.
