# Supplementary figures and images for: Epigenetic Silencing of RFX7 Defines a Transcriptional Axis Linking Lactate Metabolism to Immune Checkpoint Therapy in Glioblastoma
Source: Adv Sci (Weinh). 2026 May 28:e23792. Online ahead of print. doi: 10.1002/advs.202523792 (PMC13336137; doi:10.1002/advs.202523792)

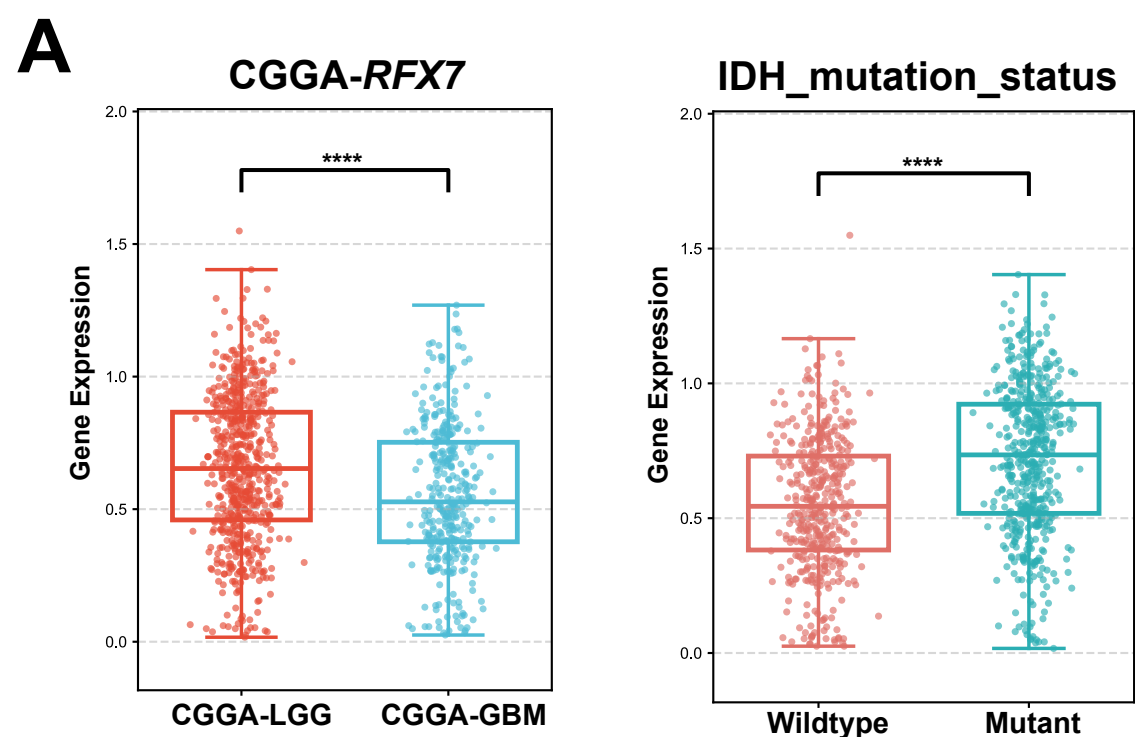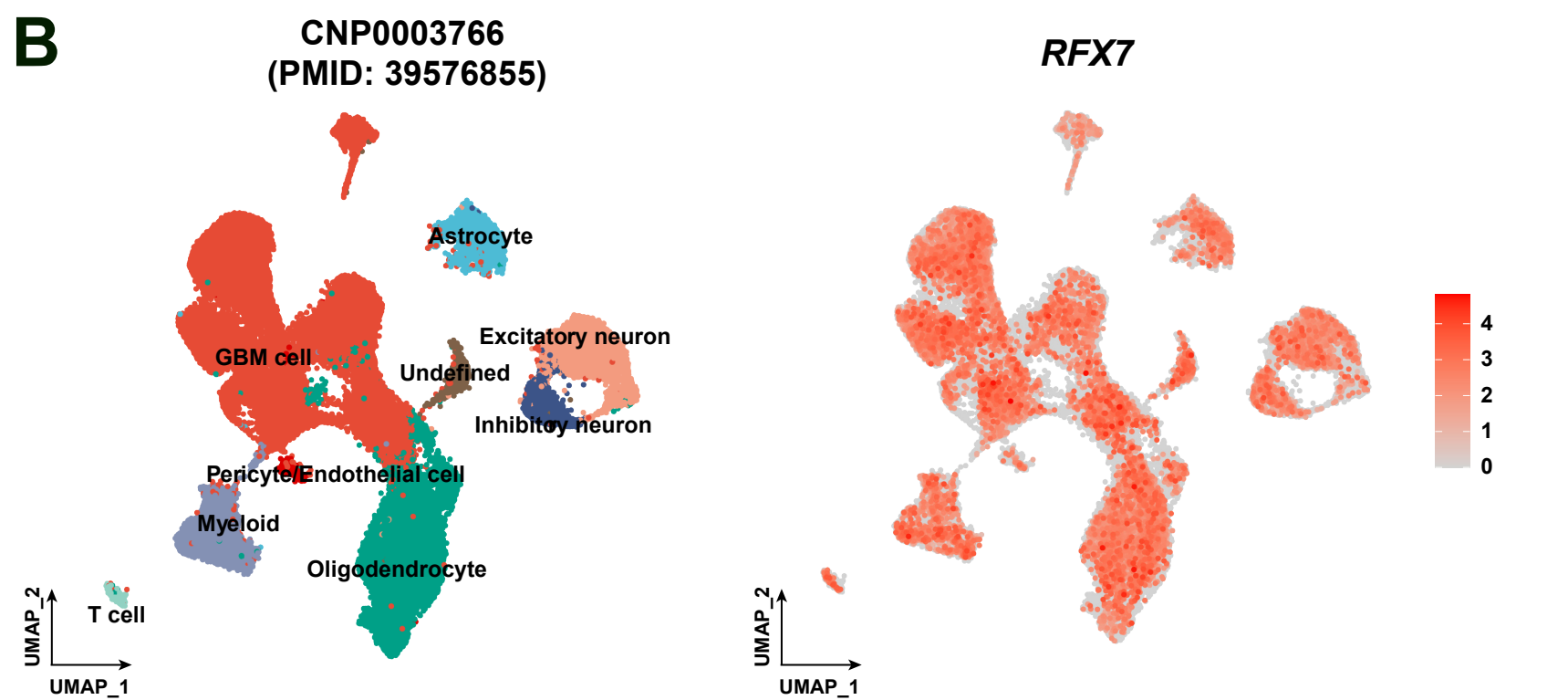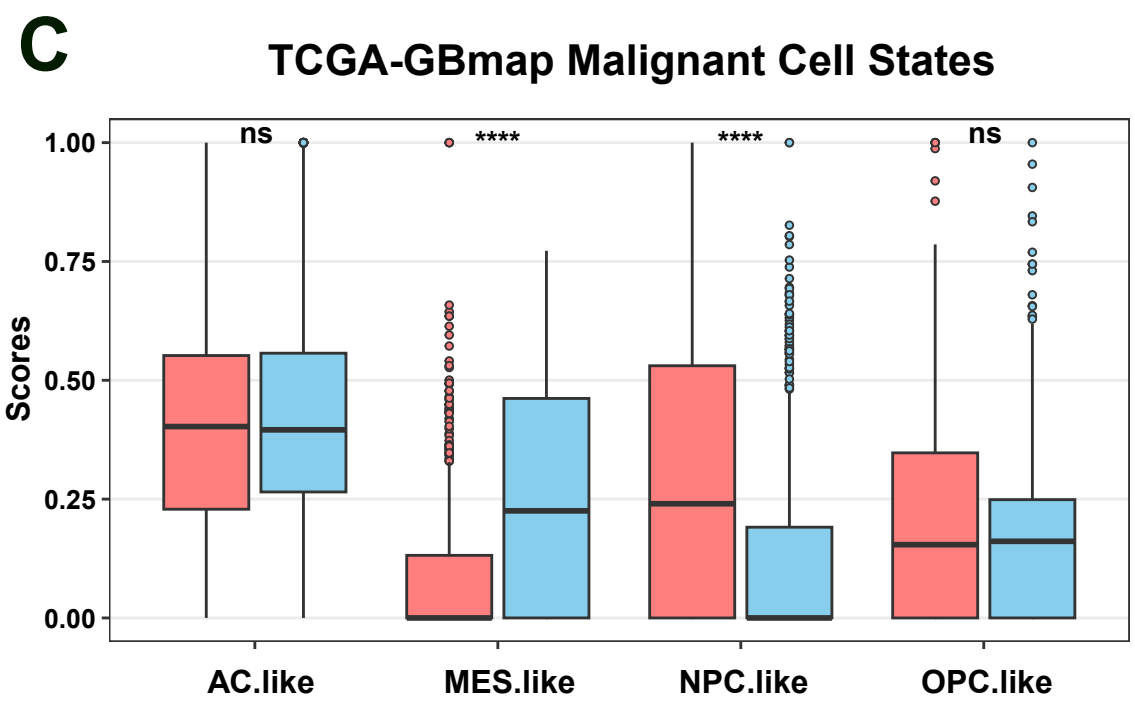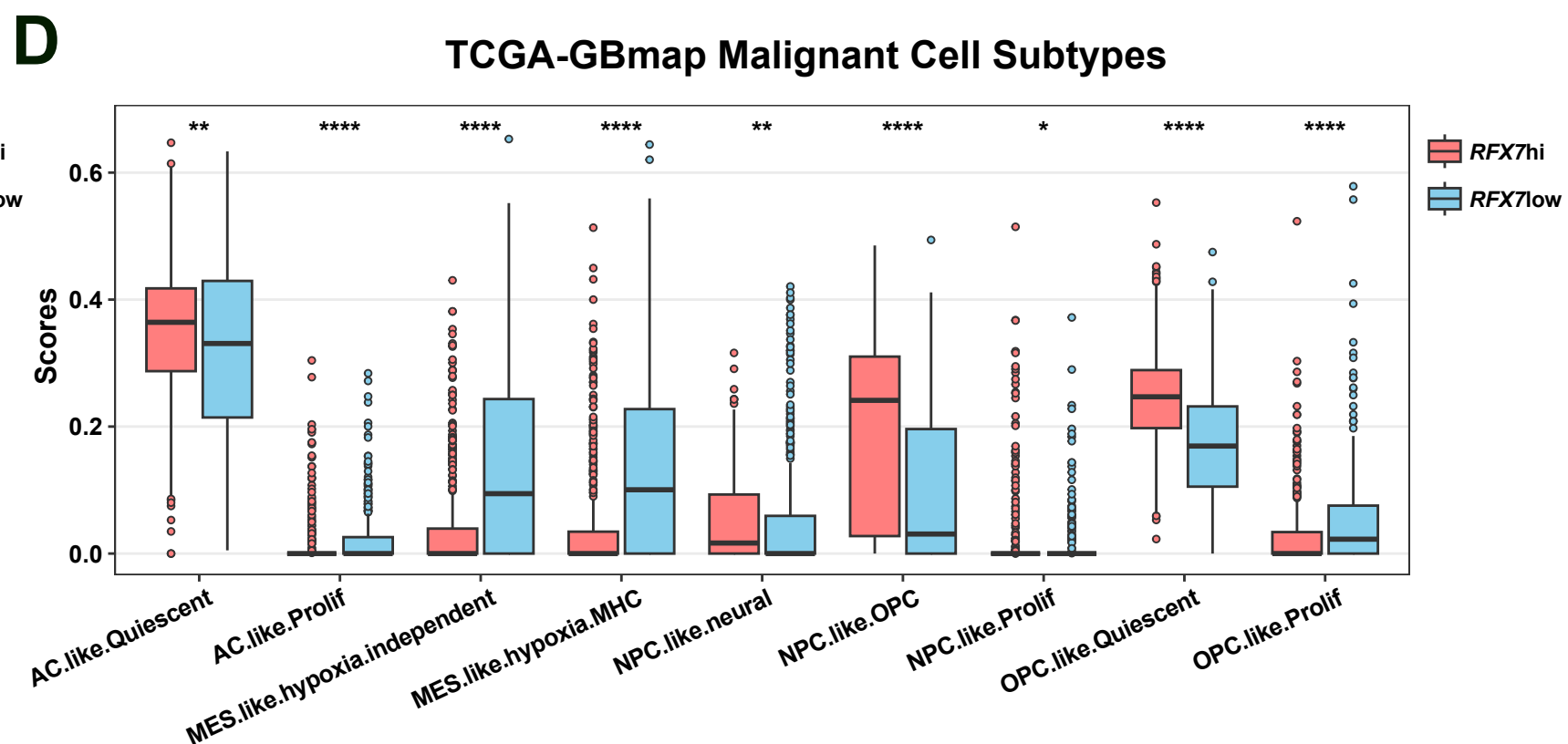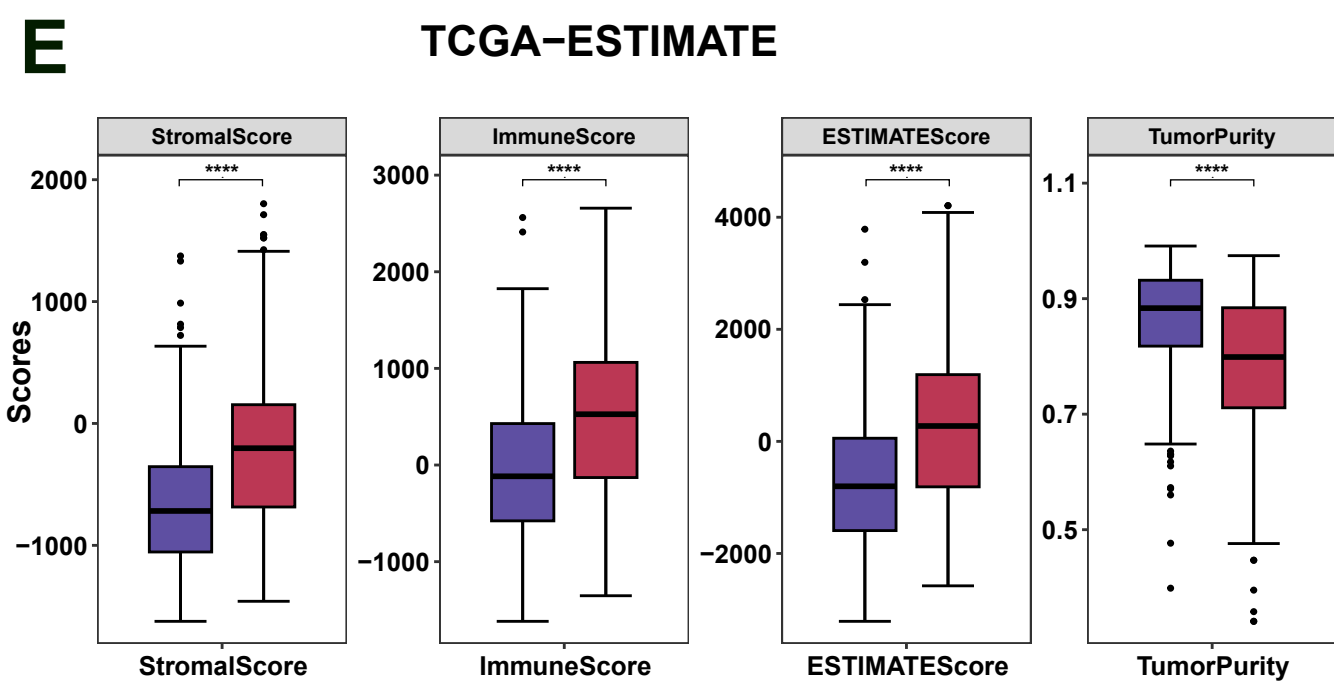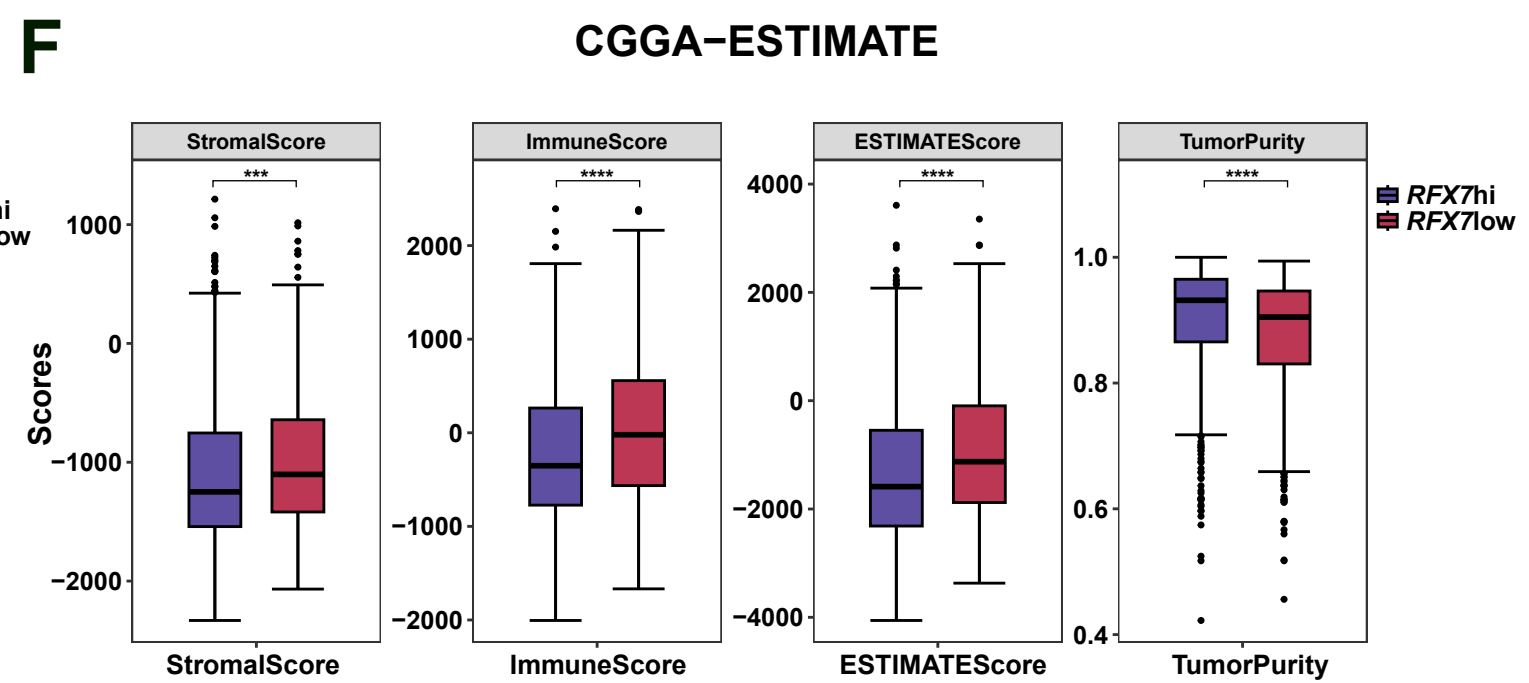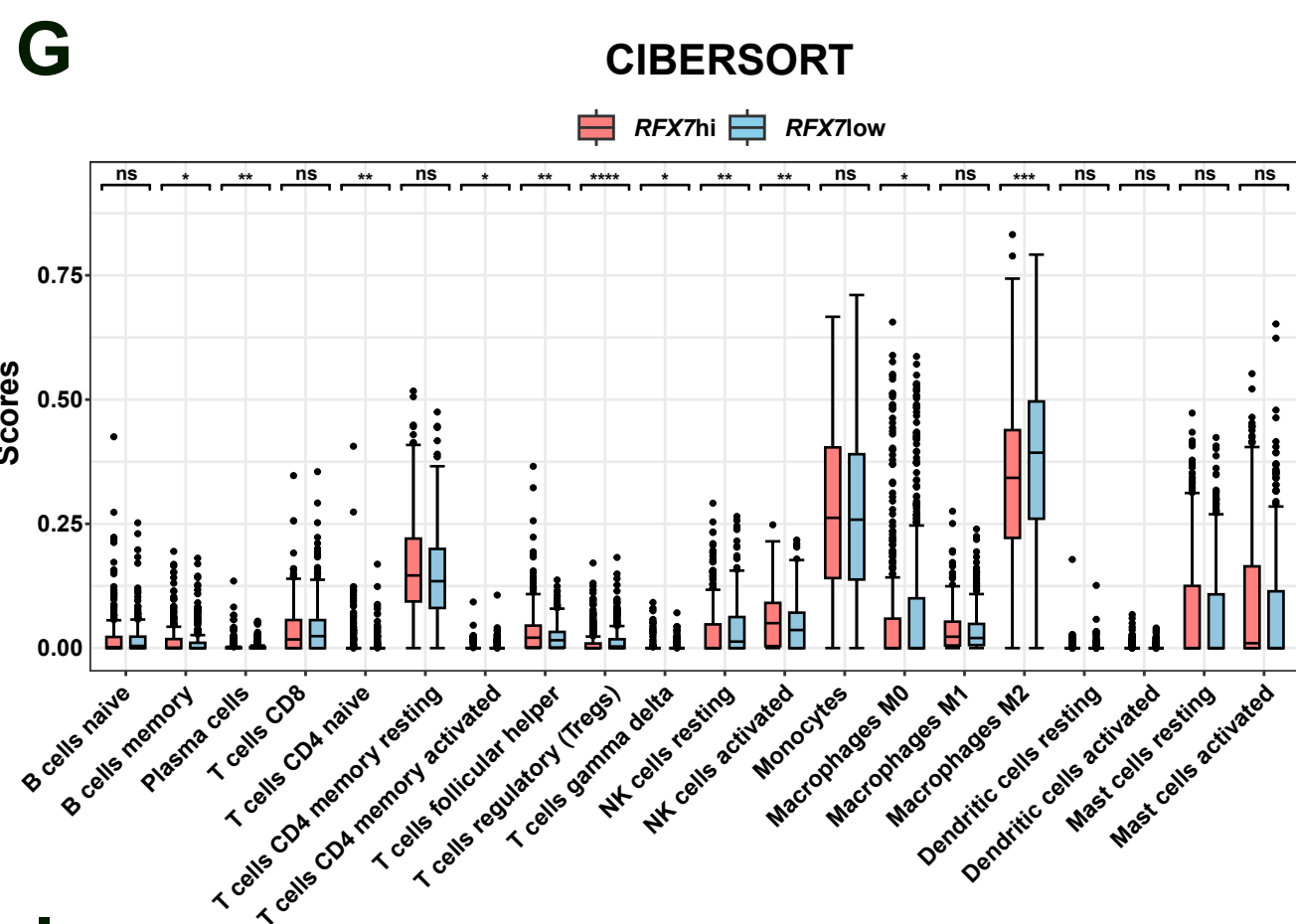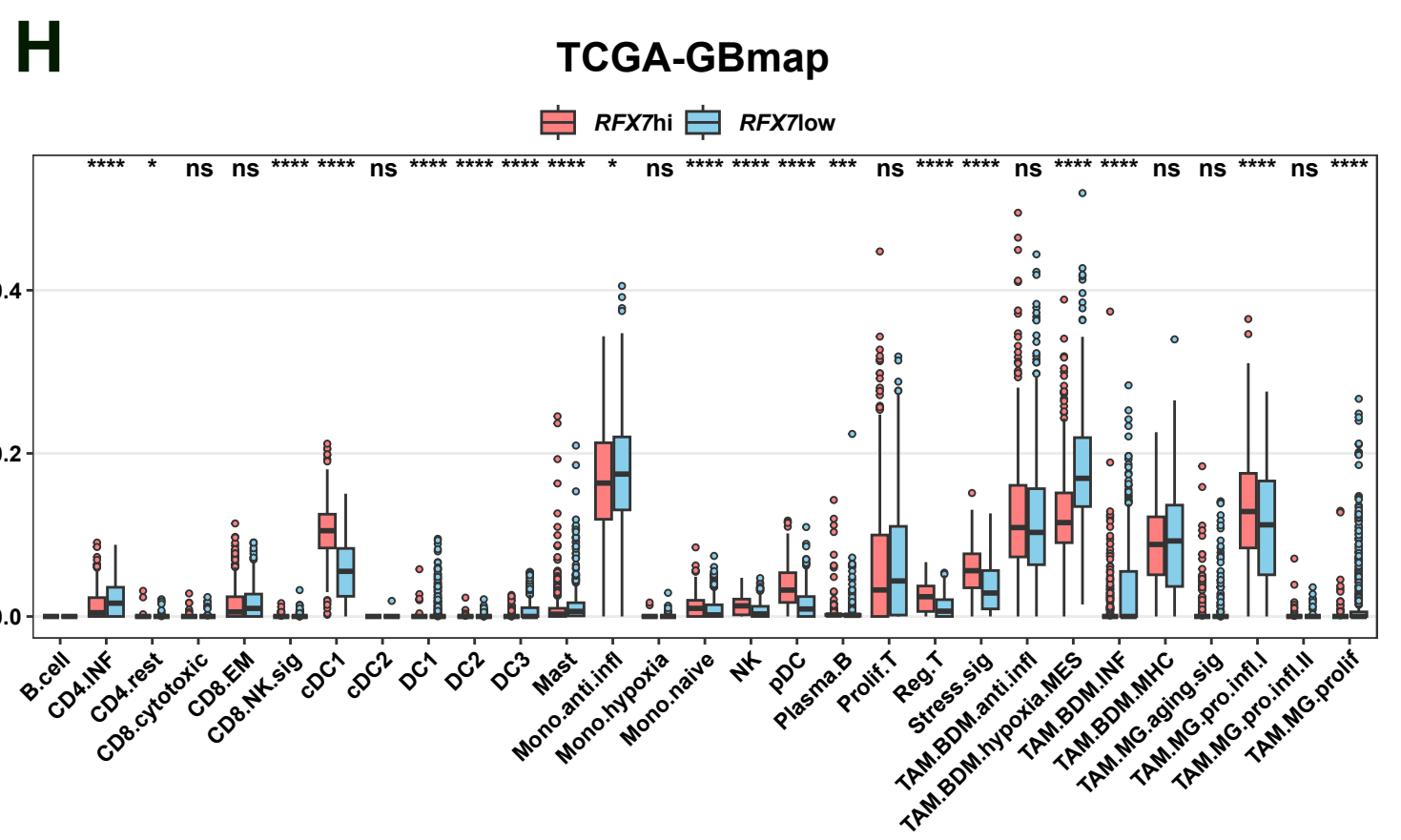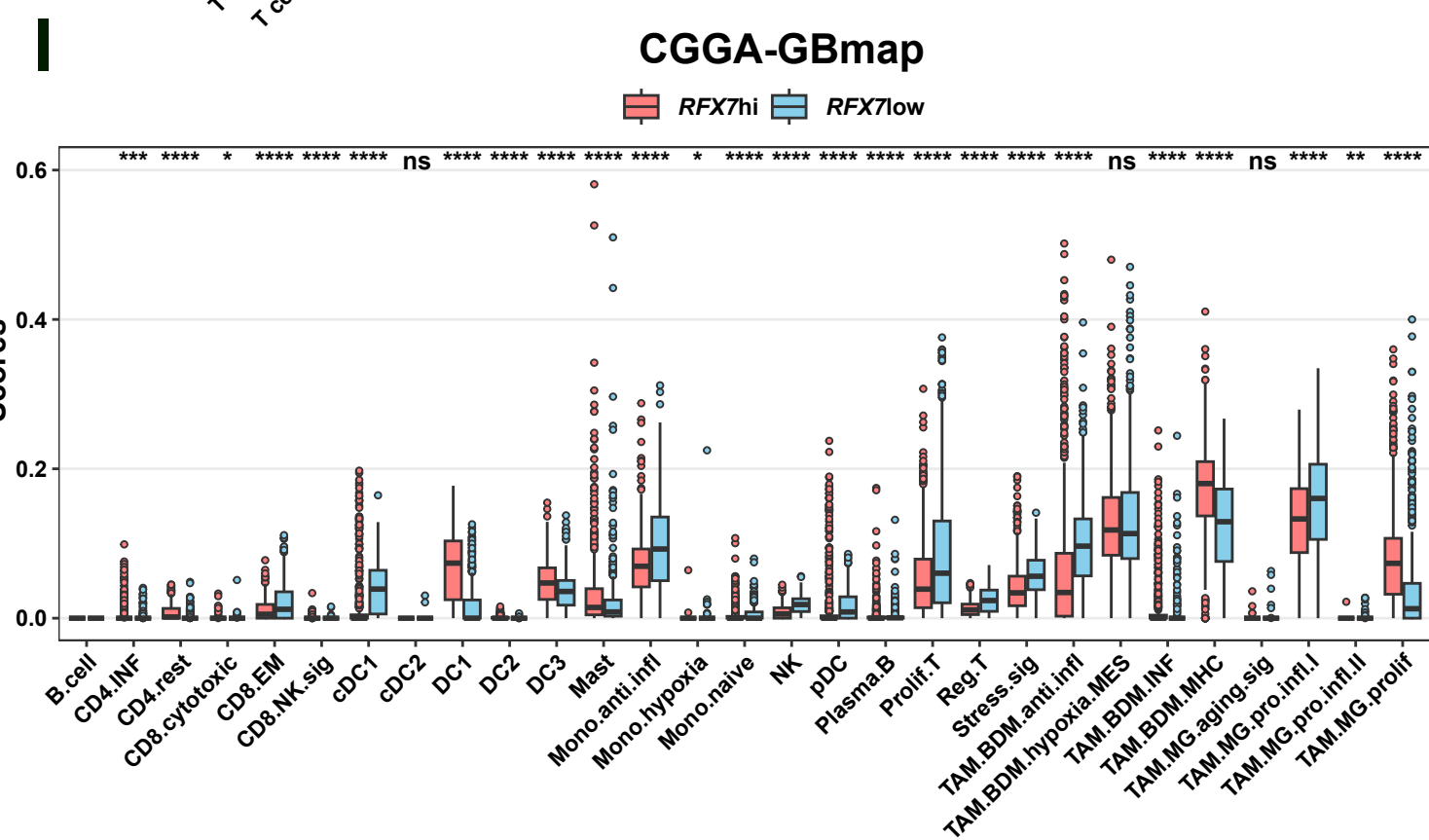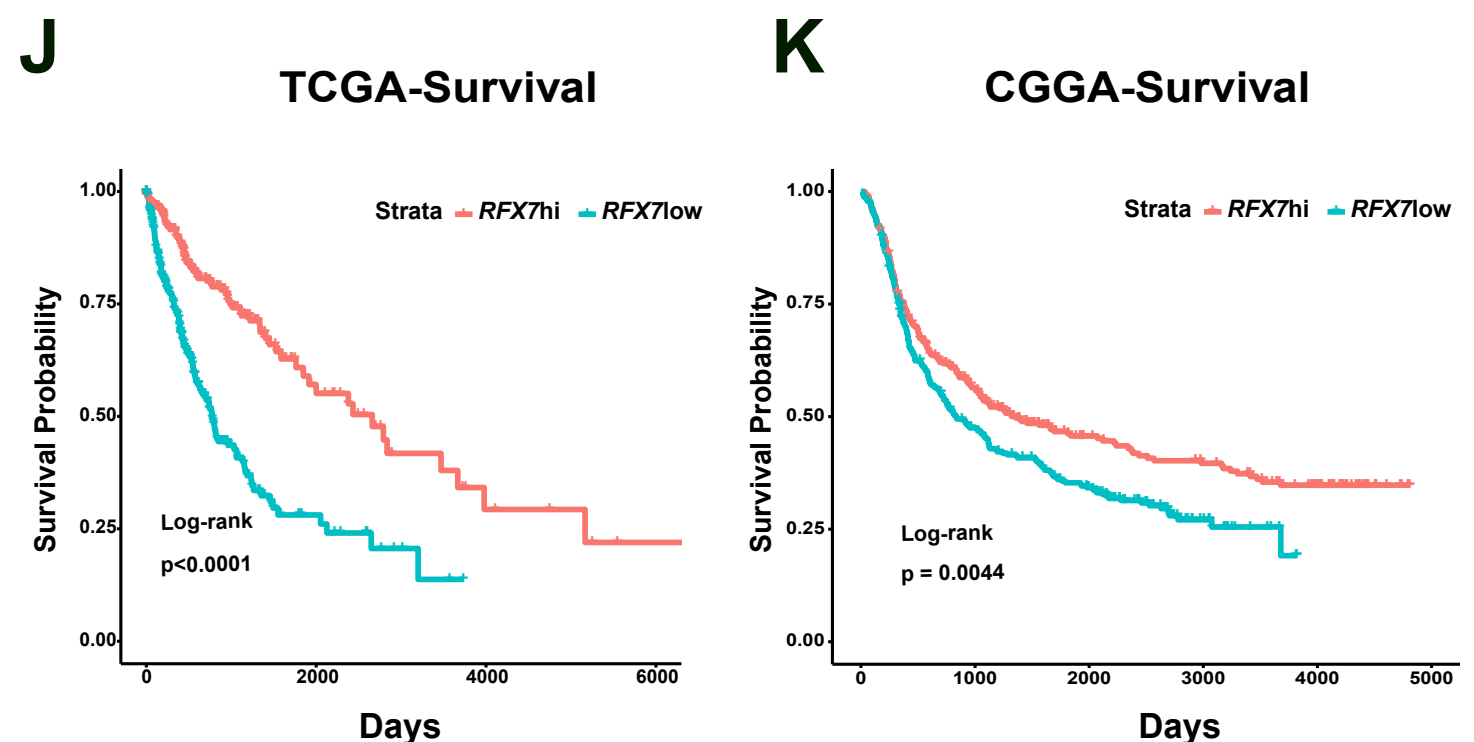

Supplement: Supplementary file 2 — Supporting File 2: advs75761‐sup‐0002‐FigureS1.pdf. [file ADVS-9999-e23792-s003.pdf]

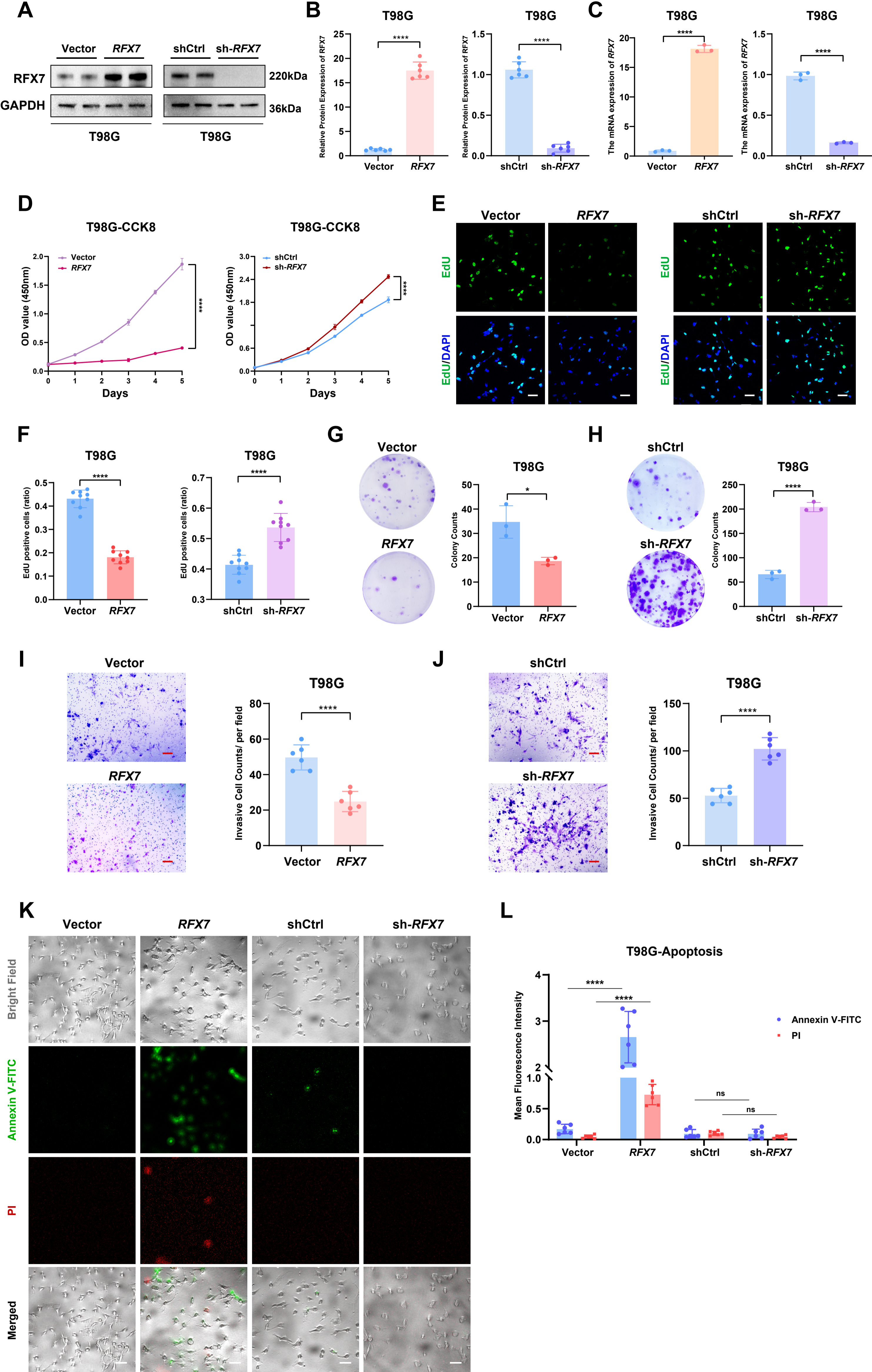

Supplement: Supplementary file 3 — Supporting File 3: advs75761‐sup‐0003‐FigureS2.pdf. [file ADVS-9999-e23792-s008.pdf]

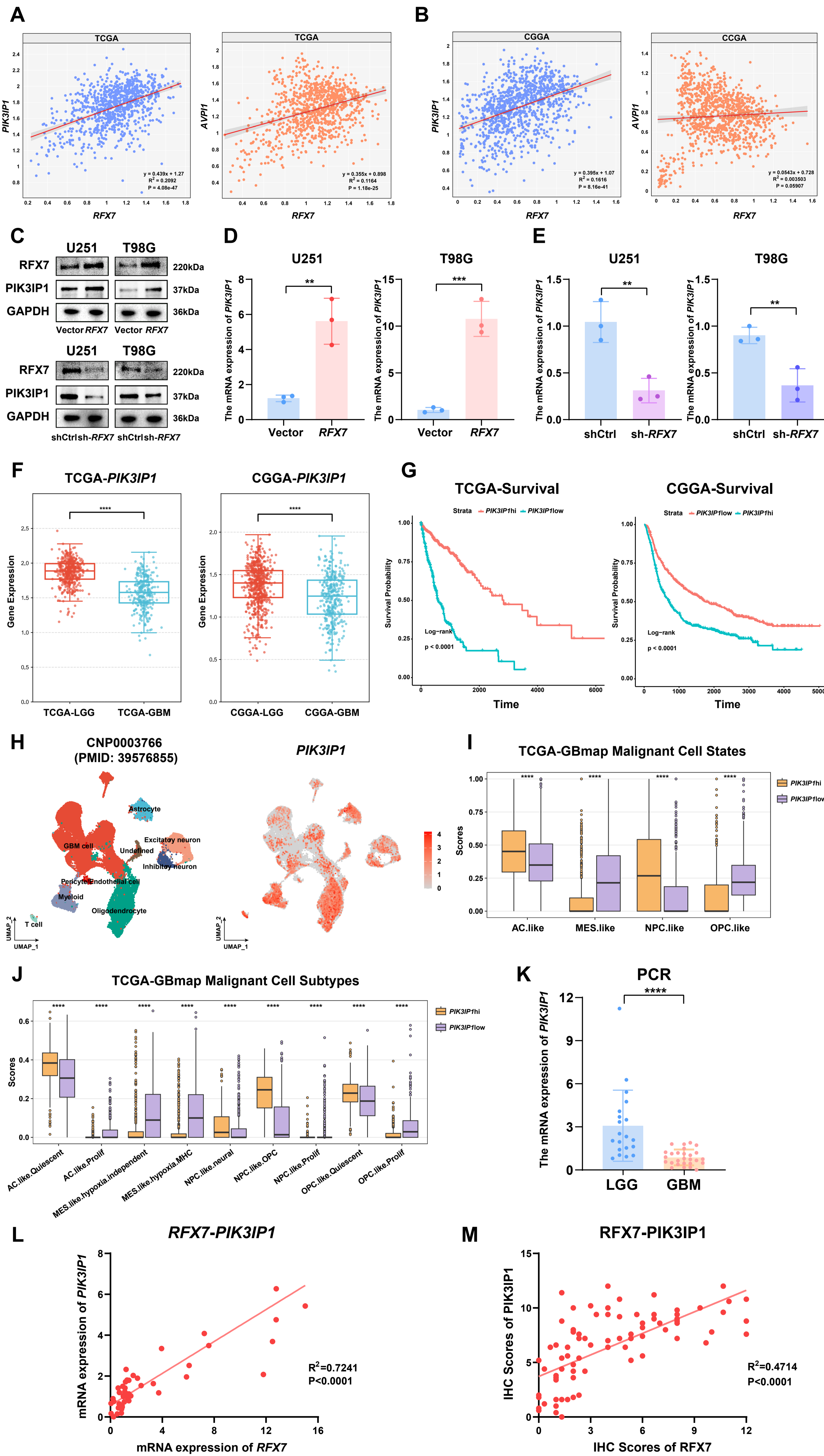

Supplement: Supplementary file 4 — Supporting File 4: advs75761‐sup‐0004‐FigureS3.pdf. [file ADVS-9999-e23792-s001.pdf]

**A****T98G-CCK8**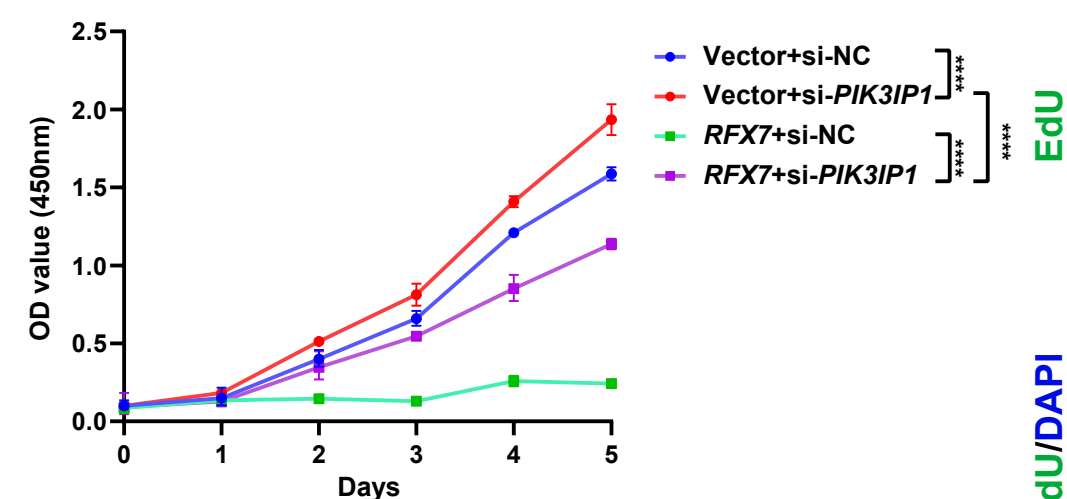**B**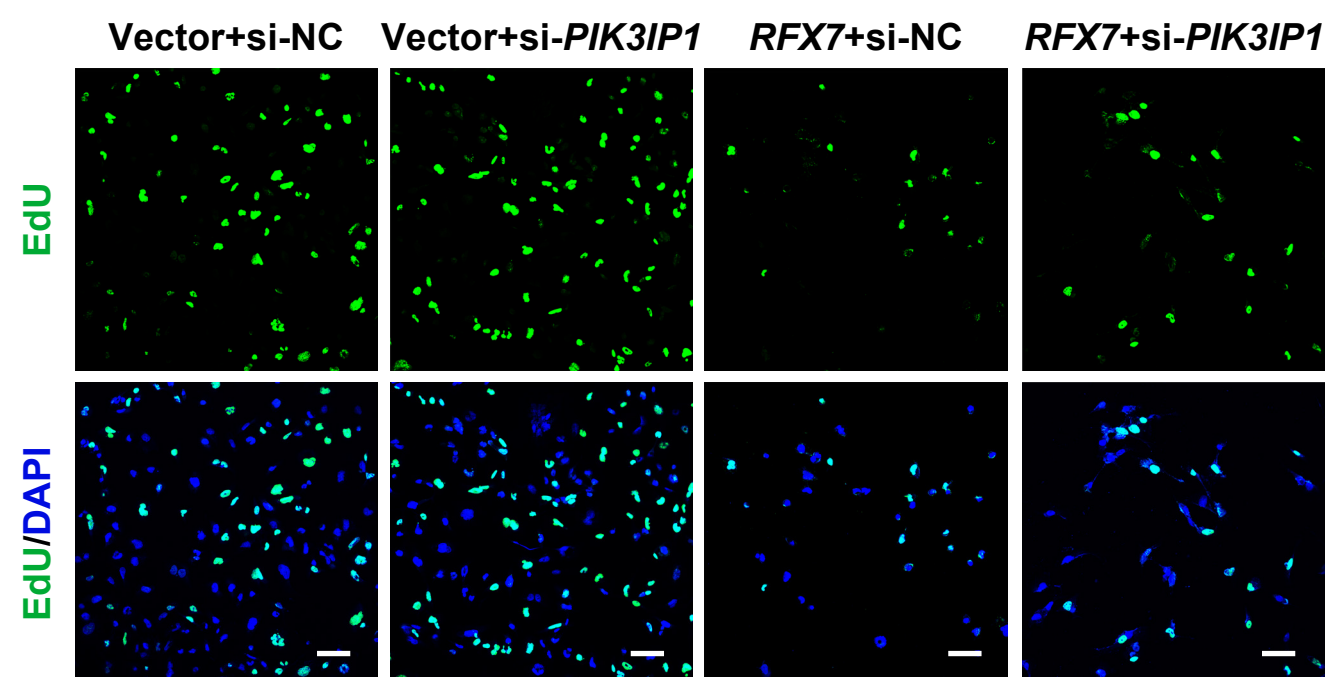**C****T98G-EdU**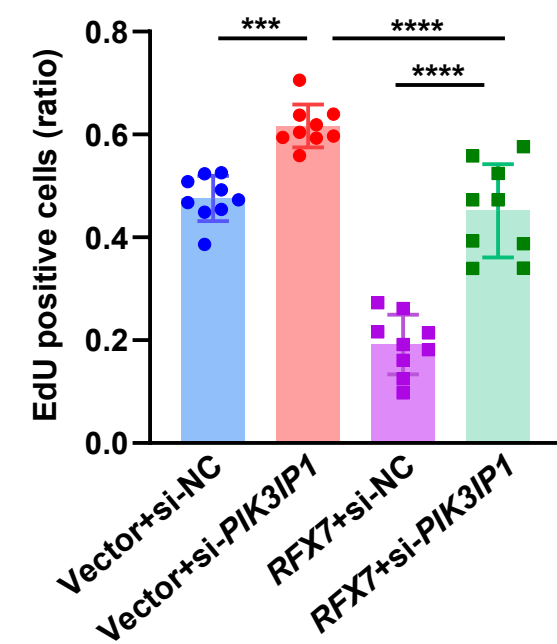**D**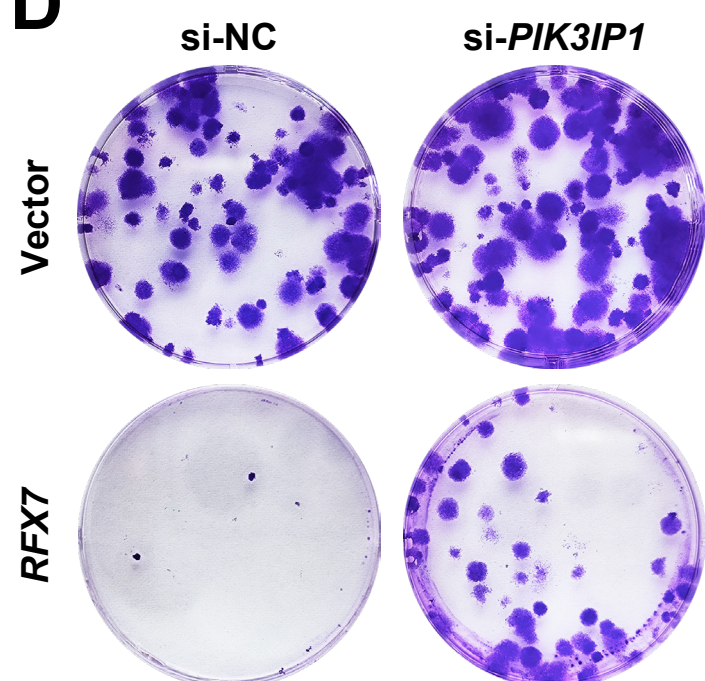**T98G-Colony Formation**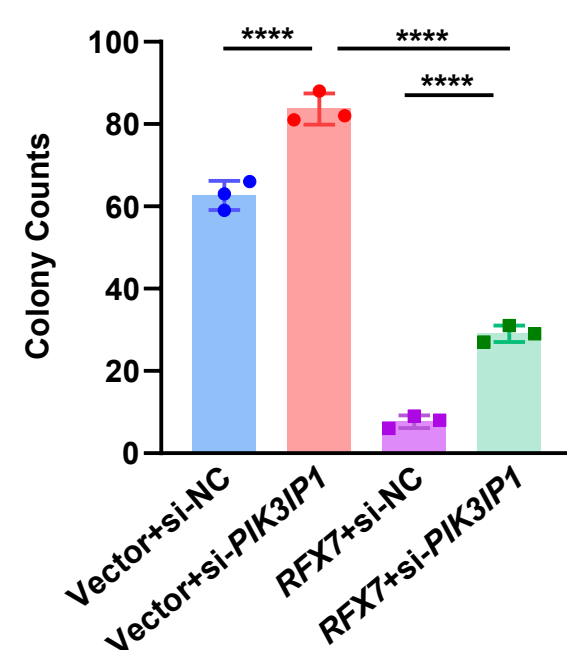**E**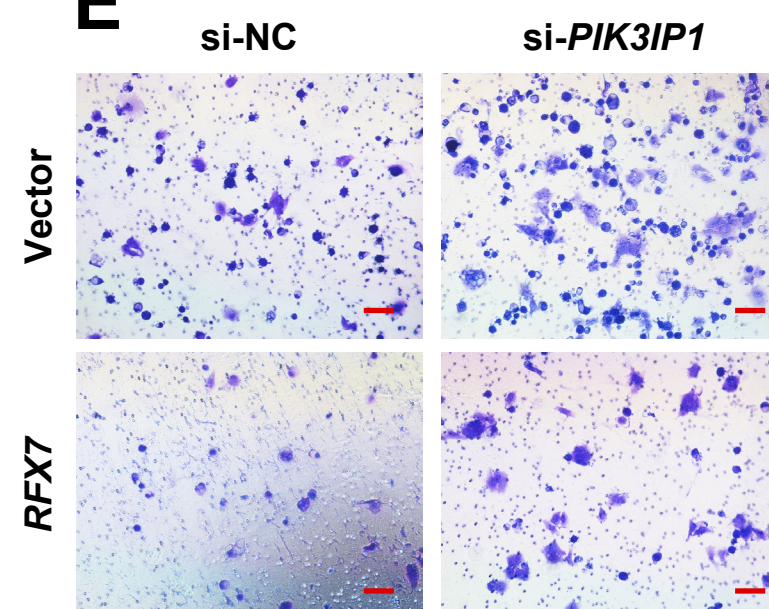**T98G-Transwell**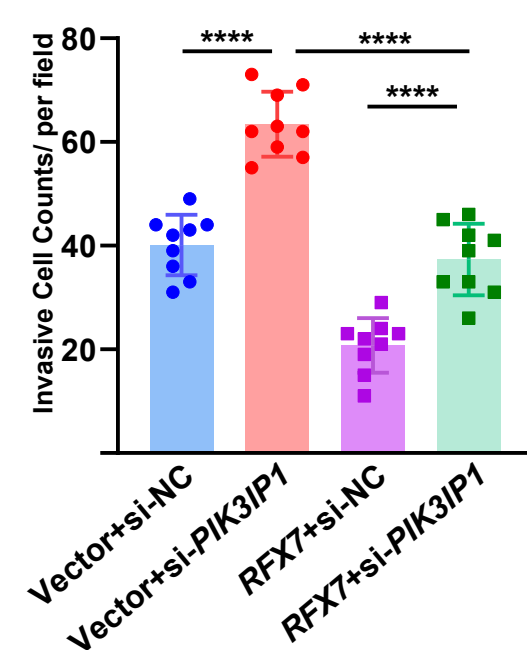**F**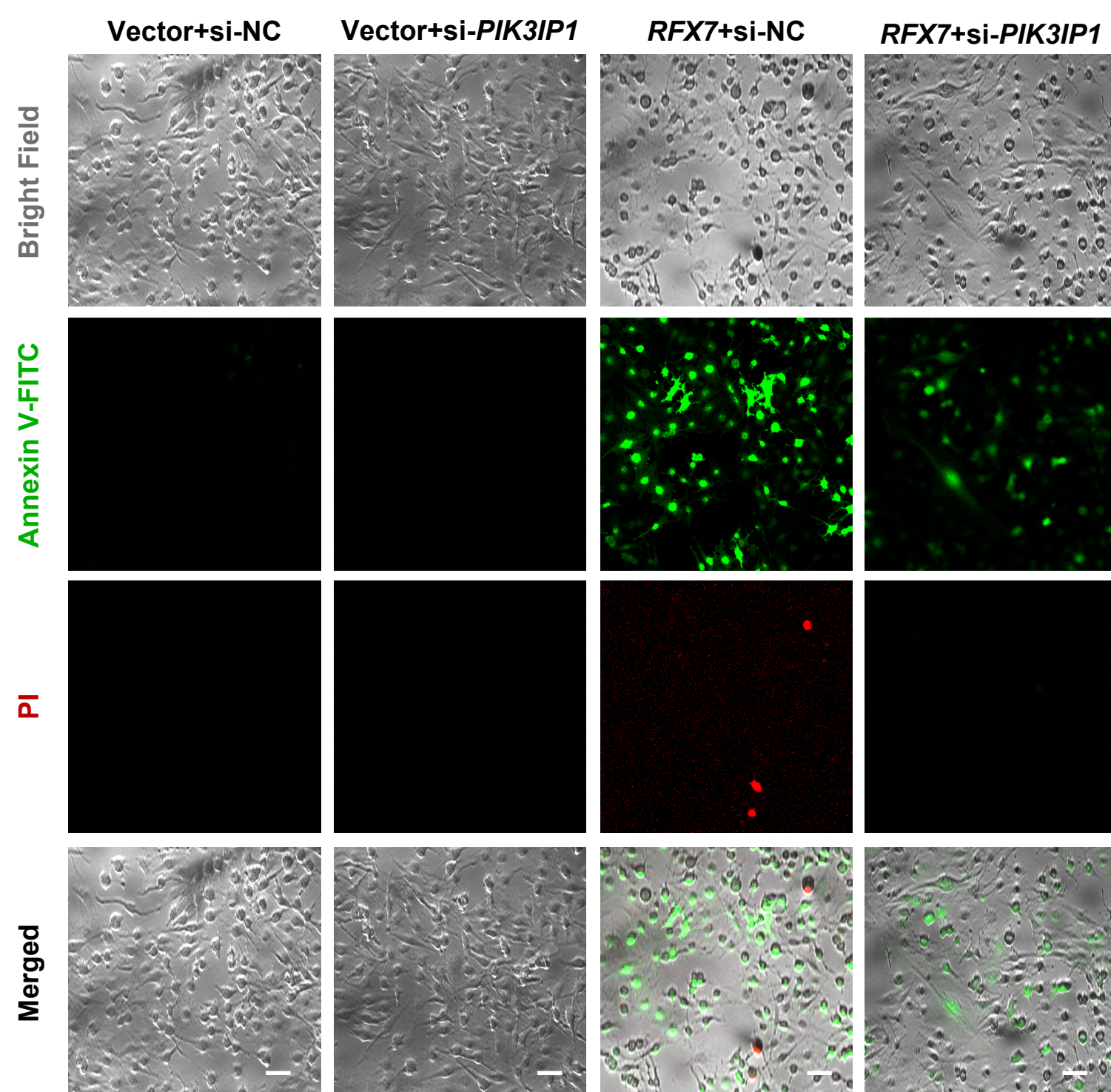**T98G-Apoptosis****G**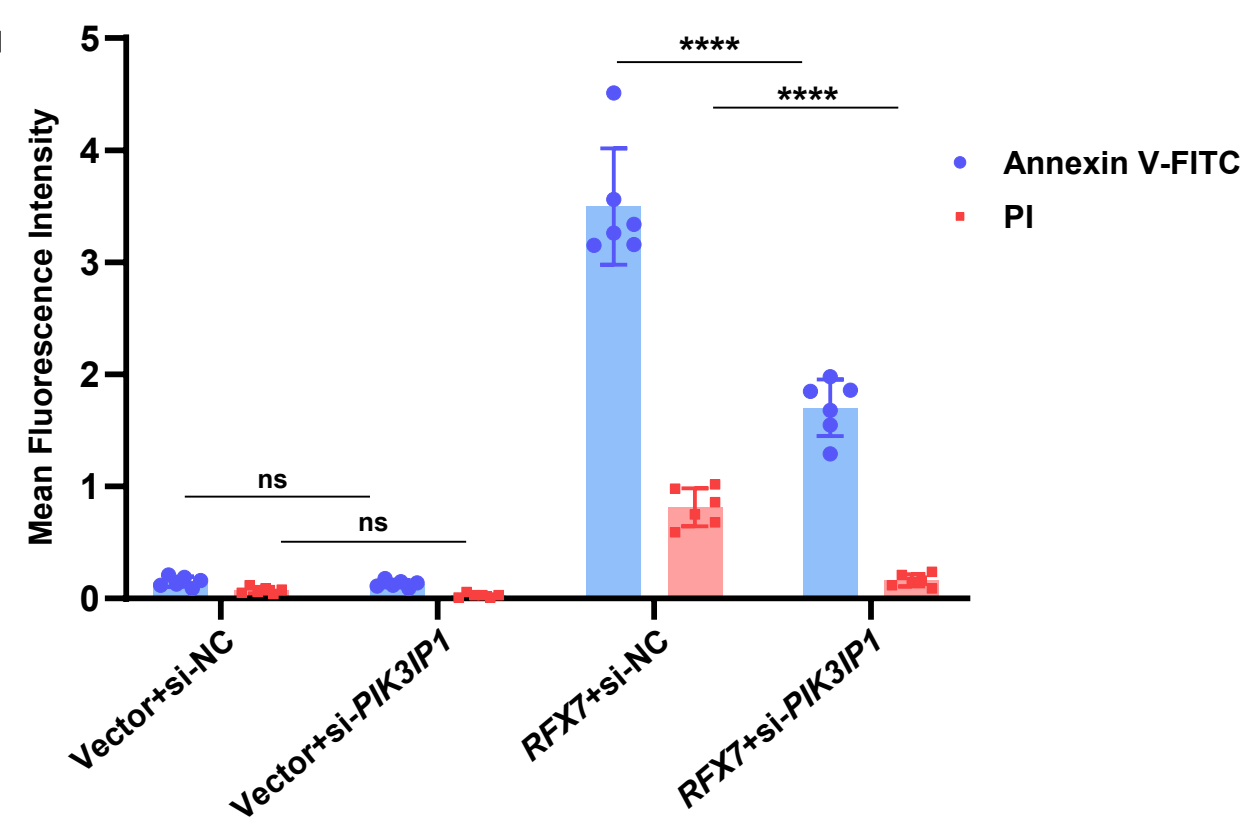**H**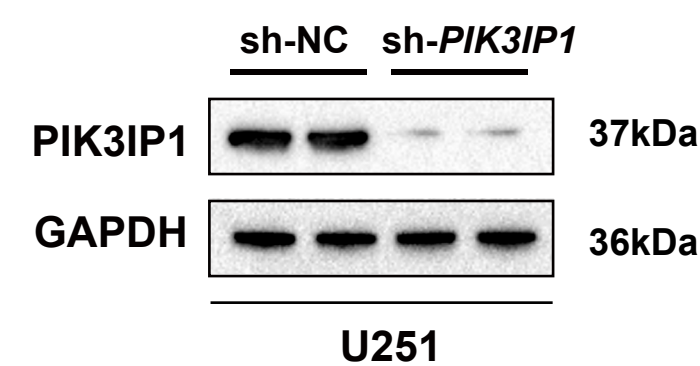**I**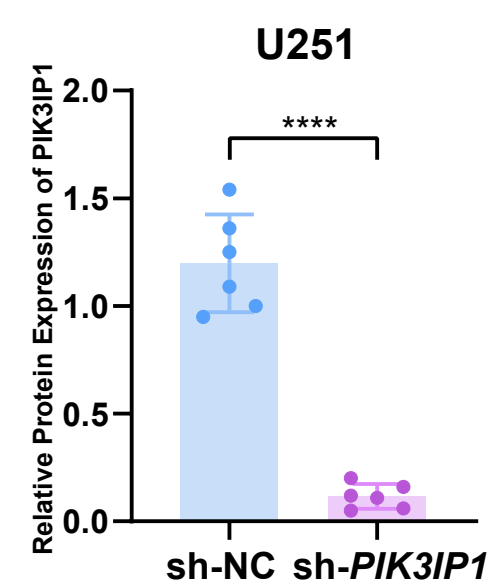**J**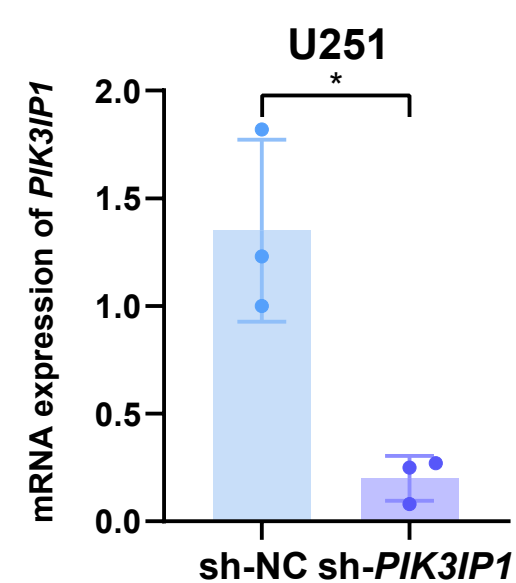

Supplement: Supplementary file 5 — Supporting File 5: advs75761‐sup‐0005‐FigureS4.pdf. [file ADVS-9999-e23792-s006.pdf]

**A****T98G-CCK8**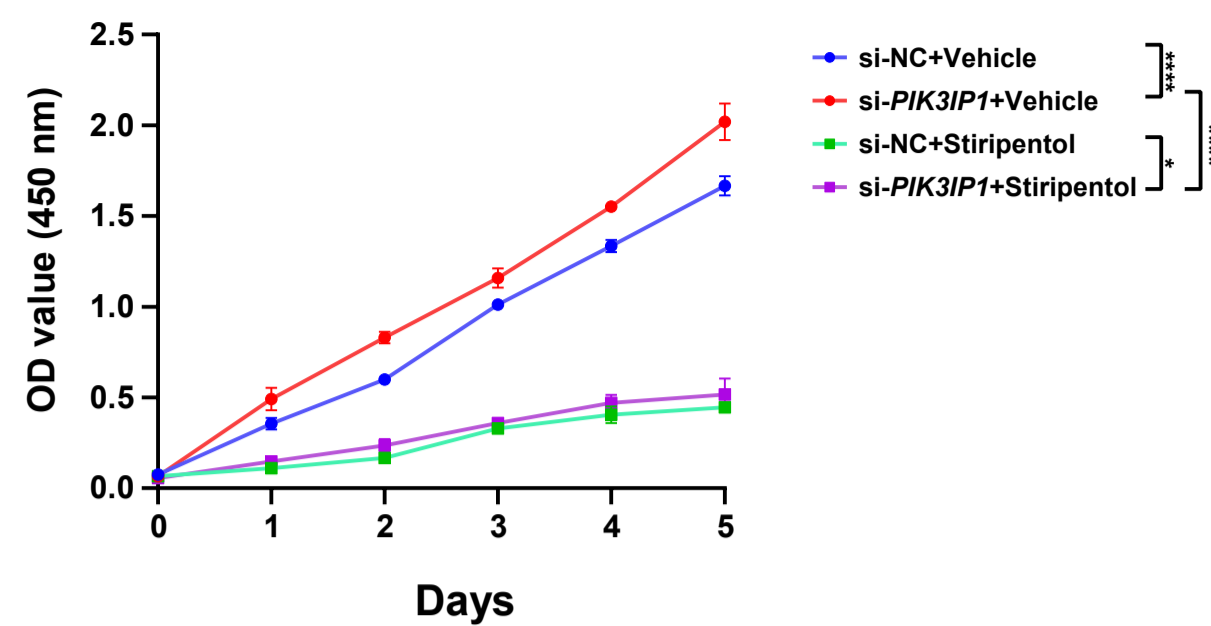**B**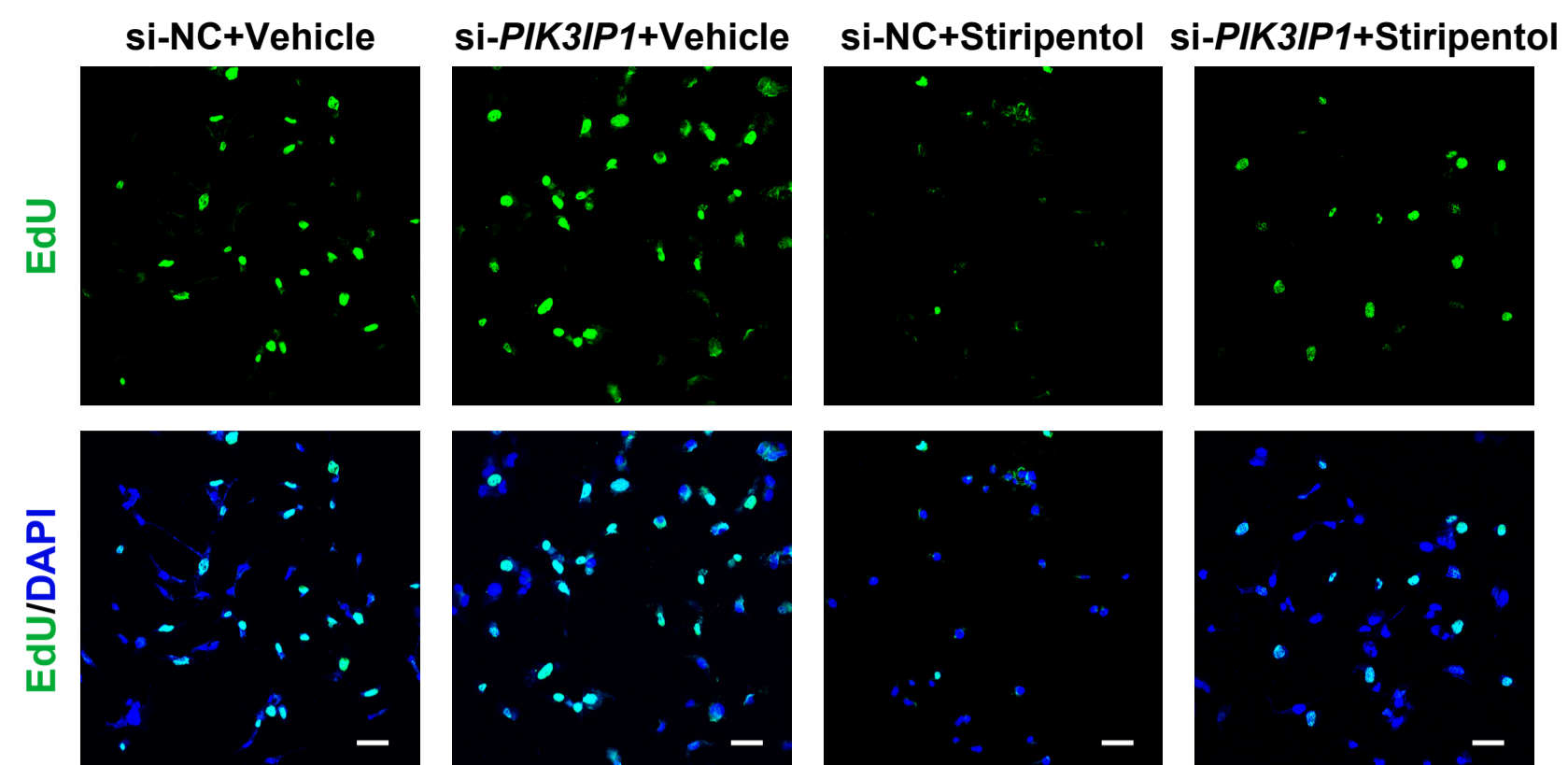**C****T98G-EdU**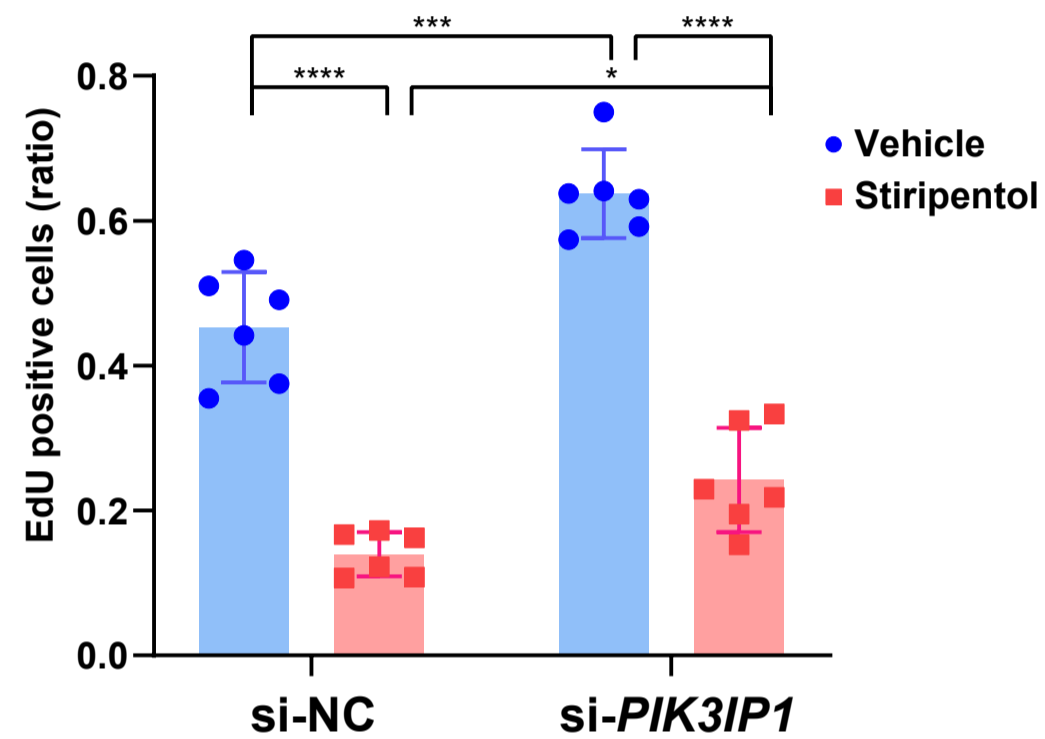**D**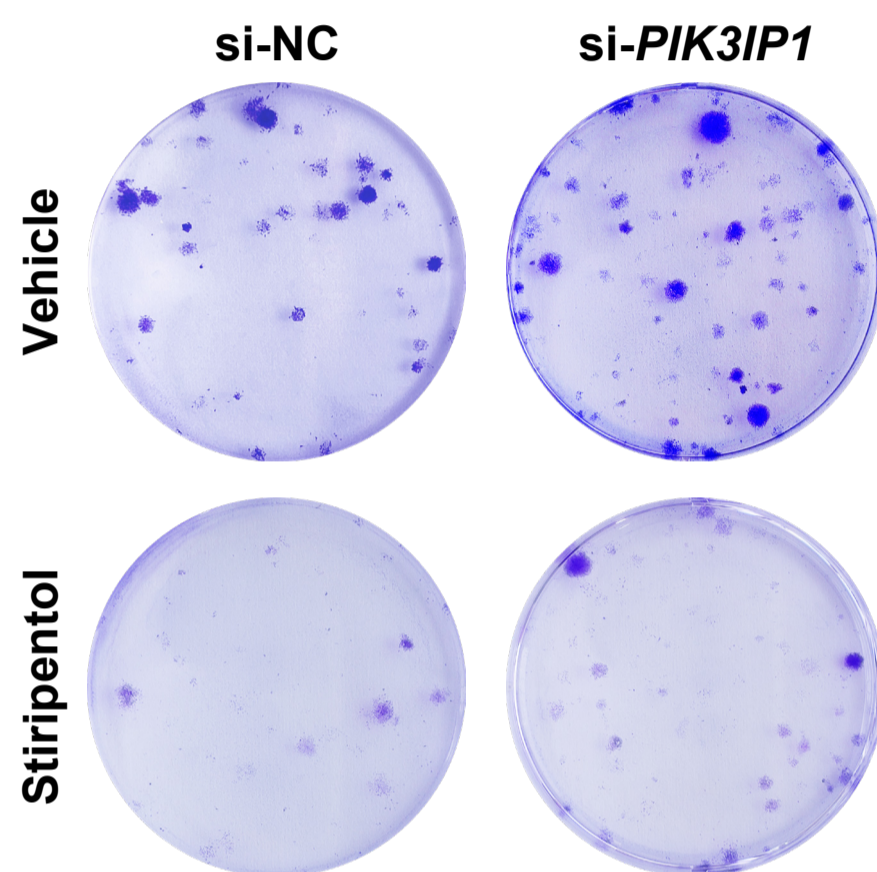**E****T98G-Colony Formation**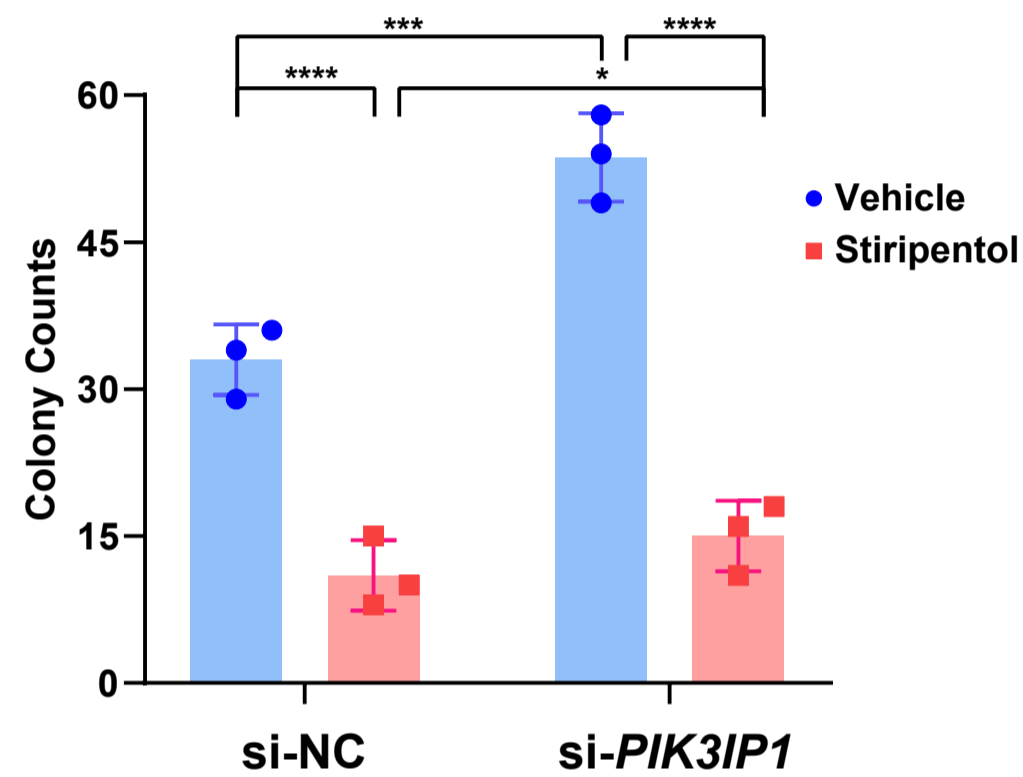**F**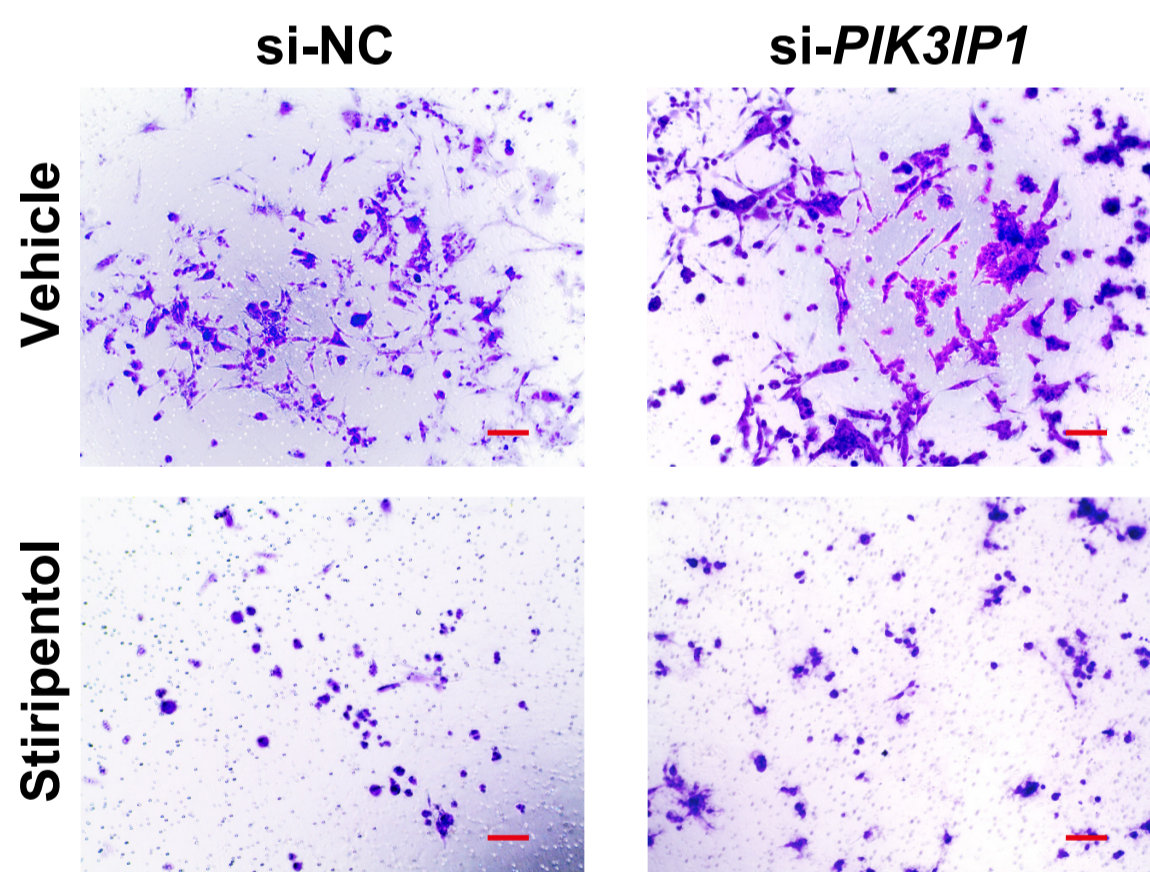**G****T98G-Transwell**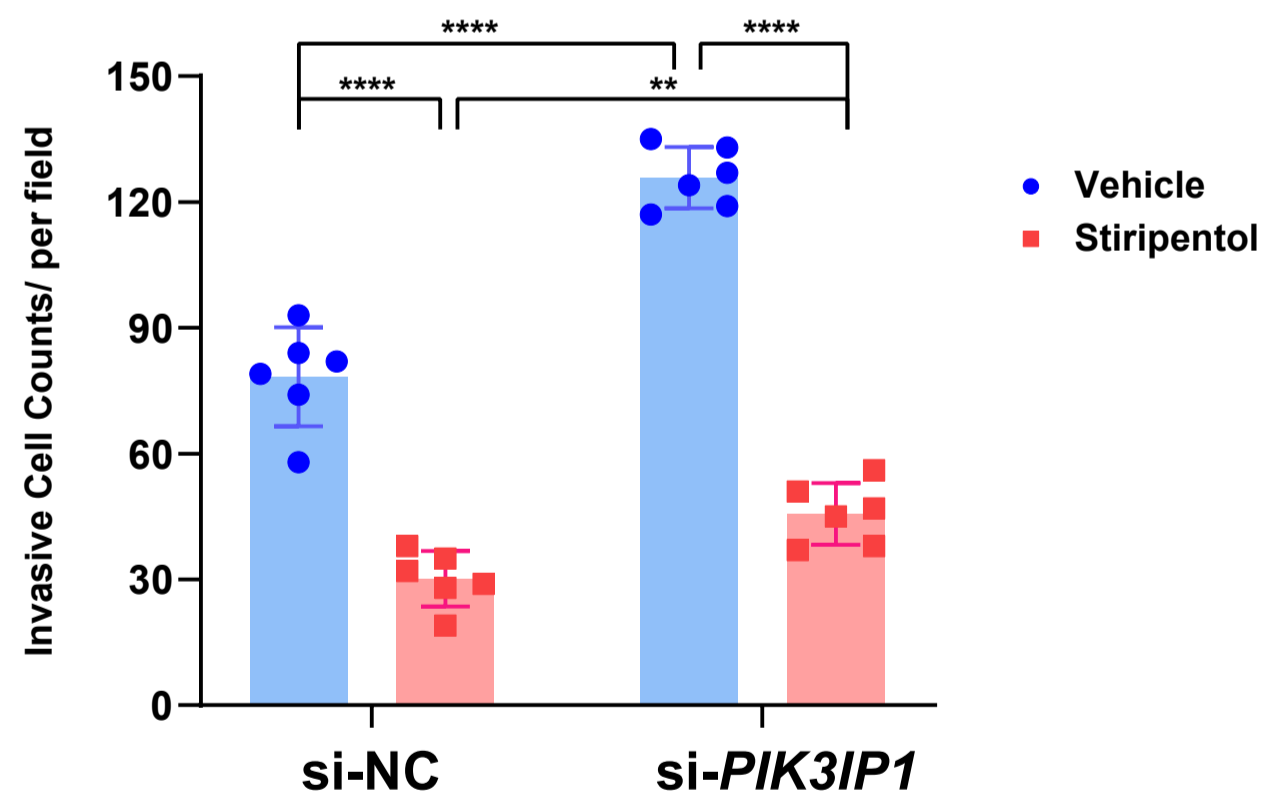**H**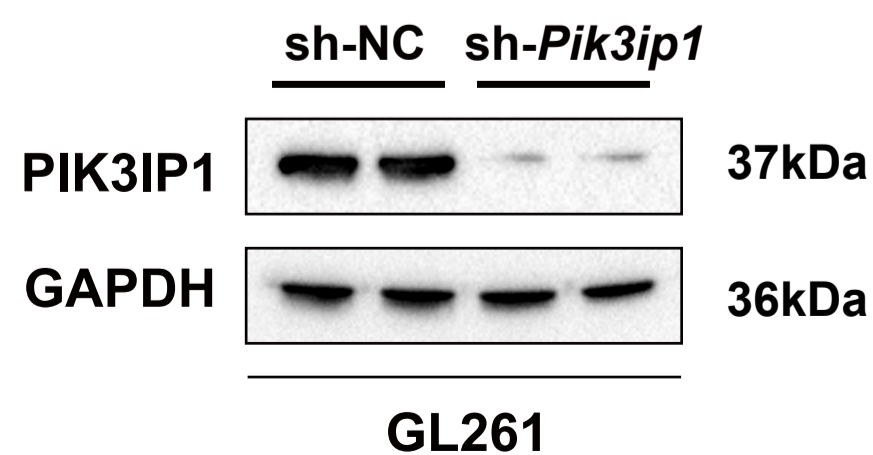**I**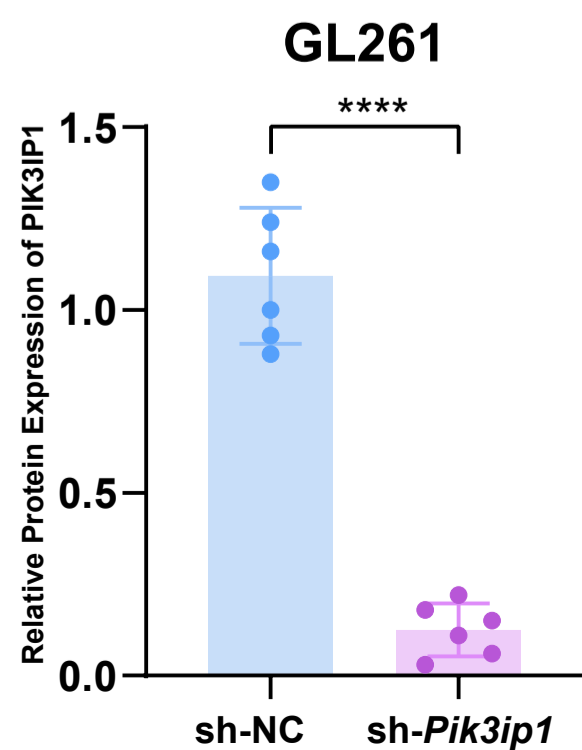**J**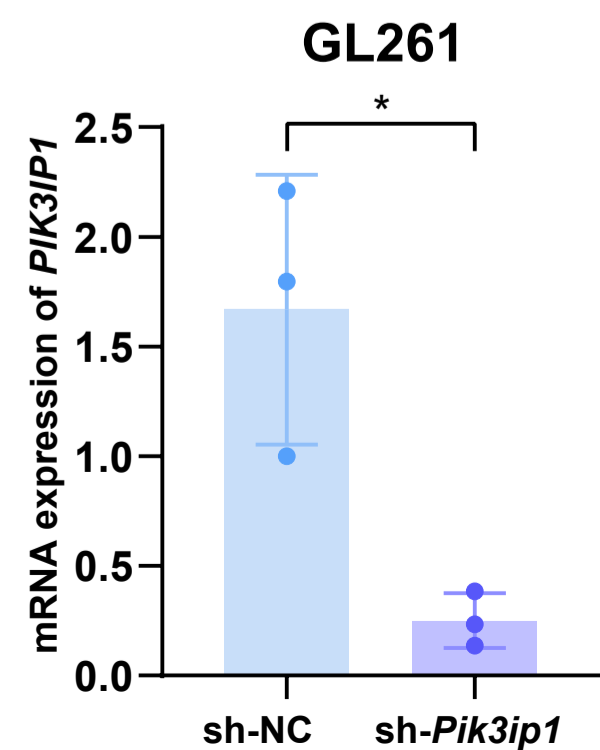

Supplement: Supplementary file 8 — Supporting File 8: advs75761‐sup‐0008‐FigureS7.pdf. [file ADVS-9999-e23792-s005.pdf]
