# Supplementary material for: Epigenetic Silencing of RFX7 Defines a Transcriptional Axis Linking Lactate Metabolism to Immune Checkpoint Therapy in Glioblastoma
Source: Adv Sci (Weinh). 2026 May 28:e23792. Online ahead of print. doi: 10.1002/advs.202523792 (PMC13336137; doi:10.1002/advs.202523792)
Supplement: Supplementary file 6 — Supporting File 6: advs75761‐sup‐0006‐FigureS5.pdf. [file ADVS-9999-e23792-s002.pdf]

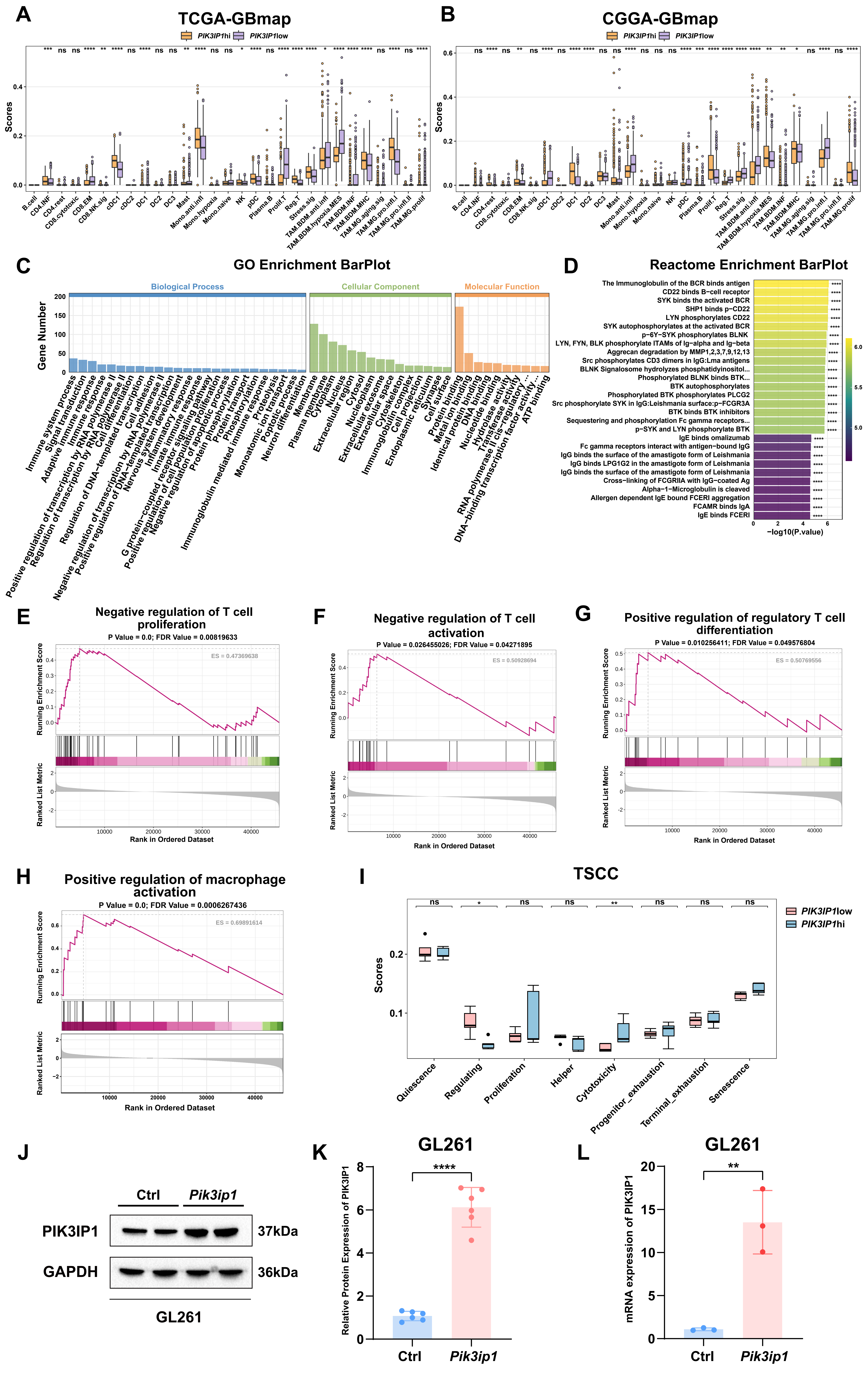

A
TCGA-GBmap
PIK3IP1hi
PIK3IP1low
Scores
B.cell
CD4.INF
CD4.rest
CD8.cytotoxic
CD8.EM
CD8.NK.sig
cDC1
cDC2
DC1
DC2
DC3
Mast
Mono.anti.infl
Mono.hypoxia
Mono.naive
NK
pDC
Plasma.B
Prolif.T
Reg.T
Stress.sig
TAM.BDM.anti.infl
TAM.BDM.hypoxia.MES
TAM.BDM.INF
TAM.BDM.MHC
TAM.MG.aging.sig
TAM.MG.pro.infl.I
TAM.MG.pro.infl.II
TAM.MG.prolif
B
CGGA-GBmap
PIK3IP1hi
PIK3IP1low
Scores
C
GO Enrichment BarPlot
Biological Process
Cellular Component
Molecular Function
Gene Number
Immune system process
Signal transduction
Adaptive immune response
Immune response
Positive regulation of transcription by RNA polymerase II
Regulation of transcription by RNA polymerase II
Cell differentiation
Regulation of DNA-templated transcription
Cell adhesion
Negative regulation of transcription by RNA polymerase II
Positive regulation of DNA-templated transcription
Nervous system development
Inflammatory response
Innate immune response
G protein-coupled receptor signaling pathway
Positive regulation of cell population proliferation
Negative regulation of apoptotic process
Protein phosphorylation
Protein transport
Phosphorylation
Immunoglobulin mediated immune response
Proteolysis
Monoatomic ion transport
Apoptotic process
Neuron differentiation
Membrane
Plasma membrane
Cytoplasm
Nucleus
Extracellular region
Cytosol
Nucleoplasm
Extracellular exosome
Extracellular space
Cytoskeleton
Immunoglobulin complex
Cell projection
Endoplasmic reticulum
Synapse
Cell surface
Protein binding
Metal ion binding
Identical protein binding
DNA binding
Nucleotide binding
Hydrolase activity
Transferase activity
RNA polymerase II cis-regulatory ...
DNA-binding transcription factor activity ...
ATP binding
D
Reactome Enrichment BarPlot
The Immunoglobulin of the BCR binds antigen
CD22 binds B-cell receptor
SYK binds the activated BCR
SHP1 binds p-CD22
LYN phosphorylates CD22
SYK autophosphorylates at the activated BCR
p-6Y-SYK phosphorylates BLNK
LYN, FYN, BLK phosphorylate ITAMs of Ig-alpha and Ig-beta
Aggrecan degradation by MMP1,2,3,7,9,12,13
Src phosphorylates CD3 dimers in IgG:Lma antigens
BLNK Signalosome hydrolyzes phosphatidyinositol...
Phosphorylated BLNK binds BTK...
BTK autophosphorylates
Phosphorylated BTK phosphorylates PLCG2
Src phosphorylate SYK in IgG:Leishmania surface:p-FCGR3A
BTK binds BTK inhibitors
Sequestering and phosphorylation Fc gamma receptors...
p-SYK and LYN phosphorylate BTK
IgE binds omalizumab
Fc gamma receptors interact with antigen-bound IgG
IgG binds the surface of the amastigote form of Leishmania
IgG binds LPG1G2 in the amastigote form of Leishmania
IgG binds the surface of the amastigote form of Leishmania
Cross-linking of FCGRIIA with IgG-coated Ag
Alpha-1-Microglobulin is cleaved
Allergen dependent IgE bound FCERI aggregation
FCAMR binds IgA
IgE binds FCERI
-log10(P.value)
E
Negative regulation of T cell proliferation
P Value = 0.0; FDR Value = 0.00819633
ES = 0.47369638
Running Enrichment Score
Ranked List Metric
Rank in Ordered Dataset
F
Negative regulation of T cell activation
P Value = 0.026455026; FDR Value = 0.04271895
ES = 0.50928694
Running Enrichment Score
Ranked List Metric
Rank in Ordered Dataset
G
Positive regulation of regulatory T cell differentiation
P Value = 0.010256411; FDR Value = 0.049576804
ES = 0.50769556
Running Enrichment Score
Ranked List Metric
Rank in Ordered Dataset
H
Positive regulation of macrophage activation
P Value = 0.0; FDR Value = 0.0006267436
ES = 0.69891614
Running Enrichment Score
Ranked List Metric
Rank in Ordered Dataset
I
TSCC
PIK3IP1low
PIK3IP1hi
Scores
Quiescence
Regulating
Proliferation
Helper
Cytotoxicity
Progenitor_exhaustion
Terminal_exhaustion
Senescence
J
Ctrl
Pik3ip1
PIK3IP1
37kDa
GAPDH
36kDa
GL261
K
GL261
Relative Protein Expression of PIK3IP1
****
Ctrl
Pik3ip1
L
GL261
mRNA expression of PIK3IP1
**
Ctrl
Pik3ip1
